# Detection, Quantification and Molecular Characterization of Fowl Adenoviruses Circulating in Ecuadorian Chicken Flocks during 2019–2021

**DOI:** 10.3390/vetsci10020115

**Published:** 2023-02-03

**Authors:** Silvana H. Santander-Parra, Manuel Caza, Luis Nuñez

**Affiliations:** 1 Facultad de Ciencias de la Salud, Carrera de Medicina Veterinaria, Universidad de Las Américas (UDLA), Quito 170124, Ecuador; 2 One Health Research Group, Facultad de Ciencias de la Salud, Carrera de Medicina Veterinaria, Universidad de Las Américas (UDLA), Quito 170124, Ecuador

**Keywords:** fowl adenovirus, Ecuador, qPCR, enteric disease

## Abstract

**Simple Summary:**

Viral diseases cause high economic losses in poultry. One of them is IBH (inclusion body hepatitis) and HHS (hepatitis-hydropericardium syndrome) associated with fowl adenoviruses (FAdVs), a virus that spreads rapidly by feces. Thus, the aim of the present study was to detect, quantify, and identify the genotypes of FAdVs that are circulating in the chicken commercial flocks and could be responsible for outbreaks of hepatic diseases. The study showed the presence of genotypes FAdV-2/D2, FAdV-6/E1, FAdV-8a/E2, and FAdV-11/D3 of FAdV, all of them related to IBH and HHS. These results showed that in commercial birds with enteric problems, FAdV is circulating, pointing to the need for revising biosecurity programs and considering FAdV as being disseminated by feces and causing IBH and HHS.

**Abstract:**

Fowl adenoviruses are a group of pathogens that cause large economic losses worldwide in the poultry industry, in addition to producing a wide range of diseases, such as IBH, HHS, and enteric and respiratory diseases. The objective of this study was to quantify, identify, and molecularly characterize the types of FAdV circulating in commercial poultry farms (broilers, breeders, and layers) in Ecuador from 2019 to 2021. Molecular characterization was performed by PCR, quantification by qPCR, and subsequent sequencing for each positive sample. The results indicated that the FAdV genotypes circulating in our country are FAdV-2/D2, FAdV-6/E1, FAdV-8a/E2, and FAdV-11/D3; the samples were grouped into different groups that contain sequences that were obtained from countries in Africa, Asia, and America, and that are found in birds at different ages, since early age where can cause different clinical signs, such as diarrhea, ruffled feathers and dwarfism. Therefore, these results indicate that several genotypes of the virus are circulating in commercial poultry flocks, suggesting that biosecurity measures on farms should be improved, in addition to carrying out new or improved vaccination plans.

## 1. Introduction

Avian adenoviruses are a group of nonenveloped viruses with double-stranded, linear DNA genomes and with icosahedral geometry; their diameters are 70–90 nm and their sizes range from 35–45 kb [1]. They belong to the Adenoviridae family [2,3], whose members are classified into five genera: Mastadenovirus, Atadenovirus, Siadenovirus, Aviadenovirus, and Ichtadenovirus [4,5]. Fowl adenoviruses (FAdVs) belong to the genus Aviadenovirus and can be divided into five species: fowl aviadenovirus A, fowl aviadenovirus B, fowl aviadenovirus C, fowl aviadenovirus D, and fowl aviadenovirus E, and based into 12 genotypes [6] which mostly correspond to well-established serotypes FAdV-1 to 8a and 8b to 11 [1]. These FAdVs are distributed worldwide and have been detected in various geographical locations [7,8,9,10,11,12,13,14]. Infected birds can present multiple clinical signs, such as reduced egg production, respiratory problems, arthritis, uneven growth, or enteritis, with varying degrees of mortality [12,15].

The transmission occurs mainly via oral route; however, vertical transmission is an important form of contamination due to the poorly developed immune system of off-spring [16], making FAdVs infections spread quickly and highlighting the importance of early diagnosis and the determination of the genetic features of the virus. For this reason, different viral detection methods have been developed over time [6,17,18,19].

Due to the global distribution of this virus’ serotypes, the different effects they cause in chickens, and the high rates of enteric diseases frequently reported by Ecuadorian poultry farmers (data not published), it is essential to know which genotypes are circulating in our country, given that poultry farming in Ecuador is one of the main economic activities in the country—the main provinces where the largest number of poultry farms are located are Pichincha, El Oro, Guayas, Imbabura, and Manabí. This study aimed to quantify, identify, and molecularly characterize the FAdVs circulating in commercial poultry farms (broilers, breeders, and layers) in Ecuador during 2019–2021 to make better decisions and prevent its circulation in birds and to develop rigorously applied biosecurity measures to control its transmission.

## 2. Materials and Methods

### 2.1. Samples

For the present study, from 2019 to 2021, 221 samples of enteric tracts obtained from broiler chickens (BC), layer hens (LH), and breeder hens (BH) showing signs of enteric disease, such as ruffled feathers, dwarfism, stunting, cloacal empasting, and diarrhea from four provinces (e.g., Chimborazo, Imbabura, Pichincha, and Tungurahua) of Ecuador were sent to the research laboratory at the College of Veterinary Medicine of the Universidad de Las Americas, Quito, Ecuador, to determine the presence or absence of fowl adenovirus (FAdV). At necropsy, the intestines (duodenum, jejunum, and ileum) were distended, with gas bubbles, watery contents, and undigested food; furthermore, the intestinal wall was thin. No other organs showed any apparent lesion. Using a molecular approach, the virus was detected, quantified and used to determine the genetic diversity of FAdV circulating in Ecuadorian chicken flocks that could be responsible for outbreaks of HHS and IBH in the country. All procedures conducted in the present investigation were in accordance with the guidelines and the approval of the Committee on the Care and Use of Laboratory and Domestic Animal resources of the Agency of Regulation and Control of Phytosanitary and Animal Health of Ecuador (AGROCALIDAD), under number #INT/DA/019.

### 2.2. DNA Extraction

DNA was extracted from 100 mg of jejunum tissue using the phenol/chloroform method described by Green and Sambrook [20]. The tissue was macerated and incubated for 5 min at 37 °C with 1000 µL of phenol/guanidine thiocyanate solution. Chloroform (200 µL) was added to the solution, mixed and centrifuged (12,000× *g* for 15 min at 4 °C), 750 µL of propanol was added, and the whole solution was stored at −20 °C for 12 h. The solution was centrifuged (12,000× *g* for 15 min at 4 °C), the supernatant was discarded, and the DNA pellet was rinsed with 70% ethanol twice, and in each rinse was centrifuged (12,000× *g* for 15 min at 4 °C). The DNA was dissolved in 30 µL of Tris EDTA (TE) buffer and stored at −20 °C until the molecular tests and DNA sequencing were performed.

### 2.3. qPCR to Detect FAdV

FAdV was detected in the intestinal contents of samples where viral DNA was detected and quantified using the qPCR method described by Gunes [21] with some modifications. DNA was obtained from samples as described above and subjected to qPCR. The reactions were prepared using a mixture containing 10 µL of PowerUp^TM^ SYBR^®^ Green Master Mix (2×) (Applied Biosystems by Thermo Fisher Scientific, Vilnius, Lithuania), 0.5 µM of each primer (Table 1), 1 µL of DNA from each sample and UltraPure™ DNase/RNase-free Distilled dH_2_O (Invitrogen by Life Technologies, Carlsbad, CA, USA) to a final volume of 20 µL. NTCs were prepared by substituting cDNA with dH_2_O. The reactions were cycled using a CFX96^TM^ Touch Real-Time PCR Detection System (Bio-Rad Laboratories, Inc., Hercules, CA 94547, USA) in standard mode with a hot start step at 95 °C for 2 min, followed by 40 cycles at 95 °C for 15 s, 60 °C for 15 s, and 72 °C for 1 min. A dissociation curve (melting) was performed in three steps: 95 °C for 15 s followed by a decrease in temperature to 60 °C for 1 min and gradual temperature increase (0.3 °C) up to 95 °C. All samples were tested in duplicate and absolute quantifications were conducted relative to the standard curves used in each run. Two non-template controls were placed in each run.

To determine the sensitivity of the assays, endpoint PCR was performed to amplify the region of the 52K and pIIIa genes of FAdV using the method previously described by Gunes [21]. The obtained amplicons were purified using CleanSweep PCR Purification (Applied Biosystems by Thermo Fisher Scientific, Carlsbad, CA, USA) according to the manufacturer’s instructions and quantified using a NanoDrop spectrophotometer (Thermo Fisher Scientific, Carlsbad, CA, USA). Using the web tool DNA copy Number and Dilution Calculator by Thermo Fisher Scientific, the quantity of DNA necessary to prepare a first dilution with a known quantity of DNA copies was calculated and tenfold dilutions were prepared to determine the sensitivities and amplification efficiencies of the qPCR assays. The detection and quantification limits were determined using the standard curve. The limit of detection (LoD) and limit of quantification (LoQ) were determined by the lowest DNA concentration that the assay could quantify that was located in the linear portion of the standard curve.

### 2.4. Molecular Characterization of FAdV

DNA from each sample was also subjected to endpoint PCR for the amplification of a region of the HEXON gene of FAdV using the method described by Meulemans et al., 2001 [17], with some modifications. The extracted DNA (2 µL) from jejunum was added to 23 µL of a mixture containing 0.5 µM of each primer (Table 1), 2.5 µL of 10× buffer, 4 µL of 1.25 mM dNTPs, 37.5 mM MgCl_2_, and 1.0 U of Platinum Taq DNA polymerase (Invitrogen by Thermo Fisher Scientific, Carlsbad, CA, USA). The reaction was run under the same conditions described by Meulemans et al., 2001 [17].

A portion of the HEXON gene was amplified and purified with CleanSweep PCR Purification (Applied Biosystems by Thermo Fisher Scientific, Carlsbad, CA, USA) according to the manufacturer’s instructions. Each purified amplicon was sequenced with forward and reverse primers using a BigDye^®^ Terminator v3.1 Cycle Sequencing kit (Applied Biosystems by Thermo Fisher Scientific, Carlsbad, CA, USA). Sequencing reactions were performed using an ABI 3730 DNA Analyzer (Applied Biosystems by Thermo Fisher Scientific, Carlsbad, CA, USA). The obtained electropherograms were edited using the Geneious 2019.2.1 software package (Biomatters Ltd., Auckland, New Zealand) and analyzed using BLAST to determine the similarities with other sequences deposited in GenBank. The obtained consensus sequence was aligned with other FAdV sequences using the ClustalX 2.2.1 software package, and the similarities of nucleotides and amino acids were inferred in the BioEdit—Sequence Alignment Editor 7.2.5 software package. The phylogenetic tree was built using a neighbor-joining statistics method, together with a p-distance substitution model and phylogeny test bootstrap model with 1000 replicates that were integrated in the MEGA version 7 software package [22].

### 2.5. Statistical Analysis

Descriptive statistics were used to represent the variability of the positive samples using the bird types (e.g., broiler chickens, layer hens, and breeder hens), ages of birds, and geographic distribution of samples. The non-parametric analyses using Kruskal–Wallis rank sum test was carried out with the average of viral gene copies of each group of animal (line birds) in order to determine the degree of significance between them. The correlation analyses between viral gene copies and the age of animal were analyzed. The cutoff for significance was set at a *p*-value ≤ 0.05. All analyses were developed in the R studio package.

### 2.6. GenBank Accession Numbers

The accession numbers of the sequences that were part of the HEXON gene of FAdV obtained were deposited in GenBank with accession numbers UDLA 2 (OQ132955), UDLA 3 (OQ132923), UDLA 4 (OQ132958), UDLA 17 (OQ132924), UDLA 44 (OQ132956), UDLA 50 (OQ132922), UDLA 56 (OQ132921), UDLA 64 (OQ132925), UDLA 71 (OQ132926), UDLA 72 (OQ132957), UDLA 76 (OQ132953), UDLA 76F (OQ132952), UDLA 78 (OQ132954), UDLA 80 (OQ132961), UDLA 85 (OQ132959), UDLA 86 (OQ132927), UDLA 86-2 (OQ132960), UDLA 88 (OQ132951), UDLA 203 (OQ132928), UDLA 207 (OQ132929), UDLA 210 (OQ132930), UDLA 213 (OQ132931), UDLA 222 (OQ132932), UDLA 226 (OQ132933), UDLA 247 (OQ132934), UDLA 250 (OQ132935), UDLA 257 (OQ132936), UDLA 267 (OQ132937), UDLA 268 (OQ132938), UDLA 276 (OQ132939), UDLA 280 (OQ132940), UDLA 282 (OQ132941), UDLA 291 (OQ132942), UDLA 292 (OQ132943), UDLA 292-2 (OQ132944), UDLA 299 (OQ132945), UDLA 303 (OQ132946), UDLA 310 (OQ132947), UDLA 310-2 (OQ132948), UDLA 316 (OQ132949), and UDLA 337 (OQ132950).

## 3. Results

### 3.1. qPCR Assay—Determination of Standard Curve

In the present work, qPCR was used for FAdV detection, and the assay could detect and quantify 10^9^ plasmid copies to 5 DNA copies. LoD and LoQ values were determined in 10 and 5 copies, respectively, of plasmid in 1 µL of DNA. The standard curve showed an efficiency of 100.4%, R^2^ = 0.978, slope = −3.314 and y-int = 40.902. The melting curve was clean, without the presence of additional curves, and showed a specific amplification product with a temperature of 85.5 °C without the presence of nonspecific products.

### 3.2. Detection and Quantification of FAdV

**Figure 1:**
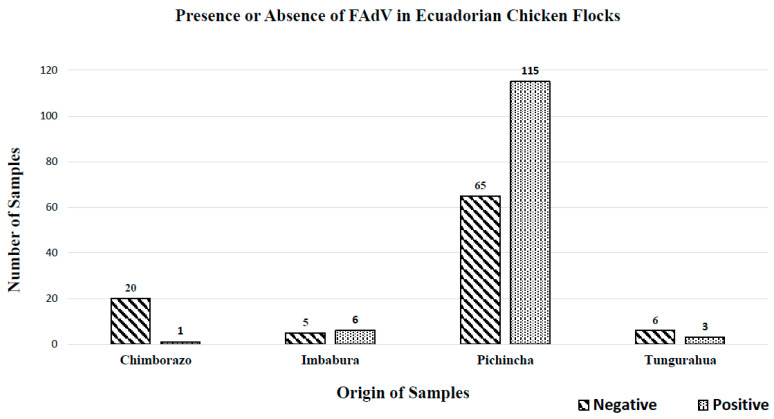
Presence or absence of FAdV in Ecuadorian chicken flocks and virus distributions in the provinces of Ecuador.

FadV was detected in 125 (56.6%) of 221 samples analyzed, belonging to Chimborazo (0.5%), Imbabura (2.7%), Tungurahua (1.4%), and Pichincha (52%) being the one with the greatest number of positive samples (Figure 1).

The virus was detected in BC (91.2%), LH (9%), and BH (2%), with BC having a greater number of positive samples. FAdV was detected from 7 to 42 days of age in BC, in BH at 4 days of age, and LH from 14 to 45 weeks of age (Table 2).

FAdV was detected and quantified in 123 samples and the virus was only detected and not quantified in two samples. The positive samples contained from 393 viral gene copies to 4.6 × 10^6^ viral gene copies. The highest amount of viral gene copies was quantified in BH (4,660,482.12), which was 4 days old, followed by LH (3,601,094.58), which was 40 weeks old, and BC (2,999,651.62), which was 35 days old (Table 2). The analysis of the means of the viral gene copies of each bird linage showed that BC and BH have no statistical significance (*p*-value = 0.990489221), but the means of viral gene copies of both groups were statistically significant (*p*-value = 0.016584108) compared with the LH group. The correlation analysis showed no relation between the amount of viral gene copies and the animal age (*p*-value = 0.182176232).

All samples in which FAdV DNA was detected showed signs of enteric diseases, principally observed in chickens affected with enteric problems mainly related to runting and stunting syndrome (RSS).

### 3.3. Sequencing and Phylogenetic Analyses

In the present study, 41 samples were sequenced and the samples were analyzed using the BLAST tool. High similarities with other FAdV sequences were observed.

**Figure 2:**
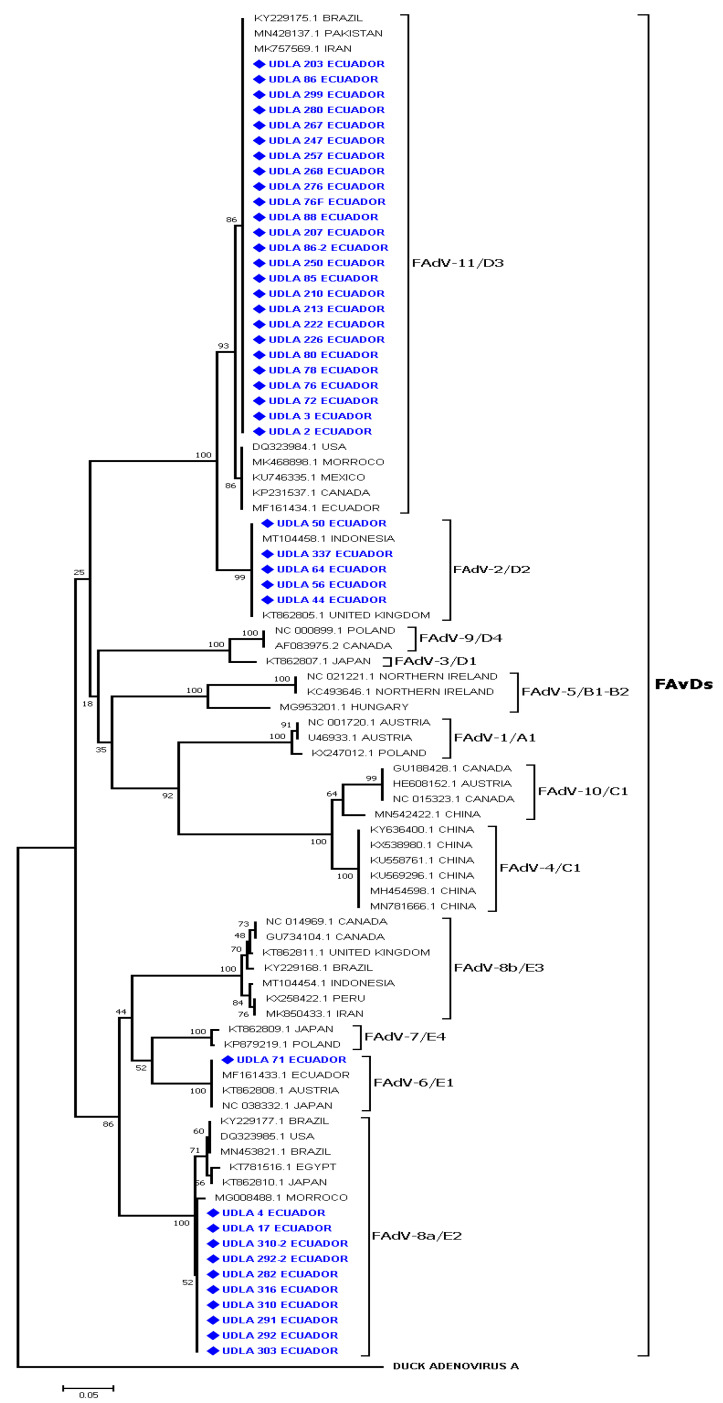
The phylogenetic comparison was performed using the sequences obtained from a portion of the HEXON gene in the present study and other FAdV sequences of many genotypes present in GenBank. Sequences were aligned using the CLUSTAL W method in ClustalX2 2.1. The phylogenetic tree was built using MEGA 7 software. Numbers along the branches refer to bootstrap values for 1000 replicates. The scale bar represents the number of substitutions per site. Duck adenovirus A (NC_001813.1) was used as the outgroup. The FAdV sequences in blue and marked with diamonds were obtained from the jejunum of Ecuadorian chicken flocks exhibiting signs of enteric disorders.

The phylogenetic analyses of a portion of the HEXON gene showed the presence of four genotypes of the virus. These were FAdV-2/D2, FAdV-6/E1, FAdV-8a/E2, and FAdV-11/D3, and the sequences obtained herein were branched in each genotype of FAdV. Samples UDLA 44, 50, 56, 56-1, 64, and 337 formed a group (clade bootstrap value 99%) and were joined with sequences belonging to genotype FAdV-2/D2 from Indonesia and the United Kingdom. The sequences belonging to FAdV-6/E1 grouped with the sequence UDLA 71 in a unique group (clade bootstrap value 100%) and between these groups were sequences from Ecuador, Austria, and Japan. Sequences UDLA 4, 17, 282, 291, 292, 292-2, 303, 310, 310-2, and 316 were clustered in a unique group (clade bootstrap value 93%) with other sequences from Africa (Morocco and Egypt), Asia (Japan), and America (Brazil and the United States) belonging to genotype FAdV-8a/E2. The majority of the sequences [e.g., UDLA 2, 3, 72, 76, 76F, 78, 80, 85, 86, 86-2, 88, 203, 207, 210, 213, 222, 226, 247, 250, 257, 267, 268, 276, 280, and 299)] obtained in this study were clustered with other sequences that were molecularly characterized as genotype FAdV-11/D3 in a unique group (clade bootstrap value 100%) (Figure 2) from America (Canada, Mexico, and the United States), Asia (Pakistan), and Africa (Morocco).

The genotypes of FAdVs were detected in all commercial lines (LH, BH, and BC); genotype FAdV-2/D2 present in the provinces of Chimborazo, Pichincha, Imbabura, and Tungurahua; genotype FAdV-11/D3 present in the province of Pichincha; genotype FAdV-6/E1 present in the province of Tungurahua; and genotype FAdV-8a/E2 present in the provinces of Pichincha and Imbabura (Supplementary Materials).

The present study detected FAdV sequences belonging to genotypes FAdV-2/D2, FAdV-6/E1, FAdV-8a/E2, and FAdV-11/D3. These FAdV-2/D2 sequences have high similarities of nucleotides (NT) of 99.7 to 100% and amino acids (AA) of 100% with a sequence from Indonesia sharing a similarity of 99.7 to 100% of NT and 93.6% of AA. A unique FAdV-6/E1 sequence showed similarities of 99.3 to 100% of NT and 100% of AA with other FAdV-6/E1 sequences from Ecuador, Austria, and Japan. The sequences belonging to genotype FAdV-8a/E2 of FAdV showed similarities of <99.6–100% of NT and 100% of AA, among them and similarities of 98.4 to 99% of NT and 99.5–100% of AA with sequences from Brazil and the United States.

The sequences belonging to genotype FAdV-11/D3 showed similarities of 96.8–100% and 95.1–100% of AA between them and similarities of 98.6–99.9% of NT and 95.6–100% of AA with sequences from Iran, Brazil, the United States, Mexico, and Canada. When comparing the sequences of each genotype obtained in this study, it was observed that genotype FadV-2/D2 was more related to FadV-11/D3 showing differences of 93.1 to 99.5% of AA. However, there were large differences in NT and AA (12.7 to 21.6%) for the sequences of genotypes FadV-6/E1 and FadV-8a/E2. Genotypes FadV-6/E1 and FadV-8a/E2 exhibited large differences in NT and AA (12.7 to 21.6%) with the other genotypes characterized in Ecuador (Supplementary Materials).

## 4. Discussion

Fowl adenoviruses are infectious agents that affect both farmed and wild birds and are distributed throughout the entire world. The diseases they cause include IBH, HHS, adenoviral gizzard erosions (AGE), and respiratory infections, in addition to being associated with enteric diseases [1,23].

Several molecular methods have been used to both detect and characterize adenoviruses [17,19,24,25,26,27]. One of them was developed by Gunes et al., 2012 [21], which allowed us to detect the presence of adenovirus in 56.6% of the samples studied in different avian lineages, broiler chickens were the lineage in which adenovirus was seen in higher percentages (Table 2). Thus, previous studies also indicated that the highest detection rates occurred for this commercial bird lineage, but it is also worth mentioning that adenoviruses were detected in other commercial bird lineages, as in our study, where they were also detected in layer hens [10,11,14]. It was also possible to identify the virus in chickens of a wide variety of ages in all studied lineages, even though previous studies found that the most susceptible age for adenovirus infection was between 2–20 weeks [28,29]. However, Adel et al., 2021 [30], as in our study, found the virus in birds at 4 days of age, suggesting that in these animals, there may have been vertical transmission of the virus [12]. Another significant aspect of vertical transmission that must be considered is that adenoviruses can remain latent and not be detected for some time and can be reactivated in young birds that are immunocompromised [31,32], so we could be detecting the virus in young birds. Several experimental studies using RSS-associated enteric virus strains show that the digestive tract of birds is the organ where a high viral load is found, and the jejunum has more pathological lesions [33,34,35,36,37]. In the present study, the digestive tract of the analyzed birds presented lesions (thin walled) and the presence of gas bubbles, undigested food, and diarrhea. The jejunum was used for the detection and quantification of FAdV showing 56.6% of samples positive for the virus. The other organs of the coelomatic cavity of the analyzed birds did not present any apparent lesions and were not subjected to molecular analysis. 

In addition, it was possible to quantify the number of viral particles present in the samples studied by using a gene from a conserved region of the adenovirus genome, which helps the PCR efficiency (Table 2). We found concentrations ranging from 393 viral gene copies to 4.6 × 10^6^; these values are slightly different from those found in another study that used a different gene for quantification in different organs of birds in which the viral concentrations ranged from 1.0 × 10^7^ to 1.0 × 10^14^ [38]. However, R2 = 0.978 and slope −3.314 values and efficiency of the 100.4% curve developed in this research are quite similar to those shown in studies in which the reaction was standardized [19,21,26].

FAdVs are classified into twelve serotypes which are divided into five species (A-E) [1], and in Ecuador genotypes FAdV-6/E1 and FAdV-11/D3 have been detected in previous studies [10]. In addition, FAdV-4/C1 was also detected by RFLP [39] in our research, after sequencing analysis, genotypes FAdV-2/D2, FAdV-6/E1, FAdV-8a/E2, and FAdV-11/D3 of FAdV were identified (Figure 2). Genotypes FAdV-2/D2, FAdV-8a/E2, FAdV-8b/E3, and FAdV-11/D3 are mainly related to the production of IBH [12]. In addition, genotype FAdV-8a/E2 has also been isolated sporadically in AGE outbreaks [40]. In contrast, genotype FAdV-6/E1 was genotyped in IBH outbreaks [41] and genotype FAdV-11/D3 has also been identified in birds with RSS [11].

Previous studies indicate that in birds up to five weeks of age, layers or breeders, that presented IBH, signs such as poor growth, prostration, apathy, and ruffled feathers were also observed [1,12,39]. In the birds studied, the main signs observed were dwarfism, growth retardation, and diarrhea, affecting animals (chickens, layer and breeder hens) between 7–42 days. Likewise, in the study carried out by Adel et al., 2021 [30], they described gastrointestinal alterations and weight loss in a 33-day-old broiler, and some studies stated that if there is vertical transmission of the disease and depending on the strain that affects the population, noticeable decreases in weight gain and other adverse effects on different production parameters can be observed [12,42]. Herein, FAdVs were detected in young animals in BC (7 days old) and BH (4 days old) suggesting the possible vertical transmission of the virus, and the possible association with the enteric problems that these animals suffered. Situations where viruses cause problems in the development of animals have severe consequences for production, since the affected birds are unlikely to recover from the different signs, especially growth retardation, until they reach the slaughter stage [14]. This aspect is very important to consider, since most of the animals analyzed in this study presented dwarfism and FAdVs were detected; however, it cannot be concluded that the FAdVs genotypes found in the present study are responsible for the digestive alterations and the lack of development of the animals, making it important to carry out experimental studies using virus isolates in order to determine the possible role of FAdV in the genesis of these enteric alterations. 

Other studies suggest that in birds, erosions presented in the gizzard, both the epithelial lining of the gizzard and sections of the alimentary tract, can serve as reservoirs for FAdV and play an essential role in viral transmission, which is similar to some human adenoviruses that specifically adhere to and infect epithelial cells of the alimentary system [43]; therefore, clinical signs, such as diarrhea, were observed in most of the animals we studied. Thus, the presence of FAdV in the digestive tract of the analyzed animals shows that these samples could be considered as one of the main sources of virus dissemination.

In our country, vaccination against FAdV is used with inactivated vaccines of genotype FAdV-8a/E2 [10] and is intended for the vaccination of broiler chickens and breeders for the prevention of IBH and HHS, but the protection capacity that this vaccine generates in animals has not been studied or determined. However, studies carried out with FAdV-8a/E2 or FAdV-8b/E3 viral strains as candidates for possible vaccines indicate that the strain could be effective in protecting against infections with homologous strains and with some heterologous strains, specifically against strains of the FAdV-8a/D2, FAdV-8b/E3, and FAdV-11/D3 genotypes [1,12,44,45]. These findings could suggest that vaccination in Ecuador should protect against infections with genotypes FAdV-8a/E2 and FAdV-11/D3, and could possibly generate protection against genotypes FAdV-2/D2 and FadV-6/E1, since they are included in species D and E of FadV. For its purpose, it would be important to carry out experimental studies in order to determine if this vaccine strain generates protection against these other genotypes. 

As mentioned above, the present study shows that four FadV genotypes (FadV-2/D2, FadV-6/E1, FadV-8a/E2, and FadV-11/D3) are circulating in commercial chicken flocks in Ecuador, showing that these viral genotypes are spreading rapidly in the gastrointestinal tract of commercial birds, which could be related to the presentation of the enteric problems that the birds studied present, but in order to confirm that these FAdV genotypes could be the cause of the presentation of IBH or HHS, more studies would have to be carried out in order to test this theory. 

On the other hand, the birds analyzed in this study were not vaccinated, so it can be deduced that the viral strains that are present in commercial batches are field strains. Many studies showed that the genotypes characterized here were associated with outbreaks of IBH or HHS in chickens in several countries around the world [41,46,47,48]; however, the vast majority of reports of IBH or HHS worldwide have been associated with FAdV-4/C1 [23,49,50,51,52] and the report of a large number of outbreaks associated with this FAdV genotype has led many studies to focus on the development of vaccines based on this viral genotype [53,54,55], as well as associations with other genotypes (FAdV-8b/E3) where excellent protection against infections with both genotypes is shown [1,12,56,57,58]. However, these recombinant vaccines have not been tested with other FAdV genotypes that are included in the same group to which the vaccine strains belong, in the case of FAdV-8b/E3 belonging to species E, perhaps it could generate protection against FAdV-8a/E2 and FAdV-6/E1 strains and be an alternative in the country. Thus, the results obtained in this study show that other adenovirus genotypes beyond FAdV-8a/E2 (vaccine) are circulating in Ecuador, and it can also be seen that the virus is present in broilers, layers, and breeders. 

Additionally, the virus was detected in birds at early ages and in relatively high concentrations, which is why it is necessary to carry out more studies to help understand the dynamics of the virus in the country’s poultry population, since studies show that the use of a vaccine genotype can reduce the presentation of the same virus; however, it also increases the presentation of other genotypes [59]. Additionally, it is important to determine whether vaccines or the implementation of better biosecurity practices would help decrease viral transmission and different signs of disease.

## 5. Conclusions 

The results in this investigation indicate that several genotypes of FAdV that have been associated with IBH and HHS are circulating in commercial poultry flocks in some provinces in Ecuador, suggesting that these genotypes could be responsible for the outbreaks of IBH or HHS and could also be associated with the presentation of enteric problems (diarrhea), making it important to carry out new or enhanced vaccination plans. 

## Figures and Tables

**Table 1:** Primer sequences, gene targets, and assays used for the amplification and quantification of FAdV.

Virus	Gene Target	Primer Name	Primer Sequence	Amplicon Size (bp)	Assay	Reference
FAdV	52K and pIIIa	52K-fw	5′-ATG GCK CAG ATG GCY AAG G-3′	176	qPCR	[21]
		52K-rv	5′-AGC GCC TGG GTC AAA CCG A-3′			
		52K-F	5′-TGT ACG AYT TCG TSC ARA C-3′	773	PCR	
		52K-R	5′-TAR ATG GCG CCY TGC TC-3′			
	Hexon	Hexon A	5′-CAARTTCAGRCAGACGGT-3′	897		[17]
		Hexon B	5′-TAGTGATGMCGSGACATCAT-3′			

FAdV = Fowl adenovirus and bp = base pair.

**Table 2:** Molecular detection and quantification of FAdV in Ecuadorian chicken flocks.

Lineage	Age		Number of Analyzed Samples	Clinical Signs			Negative Samples to FAdV	Sample Origin	Positive Samples to FAdV	Average of Viral Gene Copies/mg of tissue		Sample Origin
				Dwarfism	Stunting	Diarrhea						
**Broiler**	**Days**	7	16	16/16	16/16	14/16	13	(10) EC-H, (2) EC-I, (1) EC-P	3	327.51	**a**	(1) EC-H, (1) EC-P, (1) EC-T
		12	2	2/2	2/2	2/2			2	866,152.96		(2) EC-P
		14	9	9/9	6/9	9/9	5	(3) EC-H, (1) EC-I, (1) EC-P	4	54,394.66		(2) EC-I, (2) EC-P
		21	5	5/5	4/5	5/5	3	(3) EC-P	2	3842.90		(2) EC-P
		25	3	3/3	3/3	1/3	1	(1) EC-P	2	142.89		(2) EC-P
		26	1	1/1	1/1	1/1			1	33.10		(1) EC-P
		27	1	1/1	1/1	1/1			1	DNQ		(1) EC-P
		28	4	4/4	3/4	4/4	2	(2) EC-P	2	1,199,504.49		(2) EC-P
		29	1	1/1	1/1	1/1	1	(1) EC-P				
		30	1	1/1	1/1	1/1			1	556.63		(1) EC-P
		31	1	1/1	1/1	1/1			1	DNQ		(1) EC-P
		32	1	1/1	1/1	1/1			1	14,050.52		(1) EC-P
		33	1	1/1	1/1	1/1			1	188.93		(1) EC-P
		34	1	1/1	1/1	1/1	1	(1) EC-P				
		35	14	14/14	10/14	14/14	10	(1) EC-H, (9) EC-P	4	2,999,651.62		(4) EC-P
		36	1	1/1	0/1	1/1			1	668,606.68		(1) EC-P
		42	131	120/131	120/131	131/131	43	(6) EC-H, (2) EC-I, (35) EC-P	88	270,140.35		(4) EC-I, (84) EC-P
**Breeder Hens**	**Days**	4	4	4/4	2/4	4/4	2	(2) EC-P	2	4,660,482.12	**a**	(2) EC-P
**Layer Hens**	**Weeks**	14	1	1/1	1/1	1/1			1	393.39	**b**	(1) EC-P
		30	3	3/3	2/2	3/3	1	(1) EC-P	2	883.37		(2) EC-P
		35	6	6/6	5/6	6/6	4	(4) EC-P	2	7976.76		(2) EC-P
		40	5	5/5	5/5	5/5	3	(3) EC-P	2	3,601,094.58		(2) EC-P
		45	3	3/3	2/2	3/3	1	(1) EC-P	2	37,149.28		(2) EC-T
		48	4	4/4	2/4	4/4	4	(4) EC-T				
		50	2	2/2	2/2	2/2	2	(2) EC-T				
	**Total**						96		125			

FAdV = Fowl adenovirus, () = Number of samples in each province, EC-H = Ecuador—Province of Chimborazo, EC-I = Ecuador—Province of Imbabura, EC-P = Ecuador—Province of Pichincha, and EC-T = Ecuador—Province of Tungurahua; a = significance Tukey test; b = significance Tukey HSD test.

## Data Availability

This article contains all of the data that were created or analyzed throughout the investigation.

## Supplementary Materials

The following supporting information can be downloaded at: https://www.mdpi.com/article/10.3390/vetsci10020115/s1, Table S1: Comparison of the amino acid identities of the sequences of Ecuadorian isolates of FAdV with other sequences of this virus; Table S2: Molecular Characterization of FAdV in Ecuadorian Chicken Flocks; Table S3: Comparison of the nucleotide identities of the sequences of Ecuadorian isolates of FAdV with other sequences of this virus.

## References

[1] Hess A., Swayne D., Boulaine E., Logue C., McDougald L., Nair V., Suarez D.L. (2020). Infection.

[2] Benko K., Aoki N., Arnberg A.J., Davison M., Echavarria M., Hess M.S., Jones G.L., Kajan A.E., Kajon S.K., Mittal I.I. (2022). ICTV Virus Taxonomy Profile: Adenoviridae. J. Gen. Virol..

[3] Berk A., Knipe S.E., Howley D.M., Griffin P.M., Lamb D.E., Martin R.A., Roizman M.A., Straus B. (2007). The Viruses and Their Replication.

[4] Harrach Z.L., Tarján M., Benkő M. (2019). Adenoviruses across the animal kingdom: A walk in the zoo. FEBS Lett..

[5] Fitzgerald S.D., Rautenschlein S., Mahsoub H.M., Pierson F.W., Reed W.M., Jack S.W. (2020). Adenovirus Infection.

[6] Marek A., Günes E., Schulz M., Hess M.S. (2010). Classification of fowl adenoviruses by use of phylogenetic analysis and high-resolution melting-curve analysis of the hexon L1 gene region. J. Virol. Methods.

[7] Nakamura M., Mase Y., Yamamoto K., Takizawa M., Kabeya T., Wakuda M., Matsuda T., Chikuba Y., Yamamoto T., Ohyama N. (2010). Inclusion Body Hepatitis Caused by Fowl Adenovirus in Broiler Chickens in Case Report—Inclusion Body Hepatitis Caused by Fowl Adenovirus in Broiler Chickens in Japan, 2009–2010. Avian Pathol..

[8] Mettifogo E., Nuñez L.F., Chacón J.L., Santander Parra S.H., Astolfi-Ferreira C.S., Jerez J.A., Jones R.C., Piantino Ferreira A.J. (2014). Emergence of Enteric Viruses in Production Chickens is a Concern for Avian Health. Cient. World J..

[9] Niczyporuk J.S. (2016). Phylogenetic and geographic analysis of fowl adenovirus field strains isolated from poultry in Poland. Arch. Virol..

[10] De la Torre D., Torres B.H.P., Quezada E.C.M., Ferreira A.J.P. (2018). Molecular characterization of fowl adenovirus in commercial chicken flocks in Ecuador. Granja.

[11] De la Torre L.F.N., Nuñez S.H., Santander Parra C.S., Astolfi-Ferreira A.J., Piantino F. (2018). Molecular characterization of fowl adenovirus group I in commercial broiler chickens in Brazil. Virus Dis..

[12] Schachner M., Matos B., Grafl M., Hess M.S. (2018). Fowl adenovirus-induced diseases and strategies for their control–A review on the current global situation. Avian Pathol..

[13] Kaján I., Affranio A., Tóthné B., Kecskeméti S., Benkő M. (2019). An emerging new fowl adenovirus genotype. Heliyon.

[14] Mo J. (2021). Historical investigation of fowl adenovirus outbreaks in South Korea from 2007 to 2021: A comprehensive review. Viruses.

[15] Adair J.M., Zavala L., Swayne D., Glisson J., Pearson J., Reed W., Jackwood M., Woolcock P. (2008). Adenoviruses.

[16] Toro O., González C., Escobar L., Cerda M.A., Morales C. (2001). Vertical Induction of the Inclusion Body Hepatitis/Hydropericardium Syndrome with Fowl Adenovirus and Chicken Anemia Virus. Avian Dis..

[17] Meulemans G., Boschmans M., Berg T.P.V.D., Decaesstecker M. (2001). Polymerase chain reaction combined with restriction enzyme analysis for detection and differentiation of fowl adenoviruses. Avian Pathol..

[18] Meulemans G., Couvreur B., Decaesstecker M., Boschmans M., Berg T.P.V.D. (2004). Phylogenetic analysis of fowl adenoviruses. Avian Pathol..

[19] Günes A., Marek M., Hess M.S. (2013). Species determination of fowl adenoviruses based on the 52K gene region. Avian Dis..

[20] Green J., Sambrook J. (2017). Isolation of high-molecular-weight DNA using organic solvents. Cold Spring Harb. Protoc..

[21] Günes A., Marek B., Grafl E., Berger M., Hess M.S. (2012). Real-time PCR assay for universal detection and quantitation of all five species of fowl adenoviruses (FAdV-A. to FAdV-E). J. Virol. Methods..

[22] Kumar S., Stecher G., Tamura K. (2016). MEGA7: Molecular Evolutionary Genetics Analysis Version 7.0 for Bigger Datasets. Mol. Biol. Evol..

[23] Li L., Luo L., Luo Q., Zhang T., Zhao K., Wang H., Zhang R., Lu Q., Pan Z., Shao H. (2016). Genome sequence of a fowl adenovirus serotype 4 strain lethal to chickens, isolated from China. Genome Announc..

[24] Raue M., Hess M.S. (1998). Hexon based PCRs combined with restriction enzyme analysis for rapid detection and differentiation of fowl adenoviruses and egg drop syndrome virus. J. Virol. Methods.

[25] Mase M., Nakamura K., Imada T. (2010). Characterization of Fowl adenovirus serotype 4 isolated from chickens with hydropericardium syndrome based on analysis of the short fiber protein gene. J. Vet. Diagn. Investig..

[26] Hanson M.R., Rudis M., Vasquez-Lee R.D., Montgomery R.D. (2006). A broadly applicable method to characterize large DNA viruses and adenoviruses based on the DNA polymerase gene. Virol. J..

[27] Kaján S., Sameti M., Benko M. (2011). Partial sequence of the DNA-dependent DNA polymerase gene of fowl adenoviruses: A reference panel for a general diagnostic PCR in poultry. Acta Vet. Hung..

[28] Hafez M.H. (2011). Avian adenoviruses infections with special attention to inclusion body hepatitis/hydropericardium syndrome and egg drop syndrome. Pak. Vet. J..

[29] Şahindokuyucu F., Çöven H., Kılıç Ö., Yılmaz M., Kars Ö., Yazıcıoğlu E., Ertunç Z., Yazıcı Z. (2020). First report of fowl aviadenovirus serotypes FAdV-8b and FAdV-11 associated with inclusion body hepatitis in commercial broiler and broiler-breeder flocks in Turkey. Arch. Virol..

[30] Adel A.A.E., Mohamed M., Samir N.M., Hagag A., Erfan M., Said A.E.S., Arafa W.M.M., Hassan M.E., El Zowalaty M.A., Shahien M.A. (2021). Epidemiological and molecular analysis of circulating fowl adenoviruses and emerging of serotypes 1, 3, and 8b in Egypt. Heliyon.

[31] McFerran B.M.C., Adair B. (1977). Avian adenoviruses—A review. Avian Pathol..

[32] Fadly B.J., Riegle K., Nazerian E.A., Stephens E.A. (1980). Some Observations on an Adenovirus Isolated from Specific Pathogen Free Chickens. Poult. Sci..

[33] Kang M., El-Gazzar H.S., Sellers F., Dorea S.M., Williams T., Kim S., Collett E., Mundt E. (2012). Investigation into the aetiology of runting and stunting syndrome in chickens. Avian Pathol..

[34] Kang E., Linnemann A.H., Icard V., Durairaj E., Mundt H.S., Sellers C. (2018). Chicken astrovirus as an aetiological agent of runting-stunting syndrome in broiler chickens. J. Gen. Virol..

[35] Jindal D.P., Patnayak A.F., Ziegler A., Lago S.M., Goyal S. (2009). Experimental reproduction of poult enteritis syndrome: Clinical findings, growth response, and microbiology. Poult. Sci..

[36] Zsak R.M., Cha J.M., Day A.R.M., Cha R.M. (2013). Chicken parvovirus-induced runting-stunting syndrome in young broilers. Avian Dis..

[37] Nuñez L.F.N., Santander-Parra S.H., Kyriakidis N.C., Astolfi-Ferreira C.S., Buim M.R., De La Torre D., Ferreira A.J.P. (2020). Molecular characterization and determination of relative cytokine expression in naturally infected day-old chicks with chicken astrovirus associated to white chick syndrome. Animals.

[38] Niczyporuk H., Czekaj H. (2018). A comparative pathogenicity analysis of two adenovirus strains, 1/A and 8a/E, isolated from poultry in Poland. Arch. Virol..

[39] House A., Mazaheri C., Prusas M., Hess M.S. (1998). Some strains of serotype 4 fowl adenoviruses cause inclusion body hepatitis and hydropericardium syndrome in chickens. Avian Pathol..

[40] Mase K., Nakamura K. (2014). Phylogenetic analysis of fowl adenoviruses isolated from chickens with gizzard erosion in Japan. J. Vet. Med. Sci..

[41] Niczyporuk W., Kozdrun H., Czekaj K., Piekarska N., Stys F. (2021). Characterisation of adenovirus strains represented species B and E isolated from broiler chicken flocks in eastern Poland. Heliyon.

[42] Grafl F., Aigner D., Liebhart A., Marek I., Prokofieva J., Bachmeier M., Hess M.S. (2012). Vertical transmission and clinical signs in broiler breeders and broilers experiencing adenoviral gizzard erosion. Avian Pathol..

[43] Steer J.R., Sandy D., O’Rourke P.C., Scott G.F., Browning A.H., Noormohammadi A. (2015). Chronological analysis of gross and histological lesions induced by field strains of fowl adenovirus serotypes 1, 8b and 11 in one-day-old chickens. Avian Pathol..

[44] Gupta S., Popowich D., Ojkic S., Kurukulasuriya B., Chow-Lockerbie T., Gunawardana K., Goonewardene R., Karunarathna L.E., Ayalew K.A., Ahmed S.K. (2018). Inactivated and live bivalent fowl adenovirus (FAdV8b + FAdV11) breeder vaccines provide broad-spectrum protection in chicks against inclusion body hepatitis (IBH). Vaccine.

[45] Steer-Cope J.R., Sandy D., O’Rourke P.C., Scott G.F., Browning A.H., Noormohammadi A. (2019). Vaccination with FAdV-8a induces protection against inclusion body hepatitis caused by homologous and heterologous strains. Avian Pathol..

[46] Chitradevi K., Sukumar P., Suresh G.A., Balasubramaniam D., Kannan D. (2021). Molecular typing and pathogenicity assessment of fowl adenovirus associated with inclusion body hepatitis in chicken from India. Trop. Anim. Health Prod..

[47] Liu X., Shi L., Lv K., Wang Z., Yang Y., Li H., Chen H. (2021). Characterization of Co-infection With Fowl Adenovirus Serotype 4 and 8a. Front. Microbiol..

[48] Xie J., Zhang M., Sun Q., Zeng Y., Huang J., Dong L., Li S., Huang M., Liao M. (2022). The first complete genome sequence and pathogenicity characterization of fowl adenovirus serotype 2 with inclusion body hepatitis and hydropericardium in China. Front. Vet. Sci..

[49] Li L., Wang J., Chen P., Zhang S., Sun J., Yuan W. (2018). Pathogenicity and molecular characterization of a fowl adenovirus 4 isolated from chicken associated with IBH and HPS in China. BMC Vet. Res..

[50] Ishag H.Z.A., Terab A.M.A., El Tigani-Asil E.T.A., Bensalah O.K., Khalil N.A.H., Khalafalla A.I., Al Hammadi Z.M.A.H., Shah A.A.M., Al Muhairi S.S.M., Al Hammadi A.A.M. (2022). Pathology and Molecular Epidemiology of Fowl Adenovirus Serotype 4 Outbreaks in Broiler Chicken in Abu Dhabi Emirate, UAE. Vet. Sci..

[51] Changjing L., Haiying W., Dongdong W., Jingjing W., Youming W., Shouchun L., Jida L., Ping W., Jianlin X., Shouzhen C. (2016). Characterization of fowl adenoviruses isolated between 2007 and 2014 in China. Vet. Microbiol..

[52] Guan Y., Tian X., Han X., Yang H., Wang H. (2018). Complete genome sequence and pathogenicity of fowl adenovirus serotype 4 involved in hydropericardium syndrome in Southwest China. Microb. Pathog..

[53] Meng X., Yuan J., Yu Y., Zhang W., Ai Y., Wang Y. (2019). Identification, pathogenicity of novel fowl adenovirus serotype 4 SDJN0105 in shandong, china and immunoprotective evaluation of the newly developed inactivated oil-emulsion FAdV-4 vaccine. Viruses.

[54] Pei J.C., Corredor B.D., Griffin P.J., Krell É., Nagy E. (2018). Fowl adenovirus 4 (FAdV-4)-based infectious clone for vaccine vector development and viral gene function studies. Viruses.

[55] Xie W., Wang Q., Kan Y., Mu W., Zhang J., Chen L., Li H., Fu T., Li Z., Wan Z. (2022). FAdV-4 without Fiber-2 Is a Highly Attenuated and Protective Vaccine Candidate. Microbiol. Spectr..

[56] De Luca A., Schachner T., Mitra S., Heidl D., Liebhart M., Hess M.S. (2020). Fowl adenovirus (FAdV) fiber-based vaccine against inclusion body hepatitis (IBH) provides type-specific protection guided by humoral immunity and regulation of B and T cell response. Vet. Res..

[57] De Luca C., Schachner A., Heidl S., Hess M.S. (2022). Vaccination with a fowl adenovirus chimeric fiber protein (crecFib-4/11) simultaneously protects chickens against hepatitis-hydropericardium syndrome (HHS) and inclusion body hepatitis (IBH). Vaccine.

[58] Lu Q., Xie W., Zhang J., Zhang W., Wang M., Lian Z., Zhao D., Ren S., Xie Y., Lin T. (2022). A Novel Recombinant FAdV-4 Virus with Fiber of FAdV-8b Provides Efficient Protection against Both FAdV-4 and FAdV-8b. Viruses.

[59] Lai K., Min H.T.L., Lai J., Mo J. (2021). Epidemiology of fowl adenovirus (FAdV) infections in South Korean chickens during 2013–2019 following introduction of FAdV-4 vaccines. Avian Pathol..

