# Supplementary material for: Detection, Quantification and Molecular Characterization of Fowl Adenoviruses Circulating in Ecuadorian Chicken Flocks during 2019–2021
Source: Vet Sci. 2023 Feb 3;10(2):115. doi: 10.3390/vetsci10020115 (PMC9963715; doi:10.3390/vetsci10020115)
Supplement: Supplementary file 1 [file vetsci-10-00115-s001.zip › Table S1.pdf]

Table S1. Comparison of the amino acid identities of the sequences of Ecuadorian isolates of FAdV with other sequences of this virus.

	1. YP - ...	2. ADQ...	3. AUO...	5. ANV2...	6. ANV2...	7. AYU5...	8. QNI2...	9. CCE3...	10. QJZ...	11. NP ...	12. AAC...	13. APP...	14. YP ...	15. AGL...	16. QCC...	17. UDL...	18. ASU...	19. ANJ...	20. YP ...	21. YP ...	22. ADE...	23. AOC...	25. AQX...	26. QIZ...	27. ANJ...	28. ANJ...	30. UDL...	31. UDL...
1. YP - NC_015323 C...		100%	95.5%	95.5%	95.5%	95.5%	95.5%	100%	93.9%	60.8%	60.8%	61.3%	53.9%	53.9%	51.9%	51.5%	51.5%	51.5%	51.5%	52.4%	52.4%	51.9%	52.4%	51.9%	52.9%	52.9%	54.9%	54.9%
2. ADQ39061.1 | - GU18...	100%		95.5%	95.5%	95.5%	95.5%	95.5%	100%	93.9%	60.8%	60.8%	61.3%	53.9%	53.9%	51.9%	51.5%	51.5%	51.5%	51.5%	52.4%	52.4%	51.9%	52.4%	51.9%	52.9%	52.9%	54.9%	54.9%
3. AUO29782.1 | KY636...	95.5%	95.5%		100%	100%	100%	100%	95.5%	92.4%	61.8%	61.8%	62.3%	53.4%	53.4%	52.4%	52.9%	52.9%	52.9%	52.9%	52.4%	52.4%	52.4%	52.4%	51.9%	52.9%	53.4%	54.9%	54.9%
5. ANV21438.1 | KU558...	95.5%	95.5%	100%		100%	100%	100%	95.5%	92.4%	61.8%	61.8%	62.3%	53.4%	53.4%	52.4%	52.9%	52.9%	52.9%	52.9%	52.4%	52.4%	52.4%	52.4%	51.9%	52.9%	53.4%	54.9%	54.9%
6. ANV21566.1 | KU569...	95.5%	95.5%	100%	100%		100%	100%	95.5%	92.4%	61.8%	61.8%	62.3%	53.4%	53.4%	52.4%	52.9%	52.9%	52.9%	52.9%	52.4%	52.4%	52.4%	52.4%	51.9%	52.9%	53.4%	54.9%	54.9%
7. AYU58961.1 | MH454...	95.5%	95.5%	100%	100%	100%		100%	95.5%	92.4%	61.8%	61.8%	62.3%	53.4%	53.4%	52.4%	52.9%	52.9%	52.9%	52.9%	52.4%	52.4%	52.4%	52.4%	51.9%	52.9%	53.4%	54.9%	54.9%
8. QNI22031.1 | MN781...	95.5%	95.5%	100%	100%	100%	100%		95.5%	92.4%	61.8%	61.8%	62.3%	53.4%	53.4%	52.4%	52.9%	52.9%	52.9%	52.9%	52.4%	52.4%	52.4%	52.4%	51.9%	52.9%	53.4%	54.9%	54.9%
9. CCE39367.1 | HE608...	100%	100%	95.5%	95.5%	95.5%	95.5%	95.5%		93.9%	60.8%	60.8%	61.3%	53.9%	53.9%	51.9%	51.5%	51.5%	51.5%	51.5%	52.4%	52.4%	51.9%	52.4%	51.9%	52.9%	52.9%	54.9%	54.9%
10. QJZ28073.1 | MN54...	93.9%	93.9%	92.4%	92.4%	92.4%	92.4%	92.4%	93.9%		61.8%	61.8%	62.3%	53.9%	53.9%	53.4%	52.4%	52.4%	52.4%	52.4%	52.4%	52.4%	51.9%	52.4%	51.0%	52.9%	51.9%	55.3%	55.3%
11. NP NC_001720.1 - A...	60.8%	60.8%	61.8%	61.8%	61.8%	61.8%	61.8%	60.8%	61.8%		100%	99.5%	63.1%	63.1%	61.2%	59.8%	59.8%	59.8%	59.8%	59.8%	59.8%	59.8%	59.8%	59.8%	60.3%	60.8%	60.3%	60.3%
12. AAC54912.1 | U469...	60.8%	60.8%	61.8%	61.8%	61.8%	61.8%	61.8%	60.8%	61.8%	100%		99.5%	63.1%	63.1%	61.2%	59.8%	59.8%	59.8%	59.8%	59.8%	59.8%	59.8%	59.8%	59.8%	60.3%	60.8%	60.3%	60.3%
13. APP94097.1 | KX247...	61.3%	61.3%	62.3%	62.3%	62.3%	62.3%	62.3%	61.3%	62.3%	99.5%	99.5%		62.6%	62.6%	60.7%	60.3%	60.3%	60.3%	60.3%	60.3%	60.3%	60.3%	60.3%	60.3%	60.8%	61.3%	60.8%	60.8%
14. YP NC_021221.1 NO...	53.9%	53.9%	53.4%	53.4%	53.4%	53.4%	53.4%	53.9%	53.9%	63.1%	63.1%	62.6%		100%	91.7%	68.9%	68.9%	68.9%	68.9%	67.0%	67.0%	67.0%	67.5%	67.0%	67.5%	68.4%	68.4%	68.4%
15. AGL34687.1 | KC493...	53.9%	53.9%	53.4%	53.4%	53.4%	53.4%	53.4%	53.9%	53.9%	63.1%	63.1%	62.6%	100%		91.7%	68.9%	68.9%	68.9%	68.9%	67.0%	67.0%	67.0%	67.5%	67.0%	67.5%	68.4%	68.4%	68.4%
16. QCC26479.1 | MG95...	51.9%	51.9%	52.4%	52.4%	52.4%	52.4%	52.4%	51.9%	53.4%	61.2%	61.2%	60.7%	91.7%	91.7%		70.4%	70.4%	70.4%	70.4%	67.5%	67.5%	67.5%	68.0%	67.5%	68.0%	69.9%	68.4%	68.4%
17. UDLA 71 ECUAD...	51.5%	51.5%	52.9%	52.9%	52.9%	52.9%	52.9%	51.5%	52.4%	59.8%	59.8%	60.3%	68.9%	68.9%	70.4%		100%	100%	100%	84.8%	84.8%	85.3%	85.3%	85.3%	85.8%	91.2%	87.3%	87.3%
18. ASU91620.1 |MF161...	51.5%	51.5%	52.9%	52.9%	52.9%	52.9%	52.9%	51.5%	52.4%	59.8%	59.8%	60.3%	68.9%	68.9%	70.4%	100%		100%	100%	84.8%	84.8%	85.3%	85.3%	85.3%	85.8%	91.2%	87.3%	87.3%
19. ANJ02455.1 | KT862...	51.5%	51.5%	52.9%	52.9%	52.9%	52.9%	52.9%	51.5%	52.4%	59.8%	59.8%	60.3%	68.9%	68.9%	70.4%	100%	100%		100%	84.8%	84.8%	85.3%	85.3%	85.3%	85.8%	91.2%	87.3%	87.3%
20. YP NC_038332.1 JAP...	51.5%	51.5%	52.9%	52.9%	52.9%	52.9%	52.9%	51.5%	52.4%	59.8%	59.8%	60.3%	68.9%	68.9%	70.4%	100%	100%	100%		84.8%	84.8%	85.3%	85.3%	85.3%	85.8%	91.2%	87.3%	87.3%
21. YP NC_014969.1 CA...	52.4%	52.4%	52.4%	52.4%	52.4%	52.4%	52.4%	52.4%	52.4%	59.8%	59.8%	60.3%	67.0%	67.0%	67.5%	84.8%	84.8%	84.8%	84.8%		100%	96.6%	99.5%	95.6%	99.0%	89.2%	84.3%	84.3%
22. ADE58399.1 |GU73...	52.4%	52.4%	52.4%	52.4%	52.4%	52.4%	52.4%	52.4%	52.4%	59.8%	59.8%	60.3%	67.0%	67.0%	67.5%	84.8%	84.8%	84.8%	84.8%	100%		96.6%	99.5%	95.6%	99.0%	89.2%	84.3%	84.3%
23. AOC84071.1 | KX25...	51.9%	51.9%	52.4%	52.4%	52.4%	52.4%	52.4%	51.9%	51.9%	59.8%	59.8%	60.3%	67.0%	67.0%	67.5%	85.3%	85.3%	85.3%	85.3%	96.6%	96.6%		97.1%	97.1%	96.6%	89.2%	83.8%	83.8%
25. AQX17370.1 | KY229...	52.4%	52.4%	52.4%	52.4%	52.4%	52.4%	52.4%	52.4%	52.4%	59.8%	59.8%	60.3%	67.5%	67.5%	68.0%	85.3%	85.3%	85.3%	85.3%	99.5%	99.5%	97.1%		96.1%	99.5%	89.7%	84.8%	84.8%
26. QIZ86862.1 | MT104...	51.9%	51.9%	51.9%	51.9%	51.9%	51.9%	51.9%	51.9%	51.0%	59.8%	59.8%	60.3%	67.0%	67.0%	67.5%	85.3%	85.3%	85.3%	85.3%	95.6%	95.6%	97.1%	96.1%		95.6%	90.7%	83.3%	83.3%
27. ANJ02566.1 | KT862...	52.9%	52.9%	52.9%	52.9%	52.9%	52.9%	52.9%	52.9%	52.9%	60.3%	60.3%	60.8%	67.5%	67.5%	68.0%	85.8%	85.8%	85.8%	85.8%	99.0%	99.0%	96.6%	99.5%	95.6%		90.2%	85.3%	85.3%
28. ANJ02492.1 |KT8628...	52.9%	52.9%	53.4%	53.4%	53.4%	53.4%	53.4%	52.9%	51.9%	60.8%	60.8%	61.3%	68.4%	68.4%	69.9%	91.2%	91.2%	91.2%	91.2%	89.2%	89.2%	89.2%	89.7%	90.7%	90.2%		85.3%	85.3%
30. UDLA 4 ECUADOR	54.9%	54.9%	54.9%	54.9%	54.9%	54.9%	54.9%	54.9%	55.3%	60.3%	60.3%	60.8%	68.4%	68.4%	68.4%	87.3%	87.3%	87.3%	87.3%	84.3%	84.3%	83.8%	84.8%	83.3%	85.3%	85.3%		100%
31. UDLA 17 ECUADOR	54.9%	54.9%	54.9%	54.9%	54.9%	54.9%	54.9%	54.9%	55.3%	60.3%	60.3%	60.8%	68.4%	68.4%	68.4%	87.3%	87.3%	87.3%	87.3%	84.3%	84.3%	83.8%	84.8%	83.3%	85.3%	85.3%	100%	
34. UDLA 310-2 ECUAD...	54.9%	54.9%	54.9%	54.9%	54.9%	54.9%	54.9%	54.9%	55.3%	60.3%	60.3%	60.8%	68.4%	68.4%	68.4%	87.3%	87.3%	87.3%	87.3%	84.3%	84.3%	83.8%	84.8%	83.3%	85.3%	85.3%	100%	100%
35. UDLA 292-2 ECUA...	54.9%	54.9%	54.9%	54.9%	54.9%	54.9%	54.9%	54.9%	55.3%	60.3%	60.3%	60.8%	68.4%	68.4%	68.4%	87.3%	87.3%	87.3%	87.3%	84.3%	84.3%	83.8%	84.8%	83.3%	85.3%	85.3%	100%	100%
36. UDLA 282 ECUADOR	54.9%	54.9%	54.9%	54.9%	54.9%	54.9%	54.9%	54.9%	55.3%	60.3%	60.3%	60.8%	68.4%	68.4%	68.4%	87.3%	87.3%	87.3%	87.3%	84.3%	84.3%	83.8%	84.8%	83.3%	85.3%	85.3%	100%	100%
37. UDLA 316 ECUADOR	54.9%	54.9%	54.9%	54.9%	54.9%	54.9%	54.9%	54.9%	55.3%	60.3%	60.3%	60.8%	68.4%	68.4%	68.4%	87.3%	87.3%	87.3%	87.3%	84.3%	84.3%	83.8%	84.8%	83.3%	85.3%	85.3%	100%	100%
38. UDLA 310 ECUADOR	54.9%	54.9%	54.9%	54.9%	54.9%	54.9%	54.9%	54.9%	55.3%	60.3%	60.3%	60.8%	68.4%	68.4%	68.4%	87.3%	87.3%	87.3%	87.3%	84.3%	84.3%	83.8%	84.8%	83.3%	85.3%	85.3%	100%	100%
39. UDLA 291 ECUADOR	54.9%	54.9%	54.9%	54.9%	54.9%	54.9%	54.9%	54.9%	55.3%	60.3%	60.3%	60.8%	68.4%	68.4%	68.4%	87.3%	87.3%	87.3%	87.3%	84.3%	84.3%	83.8%	84.8%	83.3%	85.3%	85.3%	100%	100%
40. UDLA 292 ECUADOR	54.9%	54.9%	54.9%	54.9%	54.9%	54.9%	54.9%	54.9%	55.3%	60.3%	60.3%	60.8%	68.4%	68.4%	68.4%	87.3%	87.3%	87.3%	87.3%	84.3%	84.3%	83.8%	84.8%	83.3%	85.3%	85.3%	100%	100%
41. UDLA 303 ECUADOR	54.9%	54.9%	54.9%	54.9%	54.9%	54.9%	54.9%	54.9%	55.3%	60.3%	60.3%	60.8%	68.4%	68.4%	68.4%	87.3%	87.3%	87.3%	87.3%	84.3%	84.3%	83.8%	84.8%	83.3%	85.3%	85.3%	100%	100%
42. QKE53464.1 | MN45...	55.3%	55.3%	54.9%	54.9%	54.9%	54.9%	54.9%	55.3%	55.3%	60.3%	60.3%	60.8%	68.4%	68.4%	68.4%	87.3%	87.3%	87.3%	87.3%	84.8%	84.8%	84.3%	85.3%	83.8%	85.8%	85.8%	99.5%	99.5%
43. AQX17379.1 | KY229...	55.3%	55.3%	54.9%	54.9%	54.9%	54.9%	54.9%	55.3%	55.3%	60.3%	60.3%	60.8%	68.4%	68.4%	68.4%	87.3%	87.3%	87.3%	87.3%	84.8%	84.8%	84.3%	85.3%	83.8%	85.8%	85.8%	99.5%	99.5%
44. ABD83864.1 | DQ32...	54.9%	54.9%	54.9%	54.9%	54.9%	54.9%	54.9%	54.9%	55.3%	60.3%	60.3%	60.8%	68.4%	68.4%	68.4%	87.3%	87.3%	87.3%	87.3%	84.3%	84.3%	83.8%	84.8%	83.3%	85.3%	85.3%	100%	100%
46. NP NC_000899.1 PO...	53.2%	53.2%	53.7%	53.7%	53.7%	53.7%	53.7%	53.2%	54.6%	61.3%	61.3%	61.8%	67.5%	67.5%	69.4%	78.4%	78.4%	78.4%	78.4%	75.5%	75.5%	76.0%	76.0%	75.5%	76.0%	76.5%	77.0%	77.0%
47. AAD50344.2 |AF083...	53.2%	53.2%	53.7%	53.7%	53.7%	53.7%	53.7%	53.2%	54.6%	61.3%	61.3%	61.8%	67.5%	67.5%	69.4%	78.4%	78.4%	78.4%	78.4%	75.5%	75.5%	76.0%	76.0%	75.5%	76.0%	76.5%	77.0%	77.0%
48. ANJ02418.1 | KT862...	53.7%	53.7%	54.1%	54.1%	54.1%	54.1%	54.1%	53.7%	54.1%	61.8%	61.8%	62.3%	68.4%	68.4%	68.9%	79.9%	79.9%	79.9%	79.9%	76.5%	76.5%	77.5%	77.0%	77.0%	77.0%	77.9%	78.4%	78.4%
49. UDLA 2 ECUADOR	53.2%	53.2%	53.7%	53.7%	53.7%	53.7%	53.7%	53.2%	51.7%	59.3%	59.3%	59.8%	68.4%	68.4%	67.5%	77.9%	77.9%	77.9%	77.9%	75.5%	75.5%	76.0%	76.0%	75.5%	75.5%	77.0%	78.9%	78.9%
50. UDLA 3 ECUADOR	53.2%	53.2%	53.7%	53.7%	53.7%	53.7%	53.7%	53.2%	51.7%	59.3%	59.3%	59.8%	68.4%	68.4%	67.5%	77.9%	77.9%	77.9%	77.9%	75.5%	75.5%	76.0%	76.0%	75.5%	75.5%	77.0%	78.9%	78.9%
51. UDLA 72 ECUADOR	53.2%	53.2%	53.7%	53.7%	53.7%	53.7%	53.7%	53.2%	51.7%	59.3%	59.3%	59.8%	68.4%	68.4%	67.5%	77.9%	77.9%	77.9%	77.9%	75.5%	75.5%	76.0%	76.0%	75.5%	75.5%	77.0%	78.9%	78.9%
52. UDLA 76 ECUADOR	52.7%	52.7%	53.2%	53.2%	53.2%	53.2%	53.2%	52.7%	51.2%	57.8%	57.8%	58.3%	67.0%	67.0%	67.0%	77.5%	77.5%	77.5%	77.5%	75.0%	75.0%	75.5%	75.5%	75.0%	75.0%	76.5%	78.4%	78.4%
53. UDLA 78 ECUADOR	52.7%	52.7%	53.2%	53.2%	53.2%	53.2%	53.2%	52.7%	51.2%	57.8%	57.8%	58.3%	67.0%	67.0%	67.0%	77.5%	77.5%	77.5%	77.5%	75.0%	75.0%	75.5%	75.5%	75.0%	75.0%	76.5%	78.4%	78.4%
54. UDLA 80 ECUADOR	53.2%	53.2%	53.7%	53.7%	53.7%	53.7%	53.7%	53.2%	51.7%	59.3%	59.3%	59.8%	68.4%	68.4%	67.5%	77.9%	77.9%	77.9%	77.9%	75.5%	75.5%	76.0%	76.0%	75.5%	75.5%	77.0%	78.9%	78.9%
55. UDLA 226 ECUADOR	53.2%	53.2%	53.7%	53.7%	53.7%	53.7%	53.7%	53.2%	51.7%	59.3%	59.3%	59.8%	68.4%	68.4%	67.5%	77.9%	77.9%	77.9%	77.9%	75.5%	75.5%	76.0%	76.0%	75.5%	75.5%	77.0%	78.9%	78.9%
56. UDLA 222 ECUADOR	53.2%	53.2%	53.7%	53.7%	53.7%	53.7%	53.7%	53.2%	51.7%	59.3%	59.3%	59.8%	68.4%	68.4%	67.5%	77.9%	77.9%	77.9%	77.9%	75.5%	75.5%	76.0%	76.0%	75.5%	75.5%	77.0%	78.9%	78.9%
57. UDLA 213 ECUADOR	53.2%	53.2%	53.7%	53.7%	53.7%	53.7%	53.7%	53.2%	51.7%	59.3%	59.3%	59.8%	68.4%	68.4%	67.5%	77.9%	77.9%	77.9%	77.9%	75.5%	75.5%	76.0%	76.0%	75.5%	75.5%	77.0%	78.9%	78.9%
58. UDLA 210 ECUADOR	53.2%	53.2%	53.7%	53.7%	53.7%	53.7%	53.7%	53.2%	51.7%	59.3%	59.3%	59.8%	68.4%	68.4%	67.5%	77.9%	77.9%	77.9%	77.9%	75.5%	75.5%	76.0%	76.0%	75.5%	75.5%	77.0%	78.9%	78.9%
59. UDLA 85 ECUADOR	53.2%	53.2%	53.7%	53.7%	53.7%	53.7%	53.7%	53.2%	51.7%	59.3%	59.3%	59.8%	68.4%	68.4%	67.5%	77.9%	77.9%	77.9%	77.9%	75.5%	75.5%	76.0%	76.0%	75.5%	75.5%	77.0%	78.9%	78.9%
60. UDLA 250 ECUADOR	53.2%	53.2%	53.7%	53.7%	53.7%	53.7%	53.7%	53.2%	51.7%	59.3%	59.3%	59.8%	68.4%	68.4%	67.5%	77.9%	77.9%	77.9%	77.9%	75.5%	75.5%	76.0%	76.0%	75.5%	75.5%	77.0%	78.9%	78.9%
61. UDLA 86-2 ECUAD...	53.2%	53.2%	53.7%	53.7%	53.7%	53.7%	53.7%	53.2%	51.7%	59.3%	59.3%	59.8%	68.4%	68.4%	67.5%	77.9%	77.9%	77.9%	77.9%	75.5%	75.5%	76.0%	76.0%	75.5%	75.5%	77.0%	78.9%	78.9%
62. UDLA 88 ECUADOR	53.2%	53.2%	53.7%	53.7%	53.7%	53.7%	53.7%	53.2%	51.7%	59.3%	59.3%	59.8%	68.4%	68.4%	67.5%	77.9%	77.9%	77.9%	77.9%	75.5%	75.5%	76.0%	76.0%	75.5%	75.5%	77.0%	78.9%	78.9%
63. UDLA 207 ECUADOR	53.2%	53.2%	53.7%	53.7%	53.7%	53.7%	53.7%	53.2%	51.7%	59.3%	59.3%	59.8%	68.4%	68.4%	67.5%	77.9%	77.9%	77.9%	77.9%	75.5%	75.5%	76.0%	76.0%	75.5%	75.5%	77.0%	78.9%	78.9%
64. UDLA 76F ECUADOR	53.2%	53.2%	53.7%	53.7%	53.7%	53.7%	53.7%	53.2%	51.7%	59.3%	59.3%	59.8%	68.4%	68.4%	67.5%	77.9%	77.9%	77.9%	77.9%	75.5%	75.5%	76.0%	76.0%	75.5%	75.5%	77.0%	78.9%	78.9%
65. UDLA 276 ECUADOR	53.2%	53.2%	53.7%	53.7%	53.7%	53.7%	53.7%	53.2%	51.7%	59.3%	59.3%	59.8%	68.4%	68.4%	67.5%	77.9%	77.9%	77.9%	77.9%	75.5%	75.5%	76.0%	76.0%	75.5%	75.5%	77.0%	78.9%	78.9%
66. UDLA 268 ECUADOR	53.2%	53.2%	53.7%	53.7%	53.7%	53.7%	53.7%	53.2%	51.7%	59.3%	59.3%	59.8%	68.4%	68.4%	67.5%	77.9%	77.9%	77.9%	77.9%	75.5%	75.5%	76.0%	76.0%	75.5%	75.5%	77.0%	78.9%	78.9%
67. UDLA 257 ECUADOR	53.2%	53.2%	53.7%	53.7%	53.7%	53.7%	53.7%	53.2%	51.7%	59.3%	59.3%	59.8%	68.4%	68.4%	67.5%	77.9%	77.9%	77.9%	77.9%	75.5%	75.5%	76.0%	76.0%	75.5%	75.5%	77.0%	78.9%	78.9%
68. UDLA 247 ECUADOR	53.2%	53.2%	53.7%	53.7%	53.7%	53.7%	53.7%	53.2%	51.7%	59.3%	59.3%	59.8%	68.4%	68.4%	67.5%	77.9%	77.9%	77.9%	77.9%	75.5%	75.5%	76.0%	76.0%	75.5%	75.5%	77.0%	78.9%	78.9%
69. 267 ECUADOR	53.2%	53.2%	53.7%	53.7%	53.7%	53.7%	53.7%	53.2%	51.7%	59.3%	59.3%	59.8%	68.4%	68.4%	67.5%	77.9%	77.9%	77.9%	77.9%	75.5%	75.5%	76.0%	76.0%	75.5%	75.5%	77.0%	78.9%	78.9%
70. UDLA 280 ECUADOR	53.2%	53.2%	53.7%	53.7%	53.7%	53.7%	53.7%	53.2%	51.7%	59.3%	59.3%	59.8%	68.4%	68.4%	67.5%	77.9%	77.9%	77.9%	77.9%	75.5%	75.5%	76.0%	76.0%	75.5%	75.5%	77.0%	78.9%	78.9%
71. UDLA 299 ECUADOR	53.2%	53.2%	53.7%	53.7%	53.7%	53.7%	53.7%	53.2%	51.7%	59.3%	59.3%	59.8%	68.4%	68.4%	67.5%	77.9%	77.9%	77.9%	77.9%	75.5%	75.5%	76.0%	76.0%	75.5%	75.5%	77.0%	78.9%	78.9%
72. UDLA 86 ECUADOR	53.2%	53.2%	53.7%	53.7%	53.7%	53.7%	53.7%	53.2%	51.7%	59.3%	59.3%	59.8%	68.4%	68.4%	67.5%	77.9%	77.9%	77.9%	77.9%	75.5%	75.5%	76.0%	76.0%	75.5%	75.5%	77.0%	78.9%	78.9%
73. UDLA 203 ECUADOR	53.2%	53.2%	53.7%	53.7%	53.7%	53.7%	53.7%	53.2%	51.7%	59.3%	59.3%	59.8%	68.4%	68.4%	67.5%	77.9%	77.9%	77.9%	77.9%	75.5%	75.5%	76.0%	76.0%	75.5%	75.5%	77.0%	78.9%	78.9%
74. ABD83863.1 | DQ32...	53.2%	53.2%	53.7%	53.7%	53.7%	53.7%	53.7%	53.2%	51.7%	58.8%	58.8%	59.3%	68.0%	68.0%	68.0%	78.4%	78.4%	78.4%	78.4%	76.0%	76.0%	76.5%	76.5%	76.0%	76.0%	77.5%	79.4%	79.4%
75. AOS51012.1 |KU746...	53.2%	53.2%	53.7%	53.7%	53.7%	53.7%	53.7%	53.2%	51.7%	58.8%	58.8%	59.3%	68.0%	68.0%	68.0%	78.4%	78.4%	78.4%	78.4%	76.0%	76.0%	76.5%	76.5%	76.0%	76.0%	77.5%	79.4%	79.4%
76. AKR76197.1 |KP231...	53.2%	53.2%	53.7%	53.7%	53.7%	53.7%	53.7%	53.2%	51.7%	58.8%	58.8%	59.3%	68.0%	68.0%	68.0%	78.4%	78.4%	78.4%	78.4%	76.0%	76.0%	76.5%	76.5%	76.0%	76.0%	77.5%	79.4%	79.4%
77. ASU91621.1 | MF16...	53.2%	53.2%	53.7%	53.7%	53.7%	53.7%	53.7%	53.2%	51.7%	58.8%	58.8%	59.3%	68.0%	68.0%	68.0%	78.4%	78.4%	78.4%	78.4%	76.0%	76.0%	76.5%	76.5%	76.0%	76.0%	77.5%	79.4%	79.4%
78. QIM09460.1 |MN42...	53.2%	53.2%	53.7%	53.7%	53.7%	53.7%	53.7%	53.2%	51.7%	58.8%	58.8%	59.3%	68.0%	68.0%	68.0%	78.4%	78.4%	78.4%	78.4%	76.0%	76.0%	76.5%	76.5%	76.0%	76.0%	77.5%	79.4%	79.4%
79. QFR45466.1 |MK757...	53.2%	53.2%	53.7%	53.7%	53.7%	53.7%	53.7%	53.2%	51.7%	58.8%	58.8%	59.3%	68.0%	68.0%	68.0%	78.4%	78.4%	78.4%	78.4%	76.0%	76.0%	76.5%	76.5%	76.0%	76.0%	77.5%	79.4%	79.4%
81. UDLA 50 ECUADOR	53.2%	53.2%	52.7%	52.7%	52.7%	52.7%	52.7%	53.2%	53.2%	58.3%	58.3%	58.8%	68.0%	68.0%	69.9%	79.4%	79.4%	79.4%	79.4%	76.5%	76.5%	76.5%	77.0%	76.5%	76.5%	77.9%	79.9%	79.9%
82. AQX17377.1 | MT10...	53.2%	53.2%	53.7%	53.7%	53.7%	53.7%	53.7%	53.2%	51.7%	58.8%	58.8%	59.3%	68.0%	68.0%	68.0%	78.4%	78.4%	78.4%	78.4%	76.0%	76.0%	76.5%	76.5%	76.0%	76.0%	77.5%	79.4%	79.4%
83. UDLA 337 ECUADOR	53.2%	53.2%	52.7%	52.7%	52.7%	52.7%	52.7%	53.2%	53.2%	58.3%	58.3%	58.8%	68.0%	68.0%	69.9%	79.4%	79.4%	79.4%	79.4%	76.5%	76.5%	76.5%	77.0%	76.5%	76.5%	77.9%	79.9%	79.9%
84. UDLA 64 ECUADOR	53.2%	53.2%	52.7%	52.7%	52.7%	52.7%	52.7%	53.2%	53.2%	58.3%	58.3%	58.8%	68.0%	68.0%	69.9%	79.4%	79.4%	79.4%	79.4%	76.5%	76.5%	76.5%	77.0%	76.5%	76.5%	77.9%	79.9%	79.9%
85. UDLA 56 ECUADOR	53.2%	53.2%	52.7%	52.7%	52.7%	52.7%	52.7%	53.2%	53.2%	58.3%	58.3%	58.8%	68.0%	68.0%	69.9%	79.4%	79.4%	79.4%	79.4%	76.5%	76.5%	76.5%	77.0%	76.5%	76.5%	77.9%	79.9%	79.9%
86. UDLA 44 ECUADOR	53.2%	53.2%	52.7%	52.7%	52.7%	52.7%	52.7%	53.2%	53.2%	58.3%	58.3%	58.8%	68.0%	68.0%	69.9%	79.4%	79.4%	79.4%	79.4%	76.5%	76.5%	76.5%	77.0%	76.5%	76.5%	77.9%	79.9%	79.9%
87. ANJ02344.1 | KT862...	53.2%	53.2%	52.7%	52.7%	52.7%	52.7%	52.7%	53.2%	53.2%	58.3%	58.3%	58.8%	68.0%	68.0%	69.9%	79.4%	79.4%	79.4%	79.4%	76.5%	76.5%	76.5%	77.0%	76.5%	76.5%	77.9%	79.9%	79.9%
89. NP NC_001813.1 D...	32.4%	32.4%	32.9%	32.9%	32.9%	32.9%	32.9%	32.4%	32.4%	31.1%	31.1%	31.1%	31.6%	31.6%	30.2%	31.9%	31.9%	31.9%	31.9%	33.8%	33.8%	33.3%	33.8%	32.4%	33.8%	32.1%	32.9%	32.9%

	34. UDL...	35. UDL...	36. UDL...	37. UDL...	38. UDL...	39. UDL...	40. UDL...	41. UDL...	42. QKE...	43. AQX...	44. ABD...	46. NP ...	47. AAD...	48. ANJ...	49. UDL...	50. UDL...	51. UDL...	52. UDL...	53. UDL...	54. UDL...	55. UDL...	56. UDL...	57. UDL...	58. UDL...	59. UDL...	60. UDL...	61. UDL...	62. UDL...	63. UDL...
1. YP - NC_015323 C...	54.9%	54.9%	54.9%	54.9%	54.9%	54.9%	54.9%	54.9%	55.3%	55.3%	54.9%	53.2%	53.2%	53.7%	53.2%	53.2%	53.2%	52.7%	52.7%	53.2%	53.2%	53.2%	53.2%	53.2%	53.2%	53.2%	53.2%	53.2%	53.2%
2. ADQ39061.1 | - GU18...	54.9%	54.9%	54.9%	54.9%	54.9%	54.9%	54.9%	54.9%	55.3%	55.3%	54.9%	53.2%	53.2%	53.7%	53.2%	53.2%	53.2%	52.7%	52.7%	53.2%	53.2%	53.2%	53.2%	53.2%	53.2%	53.2%	53.2%	53.2%	53.2%
3. AUO29782.1 | KY636...	54.9%	54.9%	54.9%	54.9%	54.9%	54.9%	54.9%	54.9%	54.9%	54.9%	54.9%	53.7%	53.7%	54.1%	53.7%	53.7%	53.7%	53.2%	53.2%	53.7%	53.7%	53.7%	53.7%	53.7%	53.7%	53.7%	53.7%	53.7%	53.7%
5. ANV21438.1 | KU558...	54.9%	54.9%	54.9%	54.9%	54.9%	54.9%	54.9%	54.9%	54.9%	54.9%	54.9%	53.7%	53.7%	54.1%	53.7%	53.7%	53.7%	53.2%	53.2%	53.7%	53.7%	53.7%	53.7%	53.7%	53.7%	53.7%	53.7%	53.7%	53.7%
6. ANV21566.1 | KU569...	54.9%	54.9%	54.9%	54.9%	54.9%	54.9%	54.9%	54.9%	54.9%	54.9%	54.9%	53.7%	53.7%	54.1%	53.7%	53.7%	53.7%	53.2%	53.2%	53.7%	53.7%	53.7%	53.7%	53.7%	53.7%	53.7%	53.7%	53.7%	53.7%
7. AYU58961.1 | MH454...	54.9%	54.9%	54.9%	54.9%	54.9%	54.9%	54.9%	54.9%	54.9%	54.9%	54.9%	53.7%	53.7%	54.1%	53.7%	53.7%	53.7%	53.2%	53.2%	53.7%	53.7%	53.7%	53.7%	53.7%	53.7%	53.7%	53.7%	53.7%	53.7%
8. QNI22031.1 | MN781...	54.9%	54.9%	54.9%	54.9%	54.9%	54.9%	54.9%	54.9%	54.9%	54.9%	54.9%	53.7%	53.7%	54.1%	53.7%	53.7%	53.7%	53.2%	53.2%	53.7%	53.7%	53.7%	53.7%	53.7%	53.7%	53.7%	53.7%	53.7%	53.7%
9. CCE39367.1 | HE608...	54.9%	54.9%	54.9%	54.9%	54.9%	54.9%	54.9%	54.9%	55.3%	55.3%	54.9%	53.2%	53.2%	53.7%	53.2%	53.2%	53.2%	52.7%	52.7%	53.2%	53.2%	53.2%	53.2%	53.2%	53.2%	53.2%	53.2%	53.2%	53.2%
10. QJZ28073.1 | MN54...	55.3%	55.3%	55.3%	55.3%	55.3%	55.3%	55.3%	55.3%	55.3%	55.3%	55.3%	54.6%	54.6%	54.1%	51.7%	51.7%	51.7%	51.2%	51.2%	51.7%	51.7%	51.7%	51.7%	51.7%	51.7%	51.7%	51.7%	51.7%	51.7%
11. NP NC_001720.1 - A...	60.3%	60.3%	60.3%	60.3%	60.3%	60.3%	60.3%	60.3%	60.3%	60.3%	60.3%	61.3%	61.3%	61.8%	59.3%	59.3%	59.3%	57.8%	57.8%	59.3%	59.3%	59.3%	59.3%	59.3%	59.3%	59.3%	59.3%	59.3%	59.3%
12. AAC54912.1 | U469...	60.3%	60.3%	60.3%	60.3%	60.3%	60.3%	60.3%	60.3%	60.3%	60.3%	60.3%	61.3%	61.3%	61.8%	59.3%	59.3%	59.3%	57.8%	57.8%	59.3%	59.3%	59.3%	59.3%	59.3%	59.3%	59.3%	59.3%	59.3%	59.3%
13. APP94097.1 | KX247...	60.8%	60.8%	60.8%	60.8%	60.8%	60.8%	60.8%	60.8%	60.8%	60.8%	60.8%	61.8%	61.8%	62.3%	59.8%	59.8%	59.8%	58.3%	58.3%	59.8%	59.8%	59.8%	59.8%	59.8%	59.8%	59.8%	59.8%	59.8%	59.8%
14. YP NC_021221.1 NO...	68.4%	68.4%	68.4%	68.4%	68.4%	68.4%	68.4%	68.4%	68.4%	68.4%	68.4%	67.5%	67.5%	68.4%	68.4%	68.4%	68.4%	67.0%	67.0%	68.4%	68.4%	68.4%	68.4%	68.4%	68.4%	68.4%	68.4%	68.4%	68.4%
15. AGL34687.1 | KC493...	68.4%	68.4%	68.4%	68.4%	68.4%	68.4%	68.4%	68.4%	68.4%	68.4%	68.4%	67.5%	67.5%	68.4%	68.4%	68.4%	68.4%	67.0%	67.0%	68.4%	68.4%	68.4%	68.4%	68.4%	68.4%	68.4%	68.4%	68.4%	68.4%
16. QCC26479.1 | MG95...	68.4%	68.4%	68.4%	68.4%	68.4%	68.4%	68.4%	68.4%	68.4%	68.4%	68.4%	69.4%	69.4%	68.9%	67.5%	67.5%	67.5%	67.0%	67.0%	67.5%	67.5%	67.5%	67.5%	67.5%	67.5%	67.5%	67.5%	67.5%	67.5%
17. UDLA 71 ECUAD...	87.3%	87.3%	87.3%	87.3%	87.3%	87.3%	87.3%	87.3%	87.3%	87.3%	87.3%	78.4%	78.4%	79.9%	77.9%	77.9%	77.9%	77.5%	77.5%	77.9%	77.9%	77.9%	77.9%	77.9%	77.9%	77.9%	77.9%	77.9%	77.9%
18. ASU91620.1 |MF161...	87.3%	87.3%	87.3%	87.3%	87.3%	87.3%	87.3%	87.3%	87.3%	87.3%	87.3%	78.4%	78.4%	79.9%	77.9%	77.9%	77.9%	77.5%	77.5%	77.9%	77.9%	77.9%	77.9%	77.9%	77.9%	77.9%	77.9%	77.9%	77.9%
19. ANJ02455.1 | KT862...	87.3%	87.3%	87.3%	87.3%	87.3%	87.3%	87.3%	87.3%	87.3%	87.3%	87.3%	78.4%	78.4%	79.9%	77.9%	77.9%	77.9%	77.5%	77.5%	77.9%	77.9%	77.9%	77.9%	77.9%	77.9%	77.9%	77.9%	77.9%	77.9%
20. YP NC_038332.1 JAP...	87.3%	87.3%	87.3%	87.3%	87.3%	87.3%	87.3%	87.3%	87.3%	87.3%	87.3%	78.4%	78.4%	79.9%	77.9%	77.9%	77.9%	77.5%	77.5%	77.9%	77.9%	77.9%	77.9%	77.9%	77.9%	77.9%	77.9%	77.9%	77.9%
21. YP NC_014969.1 CA...	84.3%	84.3%	84.3%	84.3%	84.3%	84.3%	84.3%	84.3%	84.8%	84.8%	84.3%	75.5%	75.5%	76.5%	75.5%	75.5%	75.5%	75.0%	75.0%	75.5%	75.5%	75.5%	75.5%	75.5%	75.5%	75.5%	75.5%	75.5%	75.5%
22. ADE58399.1 |GU73...	84.3%	84.3%	84.3%	84.3%	84.3%	84.3%	84.3%	84.3%	84.8%	84.8%	84.3%	75.5%	75.5%	76.5%	75.5%	75.5%	75.5%	75.0%	75.0%	75.5%	75.5%	75.5%	75.5%	75.5%	75.5%	75.5%	75.5%	75.5%	75.5%
23. AOC84071.1 | KX25...	83.8%	83.8%	83.8%	83.8%	83.8%	83.8%	83.8%	83.8%	84.3%	84.3%	83.8%	76.0%	76.0%	77.5%	76.0%	76.0%	76.0%	75.5%	75.5%	76.0%	76.0%	76.0%	76.0%	76.0%	76.0%	76.0%	76.0%	76.0%	76.0%
25. AQX17370.1 | KY229...	84.8%	84.8%	84.8%	84.8%	84.8%	84.8%	84.8%	84.8%	85.3%	85.3%	84.8%	76.0%	76.0%	77.0%	76.0%	76.0%	76.0%	75.5%	75.5%	76.0%	76.0%	76.0%	76.0%	76.0%	76.0%	76.0%	76.0%	76.0%	76.0%
26. QIZ86862.1 | MT104...	83.3%	83.3%	83.3%	83.3%	83.3%	83.3%	83.3%	83.3%	83.8%	83.8%	83.3%	75.5%	75.5%	77.0%	75.5%	75.5%	75.5%	75.0%	75.0%	75.5%	75.5%	75.5%	75.5%	75.5%	75.5%	75.5%	75.5%	75.5%	75.5%
27. ANJ02566.1 | KT862...	85.3%	85.3%	85.3%	85.3%	85.3%	85.3%	85.3%	85.3%	85.8%	85.8%	85.3%	76.0%	76.0%	77.0%	75.5%	75.5%	75.5%	75.0%	75.0%	75.5%	75.5%	75.5%	75.5%	75.5%	75.5%	75.5%	75.5%	75.5%	75.5%
28. ANJ02492.1 |KT8628...	85.3%	85.3%	85.3%	85.3%	85.3%	85.3%	85.3%	85.3%	85.8%	85.8%	85.3%	76.5%	76.5%	77.9%	77.0%	77.0%	77.0%	76.5%	76.5%	77.0%	77.0%	77.0%	77.0%	77.0%	77.0%	77.0%	77.0%	77.0%	77.0%
30. UDLA 4 ECUADOR	100%	100%	100%	100%	100%	100%	100%	100%	99.5%	99.5%	100%	77.0%	77.0%	78.4%	78.9%	78.9%	78.9%	78.4%	78.4%	78.9%	78.9%	78.9%	78.9%	78.9%	78.9%	78.9%	78.9%	78.9%	78.9%
31. UDLA 17 ECUADOR	100%	100%	100%	100%	100%	100%	100%	100%	99.5%	99.5%	100%	77.0%	77.0%	78.4%	78.9%	78.9%	78.9%	78.4%	78.4%	78.9%	78.9%	78.9%	78.9%	78.9%	78.9%	78.9%	78.9%	78.9%	78.9%
34. UDLA 310-2 ECUAD...		100%	100%	100%	100%	100%	100%	100%	99.5%	99.5%	100%	77.0%	77.0%	78.4%	78.9%	78.9%	78.9%	78.4%	78.4%	78.9%	78.9%	78.9%	78.9%	78.9%	78.9%	78.9%	78.9%	78.9%	78.9%
35. UDLA 292-2 ECUA...	100%		100%	100%	100%	100%	100%	100%	99.5%	99.5%	100%	77.0%	77.0%	78.4%	78.9%	78.9%	78.9%	78.4%	78.4%	78.9%	78.9%	78.9%	78.9%	78.9%	78.9%	78.9%	78.9%	78.9%	78.9%
36. UDLA 282 ECUADOR	100%	100%		100%	100%	100%	100%	100%	99.5%	99.5%	100%	77.0%	77.0%	78.4%	78.9%	78.9%	78.9%	78.4%	78.4%	78.9%	78.9%	78.9%	78.9%	78.9%	78.9%	78.9%	78.9%	78.9%	78.9%
37. UDLA 316 ECUADOR	100%	100%	100%		100%	100%	100%	100%	99.5%	99.5%	100%	77.0%	77.0%	78.4%	78.9%	78.9%	78.9%	78.4%	78.4%	78.9%	78.9%	78.9%	78.9%	78.9%	78.9%	78.9%	78.9%	78.9%	78.9%
38. UDLA 310 ECUADOR	100%	100%	100%	100%		100%	100%	100%	99.5%	99.5%	100%	77.0%	77.0%	78.4%	78.9%	78.9%	78.9%	78.4%	78.4%	78.9%	78.9%	78.9%	78.9%	78.9%	78.9%	78.9%	78.9%	78.9%	78.9%
39. UDLA 291 ECUADOR	100%	100%	100%	100%	100%		100%	100%	99.5%	99.5%	100%	77.0%	77.0%	78.4%	78.9%	78.9%	78.9%	78.4%	78.4%	78.9%	78.9%	78.9%	78.9%	78.9%	78.9%	78.9%	78.9%	78.9%	78.9%
40. UDLA 292 ECUADOR	100%	100%	100%	100%	100%	100%		100%	99.5%	99.5%	100%	77.0%	77.0%	78.4%	78.9%	78.9%	78.9%	78.4%	78.4%	78.9%	78.9%	78.9%	78.9%	78.9%	78.9%	78.9%	78.9%	78.9%	78.9%
41. UDLA 303 ECUADOR	100%	100%	100%	100%	100%	100%	100%		99.5%	99.5%	100%	77.0%	77.0%	78.4%	78.9%	78.9%	78.9%	78.4%	78.4%	78.9%	78.9%	78.9%	78.9%	78.9%	78.9%	78.9%	78.9%	78.9%	78.9%
42. QKE53464.1 | MN45...	99.5%	99.5%	99.5%	99.5%	99.5%	99.5%	99.5%	99.5%		100%	99.5%	77.0%	77.0%	78.4%	78.4%	78.4%	78.4%	77.9%	77.9%	78.4%	78.4%	78.4%	78.4%	78.4%	78.4%	78.4%	78.4%	78.4%	78.4%
43. AQX17379.1 | KY229...	99.5%	99.5%	99.5%	99.5%	99.5%	99.5%	99.5%	99.5%	100%		99.5%	77.0%	77.0%	78.4%	78.4%	78.4%	78.4%	77.9%	77.9%	78.4%	78.4%	78.4%	78.4%	78.4%	78.4%	78.4%	78.4%	78.4%	78.4%
44. ABD83864.1 | DQ32...	100%	100%	100%	100%	100%	100%	100%	100%	99.5%	99.5%		77.0%	77.0%	78.4%	78.9%	78.9%	78.9%	78.4%	78.4%	78.9%	78.9%	78.9%	78.9%	78.9%	78.9%	78.9%	78.9%	78.9%	78.9%
46. NP NC_000899.1 PO...	77.0%	77.0%	77.0%	77.0%	77.0%	77.0%	77.0%	77.0%	77.0%	77.0%	77.0%		100%	91.6%	78.8%	78.8%	78.8%	77.3%	77.3%	78.8%	78.8%	78.8%	78.8%	78.8%	78.8%	78.8%	78.8%	78.8%	78.8%
47. AAD50344.2 |AF083...	77.0%	77.0%	77.0%	77.0%	77.0%	77.0%	77.0%	77.0%	77.0%	77.0%	77.0%	100%		91.6%	78.8%	78.8%	78.8%	77.3%	77.3%	78.8%	78.8%	78.8%	78.8%	78.8%	78.8%	78.8%	78.8%	78.8%	78.8%
48. ANJ02418.1 | KT862...	78.4%	78.4%	78.4%	78.4%	78.4%	78.4%	78.4%	78.4%	78.4%	78.4%	78.4%	91.6%	91.6%		79.3%	79.3%	79.3%	77.8%	77.8%	79.3%	79.3%	79.3%	79.3%	79.3%	79.3%	79.3%	79.3%	79.3%	79.3%
49. UDLA 2 ECUADOR	78.9%	78.9%	78.9%	78.9%	78.9%	78.9%	78.9%	78.9%	78.4%	78.4%	78.9%	78.8%	78.8%	79.3%		100%	100%	98.5%	98.5%	100%	100%	100%	100%	100%	100%	100%	100%	100%	100%
50. UDLA 3 ECUADOR	78.9%	78.9%	78.9%	78.9%	78.9%	78.9%	78.9%	78.9%	78.4%	78.4%	78.9%	78.8%	78.8%	79.3%	100%		100%	98.5%	98.5%	100%	100%	100%	100%	100%	100%	100%	100%	100%	100%
51. UDLA 72 ECUADOR	78.9%	78.9%	78.9%	78.9%	78.9%	78.9%	78.9%	78.9%	78.4%	78.4%	78.9%	78.8%	78.8%	79.3%	100%	100%		98.5%	98.5%	100%	100%	100%	100%	100%	100%	100%	100%	100%	100%
52. UDLA 76 ECUADOR	78.4%	78.4%	78.4%	78.4%	78.4%	78.4%	78.4%	78.4%	77.9%	77.9%	78.4%	77.3%	77.3%	77.8%	98.5%	98.5%	98.5%		100%	98.5%	98.5%	98.5%	98.5%	98.5%	98.5%	98.5%	98.5%	98.5%	98.5%
53. UDLA 78 ECUADOR	78.4%	78.4%	78.4%	78.4%	78.4%	78.4%	78.4%	78.4%	77.9%	77.9%	78.4%	77.3%	77.3%	77.8%	98.5%	98.5%	98.5%	100%		98.5%	98.5%	98.5%	98.5%	98.5%	98.5%	98.5%	98.5%	98.5%	98.5%
54. UDLA 80 ECUADOR	78.9%	78.9%	78.9%	78.9%	78.9%	78.9%	78.9%	78.9%	78.4%	78.4%	78.9%	78.8%	78.8%	79.3%	100%	100%	100%	98.5%	98.5%		100%	100%	100%	100%	100%	100%	100%	100%	100%
55. UDLA 226 ECUADOR	78.9%	78.9%	78.9%	78.9%	78.9%	78.9%	78.9%	78.9%	78.4%	78.4%	78.9%	78.8%	78.8%	79.3%	100%	100%	100%	98.5%	98.5%	100%		100%	100%	100%	100%	100%	100%	100%	100%
56. UDLA 222 ECUADOR	78.9%	78.9%	78.9%	78.9%	78.9%	78.9%	78.9%	78.9%	78.4%	78.4%	78.9%	78.8%	78.8%	79.3%	100%	100%	100%	98.5%	98.5%	100%	100%		100%	100%	100%	100%	100%	100%	100%
57. UDLA 213 ECUADOR	78.9%	78.9%	78.9%	78.9%	78.9%	78.9%	78.9%	78.9%	78.4%	78.4%	78.9%	78.8%	78.8%	79.3%	100%	100%	100%	98.5%	98.5%	100%	100%	100%		100%	100%	100%	100%	100%	100%
58. UDLA 210 ECUADOR	78.9%	78.9%	78.9%	78.9%	78.9%	78.9%	78.9%	78.9%	78.4%	78.4%	78.9%	78.8%	78.8%	79.3%	100%	100%	100%	98.5%	98.5%	100%	100%	100%	100%		100%	100%	100%	100%	100%
59. UDLA 85 ECUADOR	78.9%	78.9%	78.9%	78.9%	78.9%	78.9%	78.9%	78.9%	78.4%	78.4%	78.9%	78.8%	78.8%	79.3%	100%	100%	100%	98.5%	98.5%	100%	100%	100%	100%	100%		100%	100%	100%	100%
60. UDLA 250 ECUADOR	78.9%	78.9%	78.9%	78.9%	78.9%	78.9%	78.9%	78.9%	78.4%	78.4%	78.9%	78.8%	78.8%	79.3%	100%	100%	100%	98.5%	98.5%	100%	100%	100%	100%	100%	100%		100%	100%	100%
61. UDLA 86-2 ECUAD...	78.9%	78.9%	78.9%	78.9%	78.9%	78.9%	78.9%	78.9%	78.4%	78.4%	78.9%	78.8%	78.8%	79.3%	100%	100%	100%	98.5%	98.5%	100%	100%	100%	100%	100%	100%	100%		100%	100%
62. UDLA 88 ECUADOR	78.9%	78.9%	78.9%	78.9%	78.9%	78.9%	78.9%	78.9%	78.4%	78.4%	78.9%	78.8%	78.8%	79.3%	100%	100%	100%	98.5%	98.5%	100%	100%	100%	100%	100%	100%	100%	100%		100%
63. UDLA 207 ECUADOR	78.9%	78.9%	78.9%	78.9%	78.9%	78.9%	78.9%	78.9%	78.4%	78.4%	78.9%	78.8%	78.8%	79.3%	100%	100%	100%	98.5%	98.5%	100%	100%	100%	100%	100%	100%	100%	100%	100%	
64. UDLA 76F ECUADOR	78.9%	78.9%	78.9%	78.9%	78.9%	78.9%	78.9%	78.9%	78.4%	78.4%	78.9%	78.8%	78.8%	79.3%	100%	100%	100%	98.5%	98.5%	100%	100%	100%	100%	100%	100%	100%	100%	100%	100%
65. UDLA 276 ECUADOR	78.9%	78.9%	78.9%	78.9%	78.9%	78.9%	78.9%	78.9%	78.4%	78.4%	78.9%	78.8%	78.8%	79.3%	100%	100%	100%	98.5%	98.5%	100%	100%	100%	100%	100%	100%	100%	100%	100%	100%
66. UDLA 268 ECUADOR	78.9%	78.9%	78.9%	78.9%	78.9%	78.9%	78.9%	78.9%	78.4%	78.4%	78.9%	78.8%	78.8%	79.3%	100%	100%	100%	98.5%	98.5%	100%	100%	100%	100%	100%	100%	100%	100%	100%	100%
67. UDLA 257 ECUADOR	78.9%	78.9%	78.9%	78.9%	78.9%	78.9%	78.9%	78.9%	78.4%	78.4%	78.9%	78.8%	78.8%	79.3%	100%	100%	100%	98.5%	98.5%	100%	100%	100%	100%	100%	100%	100%	100%	100%	100%
68. UDLA 247 ECUADOR	78.9%	78.9%	78.9%	78.9%	78.9%	78.9%	78.9%	78.9%	78.4%	78.4%	78.9%	78.8%	78.8%	79.3%	100%	100%	100%	98.5%	98.5%	100%	100%	100%	100%	100%	100%	100%	100%	100%	100%
69. 267 ECUADOR	78.9%	78.9%	78.9%	78.9%	78.9%	78.9%	78.9%	78.9%	78.4%	78.4%	78.9%	78.8%	78.8%	79.3%	100%	100%	100%	98.5%	98.5%	100%	100%	100%	100%	100%	100%	100%	100%	100%	100%
70. UDLA 280 ECUADOR	78.9%	78.9%	78.9%	78.9%	78.9%	78.9%	78.9%	78.9%	78.4%	78.4%	78.9%	78.8%	78.8%	79.3%	100%	100%	100%	98.5%	98.5%	100%	100%	100%	100%	100%	100%	100%	100%	100%	100%
71. UDLA 299 ECUADOR	78.9%	78.9%	78.9%	78.9%	78.9%	78.9%	78.9%	78.9%	78.4%	78.4%	78.9%	78.8%	78.8%	79.3%	100%	100%	100%	98.5%	98.5%	100%	100%	100%	100%	100%	100%	100%	100%	100%	100%
72. UDLA 86 ECUADOR	78.9%	78.9%	78.9%	78.9%	78.9%	78.9%	78.9%	78.9%	78.4%	78.4%	78.9%	78.8%	78.8%	79.3%	100%	100%	100%	98.5%	98.5%	100%	100%	100%	100%	100%	100%	100%	100%	100%	100%
73. UDLA 203 ECUADOR	78.9%	78.9%	78.9%	78.9%	78.9%	78.9%	78.9%	78.9%	78.4%	78.4%	78.9%	78.8%	78.8%	79.3%	100%	100%	100%	98.5%	98.5%	100%	100%	100%	100%	100%	100%	100%	100%	100%	100%
74. ABD83863.1 | DQ32...	79.4%	79.4%	79.4%	79.4%	79.4%	79.4%	79.4%	79.4%	78.9%	78.9%	79.4%	78.3%	78.3%	78.8%	99.5%	99.5%	99.5%	99.0%	99.0%	99.5%	99.5%	99.5%	99.5%	99.5%	99.5%	99.5%	99.5%	99.5%	99.5%
75. AOS51012.1 |KU746...	79.4%	79.4%	79.4%	79.4%	79.4%	79.4%	79.4%	79.4%	78.9%	78.9%	79.4%	78.3%	78.3%	78.8%	99.5%	99.5%	99.5%	99.0%	99.0%	99.5%	99.5%	99.5%	99.5%	99.5%	99.5%	99.5%	99.5%	99.5%	99.5%
76. AKR76197.1 |KP231...	79.4%	79.4%	79.4%	79.4%	79.4%	79.4%	79.4%	79.4%	78.9%	78.9%	79.4%	78.3%	78.3%	78.8%	99.5%	99.5%	99.5%	99.0%	99.0%	99.5%	99.5%	99.5%	99.5%	99.5%	99.5%	99.5%	99.5%	99.5%	99.5%
77. ASU91621.1 | MF16...	79.4%	79.4%	79.4%	79.4%	79.4%	79.4%	79.4%	79.4%	78.9%	78.9%	79.4%	78.3%	78.3%	78.8%	99.5%	99.5%	99.5%	99.0%	99.0%	99.5%	99.5%	99.5%	99.5%	99.5%	99.5%	99.5%	99.5%	99.5%	99.5%
78. QIM09460.1 |MN42...	79.4%	79.4%	79.4%	79.4%	79.4%	79.4%	79.4%	79.4%	78.9%	78.9%	79.4%	77.8%	77.8%	78.3%	99.0%	99.0%	99.0%	98.5%	98.5%	99.0%	99.0%	99.0%	99.0%	99.0%	99.0%	99.0%	99.0%	99.0%	99.0%
79. QFR45466.1 |MK757...	79.4%	79.4%	79.4%	79.4%	79.4%	79.4%	79.4%	79.4%	78.9%	78.9%	79.4%	78.3%	78.3%	78.8%	99.5%	99.5%	99.5%	99.0%	99.0%	99.5%	99.5%	99.5%	99.5%	99.5%	99.5%	99.5%	99.5%	99.5%	99.5%
81. UDLA 50 ECUADOR	79.9%	79.9%	79.9%	79.9%	79.9%	79.9%	79.9%	79.9%	79.4%	79.4%	79.9%	79.3%	79.3%	78.8%	93.1%	93.1%	93.1%	92.6%	92.6%	93.1%	93.1%	93.1%	93.1%	93.1%	93.1%	93.1%	93.1%	93.1%	93.1%
82. AQX17377.1 | MT10...	79.4%	79.4%	79.4%	79.4%	79.4%	79.4%	79.4%	79.4%	78.9%	78.9%	79.4%	78.3%	78.3%	78.8%	99.5%	99.5%	99.5%	99.0%	99.0%	99.5%	99.5%	99.5%	99.5%	99.5%	99.5%	99.5%	99.5%	99.5%	99.5%
83. UDLA 337 ECUADOR	79.9%	79.9%	79.9%	79.9%	79.9%	79.9%	79.9%	79.9%	79.4%	79.4%	79.9%	79.3%	79.3%	78.8%	93.1%	93.1%	93.1%	92.6%	92.6%	93.1%	93.1%	93.1%	93.1%	93.1%	93.1%	93.1%	93.1%	93.1%	93.1%
84. UDLA 64 ECUADOR	79.9%	79.9%	79.9%	79.9%	79.9%	79.9%	79.9%	79.9%	79.4%	79.4%	79.9%	79.3%	79.3%	78.8%	93.1%	93.1%	93.1%	92.6%	92.6%	93.1%	93.1%	93.1%	93.1%	93.1%	93.1%	93.1%	93.1%	93.1%	93.1%
85. UDLA 56 ECUADOR	79.9%	79.9%	79.9%	79.9%	79.9%	79.9%	79.9%	79.9%	79.4%	79.4%	79.9%	79.3%	79.3%	78.8%	93.1%	93.1%	93.1%	92.6%	92.6%	93.1%	93.1%	93.1%	93.1%	93.1%	93.1%	93.1%	93.1%	93.1%	93.1%
86. UDLA 44 ECUADOR	79.9%	79.9%	79.9%	79.9%	79.9%	79.9%	79.9%	79.9%	79.4%	79.4%	79.9%	79.3%	79.3%	78.8%	93.1%	93.1%	93.1%	92.6%	92.6%	93.1%	93.1%	93.1%	93.1%	93.1%	93.1%	93.1%	93.1%	93.1%	93.1%
87. ANJ02344.1 | KT862...	79.9%	79.9%	79.9%	79.9%	79.9%	79.9%	79.9%	79.9%	79.4%	79.4%	79.9%	79.3%	79.3%	78.8%	93.1%	93.1%	93.1%	92.6%	92.6%	93.1%	93.1%	93.1%	93.1%	93.1%	93.1%	93.1%	93.1%	93.1%	93.1%
89. NP NC_001813.1 D...	32.9%	32.9%	32.9%	32.9%	32.9%	32.9%	32.9%	32.9%	32.9%	32.9%	32.9%	31.1%	31.1%	32.1%	30.1%	30.1%	30.1%	30.6%	30.6%	30.1%	30.1%	30.1%	30.1%	30.1%	30.1%	30.1%	30.1%	30.1%	30.1%

	64. UDL...	65. UDL...	66. UDL...	67. UDL...	68. UDL...	69. 267...	70. UDL...	71. UDL...	72. UDL...	73. UDL...	74. ABD...	75. AOS...	76. AKR...	77. ASU...	78. QIM...	79. QFR...	81. UDL...	82. AQX...	83. UDL...	84. UDL...	85. UDL...	86. UDL...	87. ANJ...	89. NP ...
1. YP - NC_015323 C...	53.2%	53.2%	53.2%	53.2%	53.2%	53.2%	53.2%	53.2%	53.2%	53.2%	53.2%	53.2%	53.2%	53.2%	53.2%	53.2%	53.2%	53.2%	53.2%	53.2%	53.2%	53.2%	53.2%	32.4%
2. ADQ39061.1 | - GU18...	53.2%	53.2%	53.2%	53.2%	53.2%	53.2%	53.2%	53.2%	53.2%	53.2%	53.2%	53.2%	53.2%	53.2%	53.2%	53.2%	53.2%	53.2%	53.2%	53.2%	53.2%	53.2%	53.2%	32.4%
3. AUO29782.1 | KY636...	53.7%	53.7%	53.7%	53.7%	53.7%	53.7%	53.7%	53.7%	53.7%	53.7%	53.7%	53.7%	53.7%	53.7%	53.7%	53.7%	52.7%	53.7%	52.7%	52.7%	52.7%	52.7%	52.7%	32.9%
5. ANV21438.1 | KU558...	53.7%	53.7%	53.7%	53.7%	53.7%	53.7%	53.7%	53.7%	53.7%	53.7%	53.7%	53.7%	53.7%	53.7%	53.7%	53.7%	52.7%	53.7%	52.7%	52.7%	52.7%	52.7%	52.7%	32.9%
6. ANV21566.1 | KU569...	53.7%	53.7%	53.7%	53.7%	53.7%	53.7%	53.7%	53.7%	53.7%	53.7%	53.7%	53.7%	53.7%	53.7%	53.7%	53.7%	52.7%	53.7%	52.7%	52.7%	52.7%	52.7%	52.7%	32.9%
7. AYU58961.1 | MH454...	53.7%	53.7%	53.7%	53.7%	53.7%	53.7%	53.7%	53.7%	53.7%	53.7%	53.7%	53.7%	53.7%	53.7%	53.7%	53.7%	52.7%	53.7%	52.7%	52.7%	52.7%	52.7%	52.7%	32.9%
8. QNI22031.1 | MN781...	53.7%	53.7%	53.7%	53.7%	53.7%	53.7%	53.7%	53.7%	53.7%	53.7%	53.7%	53.7%	53.7%	53.7%	53.7%	53.7%	52.7%	53.7%	52.7%	52.7%	52.7%	52.7%	52.7%	32.9%
9. CCE39367.1 | HE608...	53.2%	53.2%	53.2%	53.2%	53.2%	53.2%	53.2%	53.2%	53.2%	53.2%	53.2%	53.2%	53.2%	53.2%	53.2%	53.2%	53.2%	53.2%	53.2%	53.2%	53.2%	53.2%	53.2%	32.4%
10. QJZ28073.1 | MN54...	51.7%	51.7%	51.7%	51.7%	51.7%	51.7%	51.7%	51.7%	51.7%	51.7%	51.7%	51.7%	51.7%	51.7%	51.7%	51.7%	53.2%	51.7%	53.2%	53.2%	53.2%	53.2%	53.2%	32.4%
11. NP NC_001720.1 - A...	59.3%	59.3%	59.3%	59.3%	59.3%	59.3%	59.3%	59.3%	59.3%	59.3%	58.8%	58.8%	58.8%	58.8%	58.8%	58.8%	58.3%	58.8%	58.3%	58.3%	58.3%	58.3%	58.3%	31.1%
12. AAC54912.1 | U469...	59.3%	59.3%	59.3%	59.3%	59.3%	59.3%	59.3%	59.3%	59.3%	59.3%	58.8%	58.8%	58.8%	58.8%	58.8%	58.8%	58.3%	58.8%	58.3%	58.3%	58.3%	58.3%	58.3%	31.1%
13. APP94097.1 | KX247...	59.8%	59.8%	59.8%	59.8%	59.8%	59.8%	59.8%	59.8%	59.8%	59.8%	59.3%	59.3%	59.3%	59.3%	59.3%	59.3%	58.8%	59.3%	58.8%	58.8%	58.8%	58.8%	58.8%	31.1%
14. YP NC_021221.1 NO...	68.4%	68.4%	68.4%	68.4%	68.4%	68.4%	68.4%	68.4%	68.4%	68.4%	68.0%	68.0%	68.0%	68.0%	68.0%	68.0%	68.0%	68.0%	68.0%	68.0%	68.0%	68.0%	68.0%	31.6%
15. AGL34687.1 | KC493...	68.4%	68.4%	68.4%	68.4%	68.4%	68.4%	68.4%	68.4%	68.4%	68.4%	68.0%	68.0%	68.0%	68.0%	68.0%	68.0%	68.0%	68.0%	68.0%	68.0%	68.0%	68.0%	68.0%	31.6%
16. QCC26479.1 | MG95...	67.5%	67.5%	67.5%	67.5%	67.5%	67.5%	67.5%	67.5%	67.5%	67.5%	68.0%	68.0%	68.0%	68.0%	68.0%	68.0%	69.9%	68.0%	69.9%	69.9%	69.9%	69.9%	69.9%	30.2%
17. UDLA 71 ECUAD...	77.9%	77.9%	77.9%	77.9%	77.9%	77.9%	77.9%	77.9%	77.9%	77.9%	78.4%	78.4%	78.4%	78.4%	78.4%	78.4%	79.4%	78.4%	79.4%	79.4%	79.4%	79.4%	79.4%	31.9%
18. ASU91620.1 |MF161...	77.9%	77.9%	77.9%	77.9%	77.9%	77.9%	77.9%	77.9%	77.9%	77.9%	78.4%	78.4%	78.4%	78.4%	78.4%	78.4%	79.4%	78.4%	79.4%	79.4%	79.4%	79.4%	79.4%	31.9%
19. ANJ02455.1 | KT862...	77.9%	77.9%	77.9%	77.9%	77.9%	77.9%	77.9%	77.9%	77.9%	77.9%	78.4%	78.4%	78.4%	78.4%	78.4%	78.4%	79.4%	78.4%	79.4%	79.4%	79.4%	79.4%	79.4%	31.9%
20. YP NC_038332.1 JAP...	77.9%	77.9%	77.9%	77.9%	77.9%	77.9%	77.9%	77.9%	77.9%	77.9%	78.4%	78.4%	78.4%	78.4%	78.4%	78.4%	79.4%	78.4%	79.4%	79.4%	79.4%	79.4%	79.4%	31.9%
21. YP NC_014969.1 CA...	75.5%	75.5%	75.5%	75.5%	75.5%	75.5%	75.5%	75.5%	75.5%	75.5%	76.0%	76.0%	76.0%	76.0%	76.0%	76.0%	76.5%	76.0%	76.5%	76.5%	76.5%	76.5%	76.5%	33.8%
22. ADE58399.1 |GU73...	75.5%	75.5%	75.5%	75.5%	75.5%	75.5%	75.5%	75.5%	75.5%	75.5%	76.0%	76.0%	76.0%	76.0%	76.0%	76.0%	76.5%	76.0%	76.5%	76.5%	76.5%	76.5%	76.5%	33.8%
23. AOC84071.1 | KX25...	76.0%	76.0%	76.0%	76.0%	76.0%	76.0%	76.0%	76.0%	76.0%	76.0%	76.5%	76.5%	76.5%	76.5%	76.5%	76.5%	76.5%	76.5%	76.5%	76.5%	76.5%	76.5%	76.5%	33.3%
25. AQX17370.1 | KY229...	76.0%	76.0%	76.0%	76.0%	76.0%	76.0%	76.0%	76.0%	76.0%	76.0%	76.5%	76.5%	76.5%	76.5%	76.5%	76.5%	77.0%	76.5%	77.0%	77.0%	77.0%	77.0%	77.0%	33.8%
26. QIZ86862.1 | MT104...	75.5%	75.5%	75.5%	75.5%	75.5%	75.5%	75.5%	75.5%	75.5%	75.5%	76.0%	76.0%	76.0%	76.0%	76.0%	76.0%	76.5%	76.0%	76.5%	76.5%	76.5%	76.5%	76.5%	32.4%
27. ANJ02566.1 | KT862...	75.5%	75.5%	75.5%	75.5%	75.5%	75.5%	75.5%	75.5%	75.5%	75.5%	76.0%	76.0%	76.0%	76.0%	76.0%	76.0%	76.5%	76.0%	76.5%	76.5%	76.5%	76.5%	76.5%	33.8%
28. ANJ02492.1 |KT8628...	77.0%	77.0%	77.0%	77.0%	77.0%	77.0%	77.0%	77.0%	77.0%	77.0%	77.5%	77.5%	77.5%	77.5%	77.5%	77.5%	77.9%	77.5%	77.9%	77.9%	77.9%	77.9%	77.9%	32.1%
30. UDLA 4 ECUADOR	78.9%	78.9%	78.9%	78.9%	78.9%	78.9%	78.9%	78.9%	78.9%	78.9%	79.4%	79.4%	79.4%	79.4%	79.4%	79.4%	79.9%	79.4%	79.9%	79.9%	79.9%	79.9%	79.9%	32.9%
31. UDLA 17 ECUADOR	78.9%	78.9%	78.9%	78.9%	78.9%	78.9%	78.9%	78.9%	78.9%	78.9%	79.4%	79.4%	79.4%	79.4%	79.4%	79.4%	79.9%	79.4%	79.9%	79.9%	79.9%	79.9%	79.9%	32.9%
34. UDLA 310-2 ECUAD...	78.9%	78.9%	78.9%	78.9%	78.9%	78.9%	78.9%	78.9%	78.9%	78.9%	79.4%	79.4%	79.4%	79.4%	79.4%	79.4%	79.9%	79.4%	79.9%	79.9%	79.9%	79.9%	79.9%	32.9%
35. UDLA 292-2 ECUA...	78.9%	78.9%	78.9%	78.9%	78.9%	78.9%	78.9%	78.9%	78.9%	78.9%	79.4%	79.4%	79.4%	79.4%	79.4%	79.4%	79.9%	79.4%	79.9%	79.9%	79.9%	79.9%	79.9%	32.9%
36. UDLA 282 ECUADOR	78.9%	78.9%	78.9%	78.9%	78.9%	78.9%	78.9%	78.9%	78.9%	78.9%	79.4%	79.4%	79.4%	79.4%	79.4%	79.4%	79.9%	79.4%	79.9%	79.9%	79.9%	79.9%	79.9%	32.9%
37. UDLA 316 ECUADOR	78.9%	78.9%	78.9%	78.9%	78.9%	78.9%	78.9%	78.9%	78.9%	78.9%	79.4%	79.4%	79.4%	79.4%	79.4%	79.4%	79.9%	79.4%	79.9%	79.9%	79.9%	79.9%	79.9%	32.9%
38. UDLA 310 ECUADOR	78.9%	78.9%	78.9%	78.9%	78.9%	78.9%	78.9%	78.9%	78.9%	78.9%	79.4%	79.4%	79.4%	79.4%	79.4%	79.4%	79.9%	79.4%	79.9%	79.9%	79.9%	79.9%	79.9%	32.9%
39. UDLA 291 ECUADOR	78.9%	78.9%	78.9%	78.9%	78.9%	78.9%	78.9%	78.9%	78.9%	78.9%	79.4%	79.4%	79.4%	79.4%	79.4%	79.4%	79.9%	79.4%	79.9%	79.9%	79.9%	79.9%	79.9%	32.9%
40. UDLA 292 ECUADOR	78.9%	78.9%	78.9%	78.9%	78.9%	78.9%	78.9%	78.9%	78.9%	78.9%	79.4%	79.4%	79.4%	79.4%	79.4%	79.4%	79.9%	79.4%	79.9%	79.9%	79.9%	79.9%	79.9%	32.9%
41. UDLA 303 ECUADOR	78.9%	78.9%	78.9%	78.9%	78.9%	78.9%	78.9%	78.9%	78.9%	78.9%	79.4%	79.4%	79.4%	79.4%	79.4%	79.4%	79.9%	79.4%	79.9%	79.9%	79.9%	79.9%	79.9%	32.9%
42. QKE53464.1 | MN45...	78.4%	78.4%	78.4%	78.4%	78.4%	78.4%	78.4%	78.4%	78.4%	78.4%	78.9%	78.9%	78.9%	78.9%	78.9%	78.9%	79.4%	78.9%	79.4%	79.4%	79.4%	79.4%	79.4%	32.9%
43. AQX17379.1 | KY229...	78.4%	78.4%	78.4%	78.4%	78.4%	78.4%	78.4%	78.4%	78.4%	78.4%	78.9%	78.9%	78.9%	78.9%	78.9%	78.9%	79.4%	78.9%	79.4%	79.4%	79.4%	79.4%	79.4%	32.9%
44. ABD83864.1 | DQ32...	78.9%	78.9%	78.9%	78.9%	78.9%	78.9%	78.9%	78.9%	78.9%	78.9%	79.4%	79.4%	79.4%	79.4%	79.4%	79.4%	79.9%	79.4%	79.9%	79.9%	79.9%	79.9%	79.9%	32.9%
46. NP NC_000899.1 PO...	78.8%	78.8%	78.8%	78.8%	78.8%	78.8%	78.8%	78.8%	78.8%	78.8%	78.3%	78.3%	78.3%	78.3%	77.8%	78.3%	79.3%	78.3%	79.3%	79.3%	79.3%	79.3%	79.3%	31.1%
47. AAD50344.2 |AF083...	78.8%	78.8%	78.8%	78.8%	78.8%	78.8%	78.8%	78.8%	78.8%	78.8%	78.3%	78.3%	78.3%	78.3%	77.8%	78.3%	79.3%	78.3%	79.3%	79.3%	79.3%	79.3%	79.3%	31.1%
48. ANJ02418.1 | KT862...	79.3%	79.3%	79.3%	79.3%	79.3%	79.3%	79.3%	79.3%	79.3%	79.3%	78.8%	78.8%	78.8%	78.8%	78.3%	78.8%	78.8%	78.8%	78.8%	78.8%	78.8%	78.8%	78.8%	32.1%
49. UDLA 2 ECUADOR	100%	100%	100%	100%	100%	100%	100%	100%	100%	100%	99.5%	99.5%	99.5%	99.5%	99.0%	99.5%	93.1%	99.5%	93.1%	93.1%	93.1%	93.1%	93.1%	30.1%
50. UDLA 3 ECUADOR	100%	100%	100%	100%	100%	100%	100%	100%	100%	100%	99.5%	99.5%	99.5%	99.5%	99.0%	99.5%	93.1%	99.5%	93.1%	93.1%	93.1%	93.1%	93.1%	30.1%
51. UDLA 72 ECUADOR	100%	100%	100%	100%	100%	100%	100%	100%	100%	100%	99.5%	99.5%	99.5%	99.5%	99.0%	99.5%	93.1%	99.5%	93.1%	93.1%	93.1%	93.1%	93.1%	30.1%
52. UDLA 76 ECUADOR	98.5%	98.5%	98.5%	98.5%	98.5%	98.5%	98.5%	98.5%	98.5%	98.5%	99.0%	99.0%	99.0%	99.0%	98.5%	99.0%	92.6%	99.0%	92.6%	92.6%	92.6%	92.6%	92.6%	30.6%
53. UDLA 78 ECUADOR	98.5%	98.5%	98.5%	98.5%	98.5%	98.5%	98.5%	98.5%	98.5%	98.5%	99.0%	99.0%	99.0%	99.0%	98.5%	99.0%	92.6%	99.0%	92.6%	92.6%	92.6%	92.6%	92.6%	30.6%
54. UDLA 80 ECUADOR	100%	100%	100%	100%	100%	100%	100%	100%	100%	100%	99.5%	99.5%	99.5%	99.5%	99.0%	99.5%	93.1%	99.5%	93.1%	93.1%	93.1%	93.1%	93.1%	30.1%
55. UDLA 226 ECUADOR	100%	100%	100%	100%	100%	100%	100%	100%	100%	100%	99.5%	99.5%	99.5%	99.5%	99.0%	99.5%	93.1%	99.5%	93.1%	93.1%	93.1%	93.1%	93.1%	30.1%
56. UDLA 222 ECUADOR	100%	100%	100%	100%	100%	100%	100%	100%	100%	100%	99.5%	99.5%	99.5%	99.5%	99.0%	99.5%	93.1%	99.5%	93.1%	93.1%	93.1%	93.1%	93.1%	30.1%
57. UDLA 213 ECUADOR	100%	100%	100%	100%	100%	100%	100%	100%	100%	100%	99.5%	99.5%	99.5%	99.5%	99.0%	99.5%	93.1%	99.5%	93.1%	93.1%	93.1%	93.1%	93.1%	30.1%
58. UDLA 210 ECUADOR	100%	100%	100%	100%	100%	100%	100%	100%	100%	100%	99.5%	99.5%	99.5%	99.5%	99.0%	99.5%	93.1%	99.5%	93.1%	93.1%	93.1%	93.1%	93.1%	30.1%
59. UDLA 85 ECUADOR	100%	100%	100%	100%	100%	100%	100%	100%	100%	100%	99.5%	99.5%	99.5%	99.5%	99.0%	99.5%	93.1%	99.5%	93.1%	93.1%	93.1%	93.1%	93.1%	30.1%
60. UDLA 250 ECUADOR	100%	100%	100%	100%	100%	100%	100%	100%	100%	100%	99.5%	99.5%	99.5%	99.5%	99.0%	99.5%	93.1%	99.5%	93.1%	93.1%	93.1%	93.1%	93.1%	30.1%
61. UDLA 86-2 ECUAD...	100%	100%	100%	100%	100%	100%	100%	100%	100%	100%	99.5%	99.5%	99.5%	99.5%	99.0%	99.5%	93.1%	99.5%	93.1%	93.1%	93.1%	93.1%	93.1%	30.1%
62. UDLA 88 ECUADOR	100%	100%	100%	100%	100%	100%	100%	100%	100%	100%	99.5%	99.5%	99.5%	99.5%	99.0%	99.5%	93.1%	99.5%	93.1%	93.1%	93.1%	93.1%	93.1%	30.1%
63. UDLA 207 ECUADOR	100%	100%	100%	100%	100%	100%	100%	100%	100%	100%	99.5%	99.5%	99.5%	99.5%	99.0%	99.5%	93.1%	99.5%	93.1%	93.1%	93.1%	93.1%	93.1%	30.1%
64. UDLA 76F ECUADOR		100%	100%	100%	100%	100%	100%	100%	100%	100%	99.5%	99.5%	99.5%	99.5%	99.0%	99.5%	93.1%	99.5%	93.1%	93.1%	93.1%	93.1%	93.1%	30.1%
65. UDLA 276 ECUADOR	100%		100%	100%	100%	100%	100%	100%	100%	100%	99.5%	99.5%	99.5%	99.5%	99.0%	99.5%	93.1%	99.5%	93.1%	93.1%	93.1%	93.1%	93.1%	30.1%
66. UDLA 268 ECUADOR	100%	100%		100%	100%	100%	100%	100%	100%	100%	99.5%	99.5%	99.5%	99.5%	99.0%	99.5%	93.1%	99.5%	93.1%	93.1%	93.1%	93.1%	93.1%	30.1%
67. UDLA 257 ECUADOR	100%	100%	100%		100%	100%	100%	100%	100%	100%	99.5%	99.5%	99.5%	99.5%	99.0%	99.5%	93.1%	99.5%	93.1%	93.1%	93.1%	93.1%	93.1%	30.1%
68. UDLA 247 ECUADOR	100%	100%	100%	100%		100%	100%	100%	100%	100%	99.5%	99.5%	99.5%	99.5%	99.0%	99.5%	93.1%	99.5%	93.1%	93.1%	93.1%	93.1%	93.1%	30.1%
69. 267 ECUADOR	100%	100%	100%	100%	100%		100%	100%	100%	100%	99.5%	99.5%	99.5%	99.5%	99.0%	99.5%	93.1%	99.5%	93.1%	93.1%	93.1%	93.1%	93.1%	30.1%
70. UDLA 280 ECUADOR	100%	100%	100%	100%	100%	100%		100%	100%	100%	99.5%	99.5%	99.5%	99.5%	99.0%	99.5%	93.1%	99.5%	93.1%	93.1%	93.1%	93.1%	93.1%	30.1%
71. UDLA 299 ECUADOR	100%	100%	100%	100%	100%	100%	100%		100%	100%	99.5%	99.5%	99.5%	99.5%	99.0%	99.5%	93.1%	99.5%	93.1%	93.1%	93.1%	93.1%	93.1%	30.1%
72. UDLA 86 ECUADOR	100%	100%	100%	100%	100%	100%	100%	100%		100%	99.5%	99.5%	99.5%	99.5%	99.0%	99.5%	93.1%	99.5%	93.1%	93.1%	93.1%	93.1%	93.1%	30.1%
73. UDLA 203 ECUADOR	100%	100%	100%	100%	100%	100%	100%	100%	100%		99.5%	99.5%	99.5%	99.5%	99.0%	99.5%	93.1%	99.5%	93.1%	93.1%	93.1%	93.1%	93.1%	30.1%
74. ABD83863.1 | DQ32...	99.5%	99.5%	99.5%	99.5%	99.5%	99.5%	99.5%	99.5%	99.5%	99.5%		100%	100%	100%	99.5%	100%	93.6%	100%	93.6%	93.6%	93.6%	93.6%	93.6%	30.1%
75. AOS51012.1 |KU746...	99.5%	99.5%	99.5%	99.5%	99.5%	99.5%	99.5%	99.5%	99.5%	99.5%	100%		100%	100%	99.5%	100%	93.6%	100%	93.6%	93.6%	93.6%	93.6%	93.6%	30.1%
76. AKR76197.1 |KP231...	99.5%	99.5%	99.5%	99.5%	99.5%	99.5%	99.5%	99.5%	99.5%	99.5%	100%	100%		100%	99.5%	100%	93.6%	100%	93.6%	93.6%	93.6%	93.6%	93.6%	30.1%
77. ASU91621.1 | MF16...	99.5%	99.5%	99.5%	99.5%	99.5%	99.5%	99.5%	99.5%	99.5%	99.5%	100%	100%	100%		99.5%	100%	93.6%	100%	93.6%	93.6%	93.6%	93.6%	93.6%	30.1%
78. QIM09460.1 |MN42...	99.0%	99.0%	99.0%	99.0%	99.0%	99.0%	99.0%	99.0%	99.0%	99.0%	99.5%	99.5%	99.5%	99.5%		99.5%	93.1%	99.5%	93.1%	93.1%	93.1%	93.1%	93.1%	30.1%
79. QFR45466.1 |MK757...	99.5%	99.5%	99.5%	99.5%	99.5%	99.5%	99.5%	99.5%	99.5%	99.5%	100%	100%	100%	100%	99.5%		93.6%	100%	93.6%	93.6%	93.6%	93.6%	93.6%	30.1%
81. UDLA 50 ECUADOR	93.1%	93.1%	93.1%	93.1%	93.1%	93.1%	93.1%	93.1%	93.1%	93.1%	93.6%	93.6%	93.6%	93.6%	93.1%	93.6%		93.6%	100%	100%	100%	100%	100%	30.1%
82. AQX17377.1 | MT10...	99.5%	99.5%	99.5%	99.5%	99.5%	99.5%	99.5%	99.5%	99.5%	99.5%	100%	100%	100%	100%	99.5%	100%	93.6%		93.6%	93.6%	93.6%	93.6%	93.6%	30.1%
83. UDLA 337 ECUADOR	93.1%	93.1%	93.1%	93.1%	93.1%	93.1%	93.1%	93.1%	93.1%	93.1%	93.6%	93.6%	93.6%	93.6%	93.1%	93.6%	100%	93.6%		100%	100%	100%	100%	30.1%
84. UDLA 64 ECUADOR	93.1%	93.1%	93.1%	93.1%	93.1%	93.1%	93.1%	93.1%	93.1%	93.1%	93.6%	93.6%	93.6%	93.6%	93.1%	93.6%	100%	93.6%	100%		100%	100%	100%	30.1%
85. UDLA 56 ECUADOR	93.1%	93.1%	93.1%	93.1%	93.1%	93.1%	93.1%	93.1%	93.1%	93.1%	93.6%	93.6%	93.6%	93.6%	93.1%	93.6%	100%	93.6%	100%	100%		100%	100%	30.1%
86. UDLA 44 ECUADOR	93.1%	93.1%	93.1%	93.1%	93.1%	93.1%	93.1%	93.1%	93.1%	93.1%	93.6%	93.6%	93.6%	93.6%	93.1%	93.6%	100%	93.6%	100%	100%	100%		100%	30.1%
87. ANJ02344.1 | KT862...	93.1%	93.1%	93.1%	93.1%	93.1%	93.1%	93.1%	93.1%	93.1%	93.1%	93.6%	93.6%	93.6%	93.6%	93.1%	93.6%	100%	93.6%	100%	100%	100%	100%		30.1%
89. NP NC_001813.1 D...	30.1%	30.1%	30.1%	30.1%	30.1%	30.1%	30.1%	30.1%	30.1%	30.1%	30.1%	30.1%	30.1%	30.1%	30.1%	30.1%	30.1%	30.1%	30.1%	30.1%	30.1%	30.1%	30.1%	
