# Supplementary material for: Detection, Quantification and Molecular Characterization of Fowl Adenoviruses Circulating in Ecuadorian Chicken Flocks during 2019–2021
Source: Vet Sci. 2023 Feb 3;10(2):115. doi: 10.3390/vetsci10020115 (PMC9963715; doi:10.3390/vetsci10020115)
Supplement: Supplementary file 1 [file vetsci-10-00115-s001.zip › Table S2.pdf]

Table S2. Molecular Characterization of FAdV in Ecuadorian Chicken Flocks.

Sample		Age		Lines	FAdV	Origin	Clinical Signs
UDLA	44	44	Days	LH	**D2**	EC-H	RSS
UDLA	50	45	Weeks	BH	**D2**	EC-P	RSS
UDLA	56	30	Weeks	LH	**D2**	EC-T	RSS
UDLA	64	28	Days	BC	**D2**	EC-P	RSS
UDLA	337	4	Days	BC	**D2**	EC-P	RSS
UDLA	2	12	Days	BC	**D3**	EC-P	RSS
UDLA	3	35	Weeks	LH	**D3**	EC-I	RSS
UDLA	72	40	Weeks	LH	**D3**	EC-T	RSS
UDLA	76	26	Days	BC	**D3**	EC-P	RSS
UDLA	78	28	Days	BC	**D3**	EC-P	RSS
UDLA	80	30	Days	BC	**D3**	EC-P	RSS
UDLA	85	35	Days	BC	**D3**	EC-P	RSS
UDLA	86	36	Days	BC	**D3**	EC-P	RSS
UDLA	86-2	36	Days	BC	**D3**	EC-P	RSS
UDLA	88	25	Days	BC	**D3**	EC-P	RSS
UDLA	203	42	Days	BC	**D3**	EC-P	RSS
UDLA	207	42	Days	BC	**D3**	EC-P	RSS
UDLA	210	42	Days	BC	**D3**	EC-P	RSS
UDLA	213	42	Days	BC	**D3**	EC-P	RSS
UDLA	222	42	Days	BC	**D3**	EC-P	RSS
UDLA	226	42	Days	BC	**D3**	EC-P	RSS
UDLA	247	42	Days	BC	**D3**	EC-P	RSS
UDLA	250	42	Days	BC	**D3**	EC-P	RSS
UDLA	257	42	Days	BC	**D3**	EC-P	RSS
UDLA	267	42	Days	BC	**D3**	EC-P	RSS
UDLA	268	42	Days	BC	**D3**	EC-P	RSS
UDLA	276	42	Days	BC	**D3**	EC-P	RSS
UDLA	280	42	Days	BC	**D3**	EC-P	RSS
UDLA	299	42	Days	BC	**D3**	EC-P	RSS
UDLA	76F	26	Days	BC	**D3**	EC-P	RSS
UDLA	71	14	Days	BC	**E1**	EC-T	RSS
UDLA	4	12	Days	BC	**E2**	EC-P	RSS
UDLA	17	40	Weeks	LH	**E2**	EC-I	RSS
UDLA	282	42	Days	BC	**E2**	EC-P	RSS
UDLA	291	42	Days	BC	**E2**	EC-P	RSS
UDLA	292	42	Days	BC	**E2**	EC-P	RSS
UDLA	292-2	42	Days	BC	**E2**	EC-P	RSS
UDLA	303	42	Days	BC	**E2**	EC-P	RSS
UDLA	310	42	Days	BC	**E2**	EC-P	RSS
UDLA	310-2	42	Days	BC	**E2**	EC-P	RSS
UDLA	316	43	Days	BC	**E2**	EC-I	RSS
