# Supplementary material for: Detection, Quantification and Molecular Characterization of Fowl Adenoviruses Circulating in Ecuadorian Chicken Flocks during 2019–2021
Source: Vet Sci. 2023 Feb 3;10(2):115. doi: 10.3390/vetsci10020115 (PMC9963715; doi:10.3390/vetsci10020115)
Supplement: Supplementary file 1 [file vetsci-10-00115-s001.zip › Table S3.pdf]

Table S3. Comparison of the nucleotide identities of the sequences of Ecuadorian isolates of FAdV with other sequences of this virus

	1. NC 0...	2. GU18...	3. KY63...	4. KX53...	5. KU55...	6. KU56...	7. MH4...	8. MN7...	9. HE60...	10. MN...	11. NC ...	12. U46...	13. KX2...	14. NC ...	15. KC4...	16. MG...	17. UDL...	18. MF1...	19. KT8...	20. NC ...	21. NC ...	22. GU7...	23. KX2...	24. MK8...	25. KY2...	26. MT1...	27. KT8...	28. KT8...	29. KP8...	30. UDL...
1. NC 015323.1 CANADA		100%	98.7%	98.7%	98.7%	98.7%	98.7%	98.7%	99.4%	97.4%	74.1%	74.1%	74.1%	71.6%	71.6%	71.6%	64.6%	63.7%	72.9%	72.9%	73.8%	73.8%	73.7%	52.9%	62.1%	64.1%	74.1%	73.5%	55.9%	64.7%
2. GU188428.1 CANADA	100%		98.7%	98.7%	98.7%	98.7%	98.7%	98.7%	99.4%	97.4%	74.1%	74.1%	74.1%	71.6%	71.6%	71.6%	64.6%	63.7%	72.9%	72.9%	73.8%	73.8%	73.7%	52.9%	62.1%	64.1%	74.1%	73.5%	55.9%	64.7%
3. KY636400.1 CHINA	98.7%	98.7%		100%	100%	100%	100%	100%	98.9%	97.4%	74.1%	74.1%	74.2%	71.5%	71.5%	71.6%	64.7%	63.8%	73.0%	73.0%	73.7%	73.7%	73.7%	52.5%	61.8%	64.0%	74.0%	73.6%	55.9%	65.1%
4. KX538980.1 CHINA	98.7%	98.7%	100%		100%	100%	100%	100%	98.9%	97.4%	76.5%	76.5%	76.5%	73.6%	73.6%	73.7%	72.6%	71.8%	75.4%	75.4%	76.6%	76.6%	76.5%	58.1%	68.1%	70.6%	76.9%	76.1%	64.4%	71.2%
5. KU558761.1 CHINA	98.7%	98.7%	100%	100%		100%	100%	100%	98.9%	97.4%	74.1%	74.1%	74.2%	71.5%	71.5%	71.6%	64.7%	63.8%	73.0%	73.0%	73.7%	73.7%	73.7%	52.5%	61.8%	64.0%	74.0%	73.6%	55.9%	65.1%
6. KU569296.1 CHINA	98.7%	98.7%	100%	100%	100%		100%	100%	98.9%	97.4%	74.1%	74.1%	74.2%	71.5%	71.5%	71.6%	64.7%	63.8%	73.0%	73.0%	73.7%	73.7%	73.7%	52.5%	61.8%	64.0%	74.0%	73.6%	55.9%	65.1%
7. MH454598.1 CHINA	98.7%	98.7%	100%	100%	100%	100%		100%	98.9%	97.4%	74.1%	74.1%	74.2%	71.5%	71.5%	71.6%	64.7%	63.8%	73.0%	73.0%	73.7%	73.7%	73.7%	52.5%	61.8%	64.0%	74.0%	73.6%	55.9%	65.1%
8. MN781666.1 CHINA	98.7%	98.7%	100%	100%	100%	100%	100%		98.9%	97.4%	74.1%	74.1%	74.2%	71.5%	71.5%	71.6%	64.7%	63.8%	73.0%	73.0%	73.7%	73.7%	73.7%	52.5%	61.8%	64.0%	74.0%	73.6%	55.9%	65.1%
9. HE608152.1 AUSTRIA	99.4%	99.4%	98.9%	98.9%	98.9%	98.9%	98.9%	98.9%		97.7%	74.1%	74.1%	74.1%	71.5%	71.5%	71.5%	64.6%	63.7%	73.0%	73.0%	73.9%	73.9%	73.8%	52.9%	62.1%	64.2%	74.1%	73.5%	55.9%	64.6%
10. MN542422.1 CHINA	97.4%	97.4%	97.4%	97.4%	97.4%	97.4%	97.4%	97.4%	97.7%		73.9%	73.9%	73.9%	71.4%	71.4%	71.8%	65.0%	64.0%	73.4%	73.4%	74.1%	74.1%	73.8%	52.7%	62.1%	63.8%	74.2%	73.7%	55.4%	65.1%
11. NC 001720.1 AUSTR...	74.1%	74.1%	74.1%	76.5%	74.1%	74.1%	74.1%	74.1%	74.1%	73.9%		100%	99.7%	73.1%	73.1%	73.0%	68.5%	67.8%	75.5%	75.5%	76.2%	76.2%	75.5%	55.8%	66.0%	67.6%	76.1%	76.1%	59.8%	68.3%
12. U46933.1 AUSTRIA	74.1%	74.1%	74.1%	76.5%	74.1%	74.1%	74.1%	74.1%	74.1%	73.9%	100%		99.7%	73.1%	73.1%	73.0%	68.5%	67.8%	75.5%	75.5%	76.2%	76.2%	75.5%	55.8%	66.0%	67.6%	76.1%	76.1%	59.8%	68.3%
13. KX247012.1 POLAND	74.1%	74.1%	74.2%	76.5%	74.2%	74.2%	74.2%	74.2%	74.1%	73.9%	99.7%	99.7%		73.2%	73.2%	73.0%	68.5%	67.8%	75.5%	75.5%	76.2%	76.2%	75.6%	56.0%	66.1%	67.6%	76.1%	76.2%	60.1%	68.4%
14. NC 021221.1 NORT...	71.6%	71.6%	71.5%	73.6%	71.5%	71.5%	71.5%	71.5%	71.5%	71.4%	73.1%	73.1%	73.2%		100%	97.2%	73.4%	72.7%	77.9%	77.9%	77.1%	77.1%	76.8%	60.2%	68.8%	71.0%	77.1%	77.5%	64.8%	72.9%
15. KC493646.1 NORTH...	71.6%	71.6%	71.5%	73.6%	71.5%	71.5%	71.5%	71.5%	71.5%	71.4%	73.1%	73.1%	73.2%	100%		97.2%	73.4%	72.7%	77.9%	77.9%	77.1%	77.1%	76.8%	60.2%	68.8%	71.0%	77.1%	77.5%	64.8%	72.9%
16. MG953201.1 HUNG...	71.6%	71.6%	71.6%	73.7%	71.6%	71.6%	71.6%	71.6%	71.5%	71.8%	73.0%	73.0%	73.0%	97.2%	97.2%		73.1%	72.5%	77.8%	77.8%	77.3%	77.3%	77.0%	60.8%	69.1%	71.1%	77.3%	77.6%	65.2%	73.1%
17. UDLA 71 ECUADOR 6	64.6%	64.6%	64.7%	72.6%	64.7%	64.7%	64.7%	64.7%	64.6%	65.0%	68.5%	68.5%	68.5%	73.4%	73.4%	73.1%		99.9%	99.5%	99.5%	84.4%	84.4%	85.5%	76.1%	83.9%	85.1%	85.0%	88.1%	83.5%	86.7%
18. MF161433.1 ECUAD...	63.7%	63.7%	63.8%	71.8%	63.8%	63.8%	63.8%	63.8%	63.7%	64.0%	67.8%	67.8%	67.8%	72.7%	72.7%	72.5%	99.9%		99.4%	99.4%	83.8%	83.8%	84.9%	75.9%	83.7%	84.8%	84.4%	87.9%	83.7%	86.1%
19. KT862808.1 AUSTRIA	72.9%	72.9%	73.0%	75.4%	73.0%	73.0%	73.0%	73.0%	73.0%	73.4%	75.5%	75.5%	75.5%	77.9%	77.9%	77.8%	99.5%	99.4%		100%	90.9%	90.9%	91.4%	76.1%	83.6%	85.4%	90.9%	92.5%	83.7%	86.6%
20. NC 038332.1 JAPAN	72.9%	72.9%	73.0%	75.4%	73.0%	73.0%	73.0%	73.0%	73.0%	73.4%	75.5%	75.5%	75.5%	77.9%	77.9%	77.8%	99.5%	99.4%	100%		90.9%	90.9%	91.4%	76.1%	83.6%	85.4%	90.9%	92.5%	83.7%	86.6%
21. NC 014969.1 CANA...	73.8%	73.8%	73.7%	76.6%	73.7%	73.7%	73.7%	73.7%	73.9%	74.1%	76.2%	76.2%	76.2%	77.1%	77.1%	77.3%	84.4%	83.8%	90.9%	90.9%		100%	97.5%	92.4%	99.1%	97.2%	99.2%	93.9%	85.9%	84.8%
22. GU734104.1 CANA...	73.8%	73.8%	73.7%	76.6%	73.7%	73.7%	73.7%	73.7%	73.9%	74.1%	76.2%	76.2%	76.2%	77.1%	77.1%	77.3%	84.4%	83.8%	90.9%	90.9%	100%		97.5%	92.4%	99.1%	97.2%	99.2%	93.9%	85.9%	84.8%
23. KX258422.1 PERU	73.7%	73.7%	73.7%	76.5%	73.7%	73.7%	73.7%	73.7%	73.8%	73.8%	75.5%	75.5%	75.6%	76.8%	76.8%	77.0%	85.5%	84.9%	91.4%	91.4%	97.5%	97.5%		95.3%	97.7%	98.3%	97.3%	94.1%	86.5%	85.1%
24. MK850433.1 IRAN	52.9%	52.9%	52.5%	58.1%	52.5%	52.5%	52.5%	52.5%	52.9%	52.7%	55.8%	55.8%	56.0%	60.2%	60.2%	60.8%	76.1%	75.9%	76.1%	76.1%	92.4%	92.4%	95.3%		92.2%	92.8%	92.4%	82.8%	85.2%	74.6%
25. KY229168.1 BRAZIL	62.1%	62.1%	61.8%	68.1%	61.8%	61.8%	61.8%	61.8%	62.1%	62.1%	66.0%	66.0%	66.1%	68.8%	68.8%	69.1%	83.9%	83.7%	83.6%	83.6%	99.1%	99.1%	97.7%	92.2%		97.5%	99.4%	89.5%	85.7%	83.7%
26. MT104454.1 INDON...	64.1%	64.1%	64.0%	70.6%	64.0%	64.0%	64.0%	64.0%	64.2%	63.8%	67.6%	67.6%	67.6%	71.0%	71.0%	71.1%	85.1%	84.8%	85.4%	85.4%	97.2%	97.2%	98.3%	92.8%	97.5%		97.7%	91.8%	87.7%	84.9%
27. KT862811.1 UNITE...	74.1%	74.1%	74.0%	76.9%	74.0%	74.0%	74.0%	74.0%	74.1%	74.2%	76.1%	76.1%	76.1%	77.1%	77.1%	77.3%	85.0%	84.4%	90.9%	90.9%	99.2%	99.2%	97.3%	92.4%	99.4%	97.7%		93.9%	86.2%	85.0%
28. KT862809.1 JAPAN	73.5%	73.5%	73.6%	76.1%	73.6%	73.6%	73.6%	73.6%	73.5%	73.7%	76.1%	76.1%	76.2%	77.5%	77.5%	77.6%	88.1%	87.9%	92.5%	92.5%	93.9%	93.9%	94.1%	82.8%	89.5%	91.8%	93.9%		97.2%	87.2%
29. KP879219.1 POLAND	55.9%	55.9%	55.9%	64.4%	55.9%	55.9%	55.9%	55.9%	55.9%	55.4%	59.8%	59.8%	60.1%	64.8%	64.8%	65.2%	83.5%	83.7%	83.7%	83.7%	85.9%	85.9%	86.5%	85.2%	85.7%	87.7%	86.2%	97.2%		82.3%
30. UDLA 4 ECUADOR 8a	64.7%	64.7%	65.1%	71.2%	65.1%	65.1%	65.1%	65.1%	64.6%	65.1%	68.3%	68.3%	68.4%	72.9%	72.9%	73.1%	86.7%	86.1%	86.6%	86.6%	84.8%	84.8%	85.1%	74.6%	83.7%	84.9%	85.0%	87.2%	82.3%	
31. UDLA 17 ECUADOR	64.6%	64.6%	65.0%	71.3%	65.0%	65.0%	65.0%	65.0%	64.5%	65.0%	68.1%	68.1%	68.3%	72.9%	72.9%	73.2%	86.6%	86.1%	86.5%	86.5%	84.7%	84.7%	85.1%	74.6%	83.7%	84.9%	85.0%	87.1%	82.3%	100%
32. MG008488.1 MORO...	53.9%	53.9%	54.7%	54.2%	54.7%	54.7%	54.7%	54.7%	53.9%	54.7%	56.3%	56.3%	56.7%	63.3%	63.3%	63.7%	78.7%	78.5%	78.9%	78.9%	75.4%	75.4%	75.6%	74.5%	75.0%	75.4%	75.6%	78.5%	80.3%	97.5%
33. KT781516.1 EGYPT	52.1%	52.1%	53.0%	51.7%	53.0%	53.0%	53.0%	53.0%	52.1%	52.8%	54.2%	54.2%	54.4%	61.3%	61.3%	61.7%	76.5%	76.3%	76.7%	76.7%	74.4%	74.4%	74.8%	72.7%	74.0%	74.2%	74.6%	76.3%	79.3%	94.7%
34. UDLA 310-2 ECUAD...	64.1%	64.1%	64.5%	71.6%	64.5%	64.5%	64.5%	64.5%	64.0%	64.5%	67.1%	67.1%	67.2%	71.6%	71.6%	71.8%	85.3%	85.0%	85.3%	85.3%	83.4%	83.4%	83.7%	74.6%	82.7%	83.4%	83.7%	86.1%	82.3%	99.9%
35. UDLA 292-2 ECUAD...	64.1%	64.1%	64.5%	71.6%	64.5%	64.5%	64.5%	64.5%	64.0%	64.5%	67.1%	67.1%	67.2%	71.6%	71.6%	71.8%	85.3%	85.0%	85.3%	85.3%	83.4%	83.4%	83.7%	74.6%	82.7%	83.4%	83.7%	86.1%	82.3%	99.9%
36. UDLA 282 ECUADOR	65.1%	65.1%	65.5%	71.6%	65.5%	65.5%	65.5%	65.5%	65.0%	65.5%	68.5%	68.5%	68.6%	73.0%	73.0%	73.3%	86.8%	86.3%	86.9%	86.9%	85.0%	85.0%	85.5%	74.6%	83.9%	85.2%	85.4%	87.5%	82.3%	99.9%
37. UDLA 316 ECUADOR	65.1%	65.1%	65.5%	71.6%	65.5%	65.5%	65.5%	65.5%	65.0%	65.5%	68.5%	68.5%	68.6%	73.0%	73.0%	73.3%	86.8%	86.3%	86.9%	86.9%	85.0%	85.0%	85.5%	74.6%	83.9%	85.2%	85.4%	87.5%	82.3%	99.9%
38. UDLA 310 ECUADOR	65.1%	65.1%	65.5%	71.6%	65.5%	65.5%	65.5%	65.5%	65.0%	65.5%	68.5%	68.5%	68.6%	73.0%	73.0%	73.3%	86.8%	86.3%	86.9%	86.9%	85.0%	85.0%	85.5%	74.6%	83.9%	85.2%	85.4%	87.5%	82.3%	99.9%
39. UDLA 291 ECUADOR	65.1%	65.1%	65.5%	71.6%	65.5%	65.5%	65.5%	65.5%	65.0%	65.5%	68.5%	68.5%	68.6%	73.0%	73.0%	73.3%	86.8%	86.3%	86.9%	86.9%	85.0%	85.0%	85.5%	74.6%	83.9%	85.2%	85.4%	87.5%	82.3%	99.9%
40. UDLA 292 ECUADOR	65.1%	65.1%	65.5%	71.6%	65.5%	65.5%	65.5%	65.5%	65.0%	65.5%	68.5%	68.5%	68.6%	73.0%	73.0%	73.3%	86.8%	86.3%	86.9%	86.9%	85.0%	85.0%	85.5%	74.6%	83.9%	85.2%	85.4%	87.5%	82.3%	99.9%
41. UDLA 303 ECUADO...	65.1%	65.1%	65.5%	71.6%	65.5%	65.5%	65.5%	65.5%	65.0%	65.5%	68.5%	68.5%	68.6%	73.0%	73.0%	73.3%	86.8%	86.3%	86.9%	86.9%	85.0%	85.0%	85.5%	74.6%	83.9%	85.2%	85.4%	87.5%	82.3%	99.9%
42. MN453821.1 BRAZI...	66.8%	66.8%	67.1%	73.8%	67.1%	67.1%	67.1%	67.1%	66.7%	66.8%	69.5%	69.5%	69.7%	73.3%	73.3%	73.5%	86.6%	86.0%	87.4%	87.4%	86.0%	86.0%	86.7%	75.9%	84.2%	85.9%	86.4%	88.4%	82.4%	98.7%
43. KY229177.1 BRAZIL	66.4%	66.4%	66.7%	73.2%	66.7%	66.7%	66.7%	66.7%	66.3%	66.4%	69.3%	69.3%	69.4%	73.2%	73.2%	73.4%	86.6%	86.0%	87.3%	87.3%	85.9%	85.9%	86.6%	75.9%	84.2%	85.9%	86.3%	88.3%	82.4%	98.7%
44. DQ323985.1 USA	66.6%	66.6%	67.0%	73.4%	67.0%	67.0%	67.0%	67.0%	66.5%	66.7%	69.3%	69.3%	69.4%	73.7%	73.7%	73.8%	86.5%	85.9%	87.3%	87.3%	86.2%	86.2%	86.7%	75.4%	84.2%	85.7%	86.3%	87.9%	81.8%	99.0%
45. KT862810.1 JAPAN	73.9%	73.9%	74.1%	76.3%	74.1%	74.1%	74.1%	74.1%	74.0%	74.2%	76.2%	76.2%	76.3%	77.7%	77.7%	77.8%	86.0%	85.4%	91.6%	91.6%	93.1%	93.1%	92.2%	75.2%	84.0%	85.4%	93.3%	93.3%	81.5%	98.6%
46. NC 000899.1 POLA...	68.3%	68.3%	68.3%	69.7%	68.3%	68.3%	68.3%	68.3%	68.2%	68.5%	71.3%	71.3%	71.4%	73.7%	73.7%	74.2%	72.9%	72.6%	76.3%	76.3%	76.1%	76.1%	76.1%	63.8%	71.2%	72.8%	76.0%	75.9%	66.2%	70.9%
47. AF083975.2 CANADA	68.3%	68.3%	68.3%	69.7%	68.3%	68.3%	68.3%	68.3%	68.2%	68.5%	71.3%	71.3%	71.4%	73.7%	73.7%	74.2%	72.9%	72.6%	76.3%	76.3%	76.1%	76.1%	76.1%	63.8%	71.2%	72.8%	76.0%	75.9%	66.2%	70.9%
48. KT862807.1 JAPAN	68.4%	68.4%	68.5%	70.1%	68.5%	68.5%	68.5%	68.5%	68.4%	68.7%	71.5%	71.5%	71.5%	73.7%	73.7%	74.1%	73.3%	72.9%	76.4%	76.4%	76.0%	76.0%	75.9%	63.6%	70.8%	72.9%	75.9%	75.9%	66.6%	71.5%
49. UDLA 2 ECUADOR 11	62.1%	62.1%	62.3%	67.4%	62.3%	62.3%	62.3%	62.3%	61.9%	61.9%	65.8%	65.8%	65.8%	69.3%	69.3%	70.4%	73.9%	73.6%	74.0%	74.0%	71.5%	71.5%	71.7%	63.9%	71.1%	71.8%	71.5%	72.2%	66.9%	73.9%
50. UDLA 3 ECUADOR	62.1%	62.1%	62.3%	67.4%	62.3%	62.3%	62.3%	62.3%	61.9%	61.9%	65.8%	65.8%	65.8%	69.3%	69.3%	70.4%	74.0%	73.7%	74.1%	74.1%	71.7%	71.7%	71.8%	63.9%	71.2%	71.9%	71.7%	72.3%	66.9%	74.0%
51. UDLA 72 ECUADOR	62.1%	62.1%	62.3%	67.4%	62.3%	62.3%	62.3%	62.3%	61.9%	61.9%	65.8%	65.8%	65.8%	69.3%	69.3%	70.4%	74.0%	73.7%	74.1%	74.1%	71.7%	71.7%	71.8%	63.9%	71.2%	71.9%	71.7%	72.3%	66.9%	74.0%
52. UDLA 76 ECUADOR	62.1%	62.1%	62.3%	67.4%	62.3%	62.3%	62.3%	62.3%	61.9%	61.9%	65.8%	65.8%	65.8%	69.3%	69.3%	70.4%	73.9%	73.6%	74.0%	74.0%	71.5%	71.5%	71.7%	63.9%	71.1%	71.8%	71.5%	72.2%	66.9%	73.9%
53. UDLA 78 ECUADOR	62.1%	62.1%	62.3%	67.4%	62.3%	62.3%	62.3%	62.3%	61.9%	61.9%	65.8%	65.8%	65.8%	69.3%	69.3%	70.4%	73.9%	73.6%	74.0%	74.0%	71.5%	71.5%	71.7%	63.9%	71.1%	71.8%	71.5%	72.2%	66.9%	73.9%
54. UDLA 80 ECUADOR	62.1%	62.1%	62.3%	67.8%	62.3%	62.3%	62.3%	62.3%	61.9%	61.9%	65.6%	65.6%	65.6%	69.3%	69.3%	70.4%	74.0%	73.7%	74.1%	74.1%	71.7%	71.7%	71.8%	63.9%	71.1%	71.9%	71.7%	72.3%	66.9%	74.0%
55. UDLA 226 ECUADOR	62.8%	62.8%	62.9%	68.2%	62.9%	62.9%	62.9%	62.9%	62.5%	62.5%	66.8%	66.8%	66.8%	69.9%	69.9%	70.9%	74.4%	73.8%	74.6%	74.6%	72.3%	72.3%	72.4%	63.9%	71.1%	72.5%	72.3%	73.0%	66.9%	74.5%
56. UDLA 222 ECUADOR	62.8%	62.8%	62.9%	68.2%	62.9%	62.9%	62.9%	62.9%	62.5%	62.5%	66.8%	66.8%	66.8%	69.9%	69.9%	70.9%	74.4%	73.8%	74.6%	74.6%	72.3%	72.3%	72.4%	63.9%	71.1%	72.5%	72.3%	73.0%	66.9%	74.5%
57. UDLA 213 ECUADOR	62.8%	62.8%	62.9%	68.2%	62.9%	62.9%	62.9%	62.9%	62.5%	62.5%	66.8%	66.8%	66.8%	69.9%	69.9%	70.9%	74.4%	73.8%	74.6%	74.6%	72.3%	72.3%	72.4%	63.9%	71.1%	72.5%	72.3%	73.0%	66.9%	74.5%
58. UDLA 210 ECUADOR	62.8%	62.8%	62.9%	68.2%	62.9%	62.9%	62.9%	62.9%	62.5%	62.5%	66.8%	66.8%	66.8%	69.9%	69.9%	70.9%	74.4%	73.8%	74.6%	74.6%	72.3%	72.3%	72.4%	63.9%	71.1%	72.5%	72.3%	73.0%	66.9%	74.5%
59. UDLA 85 ECUADOR	62.1%	62.1%	62.4%	67.2%	62.4%	62.4%	62.4%	62.4%	62.0%	62.0%	65.9%	65.9%	65.9%	69.2%	69.2%	70.3%	74.0%	73.5%	74.1%	74.1%	71.7%	71.7%	71.8%	63.9%	71.1%	71.9%	71.7%	72.3%	66.9%	74.0%
60. UDLA 250 ECUADOR	62.6%	62.6%	62.7%	68.0%	62.7%	62.7%	62.7%	62.7%	62.4%	62.4%	66.5%	66.5%	66.5%	69.6%	69.6%	70.7%	74.4%	73.8%	74.5%	74.5%	72.1%	72.1%	72.3%	63.9%	71.1%	72.4%	72.1%	72.9%	66.9%	74.5%
61. UDLA 86-2 ECUADOR	61.3%	61.3%	61.6%	66.5%	61.6%	61.6%	61.6%	61.6%	61.2%	61.2%	64.5%	64.5%	64.5%	68.6%	68.6%	69.7%	73.8%	73.7%	73.9%	73.9%	71.2%	71.2%	71.3%	63.9%	71.1%	71.4%	71.2%	71.6%	66.9%	73.5%
62. UDLA 88 ECUADOR	61.9%	61.9%	62.2%	67.4%	62.2%	62.2%	62.2%	62.2%	61.8%	61.8%	65.7%	65.7%	65.7%	69.2%	69.2%	70.3%	73.9%	73.6%	74.0%	74.0%	71.5%	71.5%	71.7%	63.9%	71.1%	71.8%	71.5%	72.2%	66.9%	73.9%
63. UDLA 207 ECUADOR	61.9%	61.9%	62.2%	67.4%	62.2%	62.2%	62.2%	62.2%	61.8%	61.8%	65.7%	65.7%	65.7%	69.2%	69.2%	70.3%	73.9%	73.6%	74.0%	74.0%	71.5%	71.5%	71.7%	63.9%	71.1%	71.8%	71.5%	72.2%	66.9%	73.9%
64. UDLA 76F ECUADOR	61.9%	61.9%	62.2%	67.4%	62.2%	62.2%	62.2%	62.2%	61.8%	61.8%	65.7%	65.7%	65.7%	69.2%	69.2%	70.3%	73.9%	73.6%	74.0%	74.0%	71.5%	71.5%	71.7%	63.9%	71.1%	71.8%	71.5%	72.2%	66.9%	73.9%
65. UDLA 276 ECUADOR	63.0%	63.0%	63.1%	68.7%	63.1%	63.1%	63.1%	63.1%	62.7%	62.7%	66.7%	66.7%	66.7%	69.9%	69.9%	71.0%	74.5%	73.8%	74.7%	74.7%	72.3%	72.3%	72.5%	63.9%	71.1%	72.6%	72.3%	73.1%	66.9%	74.6%
66. UDLA 268 ECUADOR	63.0%	63.0%	63.1%	68.7%	63.1%	63.1%	63.1%	63.1%	62.7%	62.7%	66.7%	66.7%	66.7%	69.9%	69.9%	71.0%	74.5%	73.8%	74.7%	74.7%	72.3%	72.3%	72.5%	63.9%	71.1%	72.6%	72.3%	73.1%	66.9%	74.6%
67. UDLA 257 ECUADOR	62.2%	62.2%	62.3%	67.5%	62.3%	62.3%	62.3%	62.3%	61.9%	61.9%	65.8%	65.8%	65.8%	69.3%	69.3%	70.4%	74.0%	73.7%	74.1%	74.1%	71.8%	71.8%	71.8%	63.9%	71.1%	71.9%	71.8%	72.4%	66.9%	74.1%
68. UDLA 247 ECUADOR	62.7%	62.7%	62.8%	68.2%	62.8%	62.8%	62.8%	62.8%	62.5%	62.5%	66.5%	66.5%	66.5%	69.7%	69.7%	70.8%	74.4%	73.8%	74.5%	74.5%	72.1%	72.1%	72.3%	63.9%	71.1%	72.4%	72.1%	72.9%	66.9%	74.5%
69. UDLA 267 ECUADOR	63.0%	63.0%	63.1%	68.7%	63.1%	63.1%	63.1%	63.1%	62.7%	62.7%	66.7%	66.7%	66.7%	69.9%	69.9%	71.0%	74.5%	73.8%	74.7%	74.7%	72.3%	72.3%	72.5%	63.9%	71.1%	72.6%	72.3%	73.1%	66.9%	74.6%
70. UDLA 280 ECUADOR	63.0%	63.0%	63.1%	68.7%	63.1%	63.1%	63.1%	63.1%	62.7%	62.7%	66.7%	66.7%	66.7%	69.9%	69.9%	71.0%	74.5%	73.8%	74.7%	74.7%	72.3%	72.3%	72.5%	63.9%	71.1%	72.6%	72.3%	73.1%	66.9%	74.6%
71. UDLA 299 ECUADOR	63.0%	63.0%	63.1%	68.7%	63.1%	63.1%	63.1%	63.1%	62.7%	62.7%	66.7%	66.7%	66.7%	69.9%	69.9%	71.0%	74.5%	73.8%	74.7%	74.7%	72.3%	72.3%	72.5%	63.9%	71.1%	72.6%	72.3%	73.1%	66.9%	74.6%
72. UDLA 86 ECUADOR	63.0%	63.0%	63.1%	68.7%	63.1%	63.1%	63.1%	63.1%	62.7%	62.7%	66.7%	66.7%	66.7%	69.9%	69.9%	71.0%	74.5%	73.8%	74.7%	74.7%	72.3%	72.3%	72.5%	63.9%	71.1%	72.6%	72.3%	73.1%	66.9%	74.6%
73. UDLA 203 ECUADO...	62.7%	62.7%	62.8%	68.5%	62.8%	62.8%	62.8%	62.8%	62.5%	62.5%	66.5%	66.5%	66.5%	69.8%	69.8%	70.9%	74.4%	73.8%	74.5%	74.5%	72.1%	72.1%	72.3%	63.9%	71.1%	72.4%	72.1%	72.9%	66.9%	74.5%
74. DQ323984.1 USA 11	64.5%	64.5%	64.6%	70.2%	64.6%	64.6%	64.6%	64.6%	64.3%	64.5%	67.7%	67.7%	67.8%	70.3%	70.3%	71.4%	74.3%	73.5%	75.3%	75.3%	72.9%	72.9%	73.0%	63.9%	70.7%	72.4%	72.9%	73.7%	66.7%	74.1%
75. KU746335.1 MEXIC...	70.5%	70.5%	70.5%	72.2%	70.5%	70.5%	70.5%	70.5%	70.4%	70.7%	72.0%	72.0%	72.1%	75.5%	75.5%	75.9%	74.3%	73.5%	78.2%	78.2%	78.1%	78.1%	77.8%	63.9%	70.7%	72.4%	78.0%	78.0%	66.7%	74.1%
76. KP231537.1 CANADA	70.5%	70.5%	70.5%	72.2%	70.5%	70.5%	70.5%	70.5%	70.4%	70.7%	72.0%	72.0%	72.1%	75.5%	75.5%	75.9%	74.3%	73.5%	78.2%	78.2%	78.1%	78.1%	77.8%	63.9%	70.7%	72.4%	78.0%	78.0%	66.7%	74.1%
77. MF161434.1 ECUAD...	62.2%	62.2%	62.4%	67.9%	62.4%	62.4%	62.4%	62.4%	62.0%	62.0%	65.7%	65.7%	65.9%	69.1%	69.1%	70.2%	73.8%	73.5%	73.9%	73.9%	71.1%	71.1%	71.2%	63.9%	70.7%	71.3%	71.1%	72.0%	66.7%	73.7%
78. MN428137.1 PAKIS...	70.1%	70.1%	70.1%	71.7%	70.1%	70.1%	70.1%	70.1%	70.0%	70.3%	71.9%	71.9%	71.9%	75.4%	75.4%	75.9%	74.4%	73.6%	77.8%	77.8%	77.8%	77.8%	77.5%	63.9%	70.7%	72.6%	77.7%	77.8%	66.7%	74.5%
79. MK757569.1 IRAN	70.2%	70.2%	70.2%	71.8%	70.2%	70.2%	70.2%	70.2%	70.1%	70.4%	72.1%	72.1%	72.2%	75.5%	75.5%	75.9%	74.6%	73.9%	77.9%	77.9%	78.3%	78.3%	77.9%	63.9%	71.1%	73.1%	78.2%	78.2%	67.1%	74.7%
80. KY229175.1 BRAZIL	62.4%	62.4%	62.5%	68.2%	62.5%	62.5%	62.5%	62.5%	62.2%	62.2%	66.1%	66.1%	66.1%	69.2%	69.2%	70.3%	74.0%	73.9%	74.2%	74.2%	71.6%	71.6%	71.7%	63.9%	71.1%	71.8%	71.6%	72.4%	67.1%	74.2%
81. UDLA 50 ECUADOR 2	63.6%	63.6%	63.4%	67.5%	63.4%	63.4%	63.4%	63.4%	63.5%	63.5%	66.4%	66.4%	66.7%	70.2%	70.2%	70.9%	74.4%	73.6%	74.8%	74.8%	72.9%	72.9%	73.0%	65.0%	71.3%	72.9%	72.7%	73.4%	67.2%	74.4%
82. MT104458.1 INDON...	64.0%	64.0%	63.7%	68.0%	63.7%	63.7%	63.7%	63.7%	63.8%	63.8%	66.7%	66.7%	66.9%	70.4%	70.4%	71.1%	74.4%	73.6%	74.9%	74.9%	73.1%	73.1%	73.2%	65.0%	71.3%	73.0%	72.9%	73.5%	67.2%	74.4%
83. UDLA 337 ECUADOR	63.7%	63.7%	63.5%	67.5%	63.5%	63.5%	63.5%	63.5%	63.6%	63.6%	66.6%	66.6%	66.8%	70.5%	70.5%	71.1%	74.5%	73.8%	74.9%	74.9%	73.0%	73.0%	73.1%	65.0%	71.4%	73.0%	72.8%	73.5%	67.2%	74.5%
84. UDLA 64 ECUADOR	63.7%	63.7%	63.5%	67.5%	63.5%	63.5%	63.5%	63.5%	63.6%	63.6%	66.6%	66.6%	66.8%	70.5%	70.5%	71.1%	74.5%	73.8%	74.9%	74.9%	73.0%	73.0%	73.1%	65.0%	71.4%	73.0%	72.8%	73.5%	67.2%	74.5%
85. UDLA 56 ECUADOR	63.7%	63.7%	63.5%	67.5%	63.5%	63.5%	63.5%	63.5%	63.6%	63.6%	66.6%	66.6%	66.8%	70.5%	70.5%	71.1%	74.5%	73.8%	74.9%	74.9%	73.0%	73.0%	73.1%	65.0%	71.4%	73.0%	72.8%	73.5%	67.2%	74.5%
86. UDLA 44 ECUADOR 2	63.7%	63.7%	63.5%	67.5%	63.5%	63.5%	63.5%	63.5%	63.6%	63.6%	66.6%	66.6%	66.8%	70.5%	70.5%	71.1%	74.5%	73.8%	74.9%	74.9%	73.0%	73.0%	73.1%	65.0%	71.4%	73.0%	72.8%	73.5%	67.2%	74.5%
87. KT862805.1 UNITE...	70.5%	70.5%	70.3%	71.8%	70.3%	70.3%	70.3%	70.3%	70.3%	70.6%	72.0%	72.0%	72.1%	75.6%	75.6%	75.9%	74.4%	73.6%	78.2%	78.2%	78.4%	78.4%	78.1%	65.0%	71.3%	73.0%	78.2%	78.2%	67.2%	74.4%
88. MK468898.1 MORO...	59.9%	59.9%	60.0%	69.5%	60.0%	60.0%	60.0%	60.0%	59.6%	59.9%	63.4%	63.4%	63.3%	66.7%	66.7%	68.1%	71.5%	71.1%	71.7%	71.7%	68.4%	68.4%	68.9%	63.9%	67.5%	69.0%	68.7%	70.0%	66.7%	71.3%
89. DUCK ADENOVIRUS...	50.6%	50.6%	50.7%	51.6%	50.7%	50.7%	50.7%	50.7%	50.6%	50.9%	51.5%	51.5%	51.7%	51.3%	51.3%	51.0%	48.5%	47.9%	50.9%	50.9%	52.2%	52.2%	51.7%	43.8%	49.2%	49.1%	51.9%	51.5%	44.3%	48.9%

	31. UDL...	32. MG...	33. KT7...	34. UDL...	35. UDL...	36. UDL...	37. UDL...	38. UDL...	39. UDL...	40. UDL...	41. UDL...	42. MN...	43. KY2...	44. DQ3...	45. KT8...	46. NC ...	47. AF0...	48. KT8...	49. UDL...	50. UDL...	51. UDL...	52. UDL...	53. UDL...	54. UDL...	55. UDL...	56. UDL...	57. UDL...	58. UDL...	59. UDL...	60. UDL...
1. NC 015323.1 CANADA	64.6%	53.9%	52.1%	64.1%	64.1%	65.1%	65.1%	65.1%	65.1%	65.1%	65.1%	66.8%	66.4%	66.6%	73.9%	68.3%	68.3%	68.4%	62.1%	62.1%	62.1%	62.1%	62.1%	62.1%	62.8%	62.8%	62.8%	62.8%	62.1%	62.6%
2. GU188428.1 CANADA	64.6%	53.9%	52.1%	64.1%	64.1%	65.1%	65.1%	65.1%	65.1%	65.1%	65.1%	66.8%	66.4%	66.6%	73.9%	68.3%	68.3%	68.4%	62.1%	62.1%	62.1%	62.1%	62.1%	62.1%	62.8%	62.8%	62.8%	62.8%	62.1%	62.6%
3. KY636400.1 CHINA	65.0%	54.7%	53.0%	64.5%	64.5%	65.5%	65.5%	65.5%	65.5%	65.5%	65.5%	67.1%	66.7%	67.0%	74.1%	68.3%	68.3%	68.5%	62.3%	62.3%	62.3%	62.3%	62.3%	62.3%	62.9%	62.9%	62.9%	62.9%	62.4%	62.7%
4. KX538980.1 CHINA	71.3%	54.2%	51.7%	71.6%	71.6%	71.6%	71.6%	71.6%	71.6%	71.6%	71.6%	73.8%	73.2%	73.4%	76.3%	69.7%	69.7%	70.1%	67.4%	67.4%	67.4%	67.4%	67.4%	67.8%	68.2%	68.2%	68.2%	68.2%	67.2%	68.0%
5. KU558761.1 CHINA	65.0%	54.7%	53.0%	64.5%	64.5%	65.5%	65.5%	65.5%	65.5%	65.5%	65.5%	67.1%	66.7%	67.0%	74.1%	68.3%	68.3%	68.5%	62.3%	62.3%	62.3%	62.3%	62.3%	62.3%	62.9%	62.9%	62.9%	62.9%	62.4%	62.7%
6. KU569296.1 CHINA	65.0%	54.7%	53.0%	64.5%	64.5%	65.5%	65.5%	65.5%	65.5%	65.5%	65.5%	67.1%	66.7%	67.0%	74.1%	68.3%	68.3%	68.5%	62.3%	62.3%	62.3%	62.3%	62.3%	62.3%	62.9%	62.9%	62.9%	62.9%	62.4%	62.7%
7. MH454598.1 CHINA	65.0%	54.7%	53.0%	64.5%	64.5%	65.5%	65.5%	65.5%	65.5%	65.5%	65.5%	67.1%	66.7%	67.0%	74.1%	68.3%	68.3%	68.5%	62.3%	62.3%	62.3%	62.3%	62.3%	62.3%	62.9%	62.9%	62.9%	62.9%	62.4%	62.7%
8. MN781666.1 CHINA	65.0%	54.7%	53.0%	64.5%	64.5%	65.5%	65.5%	65.5%	65.5%	65.5%	65.5%	67.1%	66.7%	67.0%	74.1%	68.3%	68.3%	68.5%	62.3%	62.3%	62.3%	62.3%	62.3%	62.3%	62.9%	62.9%	62.9%	62.9%	62.4%	62.7%
9. HE608152.1 AUSTRIA	64.5%	53.9%	52.1%	64.0%	64.0%	65.0%	65.0%	65.0%	65.0%	65.0%	65.0%	66.7%	66.3%	66.5%	74.0%	68.2%	68.2%	68.4%	61.9%	61.9%	61.9%	61.9%	61.9%	61.9%	62.5%	62.5%	62.5%	62.5%	62.0%	62.4%
10. MN542422.1 CHINA	65.0%	54.7%	52.8%	64.5%	64.5%	65.5%	65.5%	65.5%	65.5%	65.5%	65.5%	66.8%	66.4%	66.7%	74.2%	68.5%	68.5%	68.7%	61.9%	61.9%	61.9%	61.9%	61.9%	61.9%	62.5%	62.5%	62.5%	62.5%	62.0%	62.4%
11. NC 001720.1 AUSTR...	68.1%	56.3%	54.2%	67.1%	67.1%	68.5%	68.5%	68.5%	68.5%	68.5%	68.5%	69.5%	69.3%	69.3%	76.2%	71.3%	71.3%	71.5%	65.8%	65.8%	65.8%	65.8%	65.8%	65.6%	66.8%	66.8%	66.8%	66.8%	65.9%	66.5%
12. U46933.1 AUSTRIA	68.1%	56.3%	54.2%	67.1%	67.1%	68.5%	68.5%	68.5%	68.5%	68.5%	68.5%	69.5%	69.3%	69.3%	76.2%	71.3%	71.3%	71.5%	65.8%	65.8%	65.8%	65.8%	65.8%	65.6%	66.8%	66.8%	66.8%	66.8%	65.9%	66.5%
13. KX247012.1 POLAND	68.3%	56.7%	54.4%	67.2%	67.2%	68.6%	68.6%	68.6%	68.6%	68.6%	68.6%	69.7%	69.4%	69.4%	76.3%	71.4%	71.4%	71.5%	65.8%	65.8%	65.8%	65.8%	65.8%	65.6%	66.8%	66.8%	66.8%	66.8%	65.9%	66.5%
14. NC 021221.1 NORT...	72.9%	63.3%	61.3%	71.6%	71.6%	73.0%	73.0%	73.0%	73.0%	73.0%	73.0%	73.3%	73.2%	73.7%	77.7%	73.7%	73.7%	73.7%	69.3%	69.3%	69.3%	69.3%	69.3%	69.3%	69.9%	69.9%	69.9%	69.9%	69.2%	69.6%
15. KC493646.1 NORTH...	72.9%	63.3%	61.3%	71.6%	71.6%	73.0%	73.0%	73.0%	73.0%	73.0%	73.0%	73.3%	73.2%	73.7%	77.7%	73.7%	73.7%	73.7%	69.3%	69.3%	69.3%	69.3%	69.3%	69.3%	69.9%	69.9%	69.9%	69.9%	69.2%	69.6%
16. MG953201.1 HUNG...	73.2%	63.7%	61.7%	71.8%	71.8%	73.3%	73.3%	73.3%	73.3%	73.3%	73.3%	73.5%	73.4%	73.8%	77.8%	74.2%	74.2%	74.1%	70.4%	70.4%	70.4%	70.4%	70.4%	70.4%	70.9%	70.9%	70.9%	70.9%	70.3%	70.7%
17. UDLA 71 ECUADOR 6	86.6%	78.7%	76.5%	85.3%	85.3%	86.8%	86.8%	86.8%	86.8%	86.8%	86.8%	86.6%	86.6%	86.5%	86.0%	72.9%	72.9%	73.3%	73.9%	74.0%	74.0%	73.9%	73.9%	74.0%	74.4%	74.4%	74.4%	74.4%	74.0%	74.4%
18. MF161433.1 ECUAD...	86.1%	78.5%	76.3%	85.0%	85.0%	86.3%	86.3%	86.3%	86.3%	86.3%	86.3%	86.0%	86.0%	85.9%	85.4%	72.6%	72.6%	72.9%	73.6%	73.7%	73.7%	73.6%	73.6%	73.7%	73.8%	73.8%	73.8%	73.8%	73.5%	73.8%
19. KT862808.1 AUSTRIA	86.5%	78.9%	76.7%	85.3%	85.3%	86.9%	86.9%	86.9%	86.9%	86.9%	86.9%	87.4%	87.3%	87.3%	91.6%	76.3%	76.3%	76.4%	74.0%	74.1%	74.1%	74.0%	74.0%	74.1%	74.6%	74.6%	74.6%	74.6%	74.1%	74.5%
20. NC 038332.1 JAPAN	86.5%	78.9%	76.7%	85.3%	85.3%	86.9%	86.9%	86.9%	86.9%	86.9%	86.9%	87.4%	87.3%	87.3%	91.6%	76.3%	76.3%	76.4%	74.0%	74.1%	74.1%	74.0%	74.0%	74.1%	74.6%	74.6%	74.6%	74.6%	74.1%	74.5%
21. NC 014969.1 CANA...	84.7%	75.4%	74.4%	83.4%	83.4%	85.0%	85.0%	85.0%	85.0%	85.0%	85.0%	86.0%	85.9%	86.2%	93.1%	76.1%	76.1%	76.0%	71.5%	71.7%	71.7%	71.5%	71.5%	71.7%	72.3%	72.3%	72.3%	72.3%	71.7%	72.1%
22. GU734104.1 CANA...	84.7%	75.4%	74.4%	83.4%	83.4%	85.0%	85.0%	85.0%	85.0%	85.0%	85.0%	86.0%	85.9%	86.2%	93.1%	76.1%	76.1%	76.0%	71.5%	71.7%	71.7%	71.5%	71.5%	71.7%	72.3%	72.3%	72.3%	72.3%	71.7%	72.1%
23. KX258422.1 PERU	85.1%	75.6%	74.8%	83.7%	83.7%	85.5%	85.5%	85.5%	85.5%	85.5%	85.5%	86.7%	86.6%	86.7%	92.2%	76.1%	76.1%	75.9%	71.7%	71.8%	71.8%	71.7%	71.7%	71.8%	72.4%	72.4%	72.4%	72.4%	71.8%	72.3%
24. MK850433.1 IRAN	74.6%	74.5%	72.7%	74.6%	74.6%	74.6%	74.6%	74.6%	74.6%	74.6%	74.6%	75.9%	75.9%	75.4%	75.2%	63.8%	63.8%	63.6%	63.9%	63.9%	63.9%	63.9%	63.9%	63.9%	63.9%	63.9%	63.9%	63.9%	63.9%	63.9%
25. KY229168.1 BRAZIL	83.7%	75.0%	74.0%	82.7%	82.7%	83.9%	83.9%	83.9%	83.9%	83.9%	83.9%	84.2%	84.2%	84.2%	84.0%	71.2%	71.2%	70.8%	71.1%	71.2%	71.2%	71.1%	71.1%	71.1%	71.1%	71.1%	71.1%	71.1%	71.1%	71.1%
26. MT104454.1 INDON...	84.9%	75.4%	74.2%	83.4%	83.4%	85.2%	85.2%	85.2%	85.2%	85.2%	85.2%	85.9%	85.9%	85.7%	85.4%	72.8%	72.8%	72.9%	71.8%	71.9%	71.9%	71.8%	71.8%	71.9%	72.5%	72.5%	72.5%	72.5%	71.9%	72.4%
27. KT862811.1 UNITE...	85.0%	75.6%	74.6%	83.7%	83.7%	85.4%	85.4%	85.4%	85.4%	85.4%	85.4%	86.4%	86.3%	86.3%	93.3%	76.0%	76.0%	75.9%	71.5%	71.7%	71.7%	71.5%	71.5%	71.7%	72.3%	72.3%	72.3%	72.3%	71.7%	72.1%
28. KT862809.1 JAPAN	87.1%	78.5%	76.3%	86.1%	86.1%	87.5%	87.5%	87.5%	87.5%	87.5%	87.5%	88.4%	88.3%	87.9%	93.3%	75.9%	75.9%	75.9%	72.2%	72.3%	72.3%	72.2%	72.2%	72.3%	73.0%	73.0%	73.0%	73.0%	72.3%	72.9%
29. KP879219.1 POLAND	82.3%	80.3%	79.3%	82.3%	82.3%	82.3%	82.3%	82.3%	82.3%	82.3%	82.3%	82.4%	82.4%	81.8%	81.5%	66.2%	66.2%	66.6%	66.9%	66.9%	66.9%	66.9%	66.9%	66.9%	66.9%	66.9%	66.9%	66.9%	66.9%	66.9%
30. UDLA 4 ECUADOR 8a	100%	97.5%	94.7%	99.9%	99.9%	99.9%	99.9%	99.9%	99.9%	99.9%	99.9%	98.7%	98.7%	99.0%	98.6%	70.9%	70.9%	71.5%	73.9%	74.0%	74.0%	73.9%	73.9%	74.0%	74.5%	74.5%	74.5%	74.5%	74.0%	74.5%
31. UDLA 17 ECUADOR		97.5%	94.7%	99.9%	99.9%	99.9%	99.9%	99.9%	99.9%	99.9%	99.9%	98.7%	98.7%	99.0%	98.6%	70.8%	70.8%	71.4%	73.9%	74.0%	74.0%	73.9%	73.9%	74.0%	74.4%	74.4%	74.4%	74.4%	73.9%	74.4%
32. MG008488.1 MORO...	97.5%		98.6%	97.5%	97.5%	97.5%	97.5%	97.5%	97.5%	97.5%	97.5%	95.9%	95.9%	96.5%	96.1%	62.3%	62.3%	63.4%	66.3%	66.3%	66.3%	66.3%	66.3%	66.3%	66.3%	66.3%	66.3%	66.3%	66.3%	66.3%
33. KT781516.1 EGYPT	94.7%	98.6%		94.7%	94.7%	94.7%	94.7%	94.7%	94.7%	94.7%	94.7%	95.0%	95.0%	95.6%	95.6%	60.9%	60.9%	62.2%	64.7%	64.7%	64.7%	64.7%	64.7%	64.7%	64.7%	64.7%	64.7%	64.7%	64.7%	64.7%
34. UDLA 310-2 ECUAD...	99.9%	97.5%	94.7%		100%	100%	100%	100%	100%	100%	100%	98.8%	98.8%	99.2%	98.8%	69.4%	69.4%	69.9%	72.6%	72.6%	72.6%	72.6%	72.6%	72.7%	73.1%	73.1%	73.1%	73.1%	72.5%	73.1%
35. UDLA 292-2 ECUAD...	99.9%	97.5%	94.7%	100%		100%	100%	100%	100%	100%	100%	98.8%	98.8%	99.2%	98.8%	69.4%	69.4%	69.9%	72.6%	72.6%	72.6%	72.6%	72.6%	72.7%	73.1%	73.1%	73.1%	73.1%	72.5%	73.1%
36. UDLA 282 ECUADOR	99.9%	97.5%	94.7%	100%	100%		100%	100%	100%	100%	100%	98.8%	98.8%	99.2%	98.7%	71.1%	71.1%	71.7%	73.9%	74.0%	74.0%	73.9%	73.9%	74.0%	74.6%	74.6%	74.6%	74.6%	74.0%	74.5%
37. UDLA 316 ECUADOR	99.9%	97.5%	94.7%	100%	100%	100%		100%	100%	100%	100%	98.8%	98.8%	99.2%	98.7%	71.1%	71.1%	71.7%	73.9%	74.0%	74.0%	73.9%	73.9%	74.0%	74.6%	74.6%	74.6%	74.6%	74.0%	74.5%
38. UDLA 310 ECUADOR	99.9%	97.5%	94.7%	100%	100%	100%	100%		100%	100%	100%	98.8%	98.8%	99.2%	98.7%	71.1%	71.1%	71.7%	73.9%	74.0%	74.0%	73.9%	73.9%	74.0%	74.6%	74.6%	74.6%	74.6%	74.0%	74.5%
39. UDLA 291 ECUADOR	99.9%	97.5%	94.7%	100%	100%	100%	100%	100%		100%	100%	98.8%	98.8%	99.2%	98.7%	71.1%	71.1%	71.7%	73.9%	74.0%	74.0%	73.9%	73.9%	74.0%	74.6%	74.6%	74.6%	74.6%	74.0%	74.5%
40. UDLA 292 ECUADOR	99.9%	97.5%	94.7%	100%	100%	100%	100%	100%	100%		100%	98.8%	98.8%	99.2%	98.7%	71.1%	71.1%	71.7%	73.9%	74.0%	74.0%	73.9%	73.9%	74.0%	74.6%	74.6%	74.6%	74.6%	74.0%	74.5%
41. UDLA 303 ECUADO...	99.9%	97.5%	94.7%	100%	100%	100%	100%	100%	100%	100%		98.8%	98.8%	99.2%	98.7%	71.1%	71.1%	71.7%	73.9%	74.0%	74.0%	73.9%	73.9%	74.0%	74.6%	74.6%	74.6%	74.6%	74.0%	74.5%
42. MN453821.1 BRAZI...	98.7%	95.9%	95.0%	98.8%	98.8%	98.8%	98.8%	98.8%	98.8%	98.8%	98.8%		100%	99.1%	99.1%	72.4%	72.4%	73.1%	73.1%	73.3%	73.3%	73.1%	73.1%	73.3%	73.9%	73.9%	73.9%	73.9%	73.3%	73.8%
43. KY229177.1 BRAZIL	98.7%	95.9%	95.0%	98.8%	98.8%	98.8%	98.8%	98.8%	98.8%	98.8%	98.8%	100%		99.2%	99.2%	72.0%	72.0%	72.6%	73.1%	73.3%	73.3%	73.1%	73.1%	73.3%	73.9%	73.9%	73.9%	73.9%	73.3%	73.8%
44. DQ323985.1 USA	99.0%	96.5%	95.6%	99.2%	99.2%	99.2%	99.2%	99.2%	99.2%	99.2%	99.2%	99.1%	99.2%		99.6%	72.3%	72.3%	73.0%	73.8%	73.9%	73.9%	73.8%	73.8%	73.9%	74.5%	74.5%	74.5%	74.5%	73.9%	74.4%
45. KT862810.1 JAPAN	98.6%	96.1%	95.6%	98.8%	98.8%	98.7%	98.7%	98.7%	98.7%	98.7%	98.7%	99.1%	99.2%	99.6%		75.7%	75.7%	75.8%	73.5%	73.6%	73.6%	73.5%	73.5%	73.6%	74.3%	74.3%	74.3%	74.3%	73.6%	74.2%
46. NC 000899.1 POLA...	70.8%	62.3%	60.9%	69.4%	69.4%	71.1%	71.1%	71.1%	71.1%	71.1%	71.1%	72.4%	72.0%	72.3%	75.7%		100%	97.6%	79.0%	79.1%	79.1%	79.0%	79.0%	78.9%	78.9%	78.9%	78.9%	78.9%	79.0%	78.8%
47. AF083975.2 CANADA	70.8%	62.3%	60.9%	69.4%	69.4%	71.1%	71.1%	71.1%	71.1%	71.1%	71.1%	72.4%	72.0%	72.3%	75.7%	100%		97.6%	79.0%	79.1%	79.1%	79.0%	79.0%	78.9%	78.9%	78.9%	78.9%	78.9%	79.0%	78.8%
48. KT862807.1 JAPAN	71.4%	63.4%	62.2%	69.9%	69.9%	71.7%	71.7%	71.7%	71.7%	71.7%	71.7%	73.1%	72.6%	73.0%	75.8%	97.6%	97.6%		78.6%	78.5%	78.5%	78.6%	78.6%	78.5%	78.5%	78.5%	78.5%	78.5%	78.6%	78.4%
49. UDLA 2 ECUADOR 11	73.9%	66.3%	64.7%	72.6%	72.6%	73.9%	73.9%	73.9%	73.9%	73.9%	73.9%	73.1%	73.1%	73.8%	73.5%	79.0%	79.0%	78.6%		99.9%	99.9%	100%	100%	99.8%	99.9%	99.9%	99.9%	99.9%	99.9%	99.9%
50. UDLA 3 ECUADOR	74.0%	66.3%	64.7%	72.6%	72.6%	74.0%	74.0%	74.0%	74.0%	74.0%	74.0%	73.3%	73.3%	73.9%	73.6%	79.1%	79.1%	78.5%	99.9%		100%	99.9%	99.9%	99.6%	99.8%	99.8%	99.8%	99.8%	99.8%	99.8%
51. UDLA 72 ECUADOR	74.0%	66.3%	64.7%	72.6%	72.6%	74.0%	74.0%	74.0%	74.0%	74.0%	74.0%	73.3%	73.3%	73.9%	73.6%	79.1%	79.1%	78.5%	99.9%	100%		99.9%	99.9%	99.6%	99.8%	99.8%	99.8%	99.8%	99.8%	99.8%
52. UDLA 76 ECUADOR	73.9%	66.3%	64.7%	72.6%	72.6%	73.9%	73.9%	73.9%	73.9%	73.9%	73.9%	73.1%	73.1%	73.8%	73.5%	79.0%	79.0%	78.6%	100%	99.9%	99.9%		100%	99.8%	99.9%	99.9%	99.9%	99.9%	99.9%	99.9%
53. UDLA 78 ECUADOR	73.9%	66.3%	64.7%	72.6%	72.6%	73.9%	73.9%	73.9%	73.9%	73.9%	73.9%	73.1%	73.1%	73.8%	73.5%	79.0%	79.0%	78.6%	100%	99.9%	99.9%	100%		99.8%	99.9%	99.9%	99.9%	99.9%	99.9%	99.9%
54. UDLA 80 ECUADOR	74.0%	66.3%	64.7%	72.7%	72.7%	74.0%	74.0%	74.0%	74.0%	74.0%	74.0%	73.3%	73.3%	73.9%	73.6%	78.9%	78.9%	78.5%	99.8%	99.6%	99.6%	99.8%	99.8%		99.9%	99.9%	99.9%	99.9%	99.9%	99.9%
55. UDLA 226 ECUADOR	74.4%	66.3%	64.7%	73.1%	73.1%	74.6%	74.6%	74.6%	74.6%	74.6%	74.6%	73.9%	73.9%	74.5%	74.3%	78.9%	78.9%	78.5%	99.9%	99.8%	99.8%	99.9%	99.9%	99.9%		100%	100%	100%	100%	100%
56. UDLA 222 ECUADOR	74.4%	66.3%	64.7%	73.1%	73.1%	74.6%	74.6%	74.6%	74.6%	74.6%	74.6%	73.9%	73.9%	74.5%	74.3%	78.9%	78.9%	78.5%	99.9%	99.8%	99.8%	99.9%	99.9%	99.9%	100%		100%	100%	100%	100%
57. UDLA 213 ECUADOR	74.4%	66.3%	64.7%	73.1%	73.1%	74.6%	74.6%	74.6%	74.6%	74.6%	74.6%	73.9%	73.9%	74.5%	74.3%	78.9%	78.9%	78.5%	99.9%	99.8%	99.8%	99.9%	99.9%	99.9%	100%	100%		100%	100%	100%
58. UDLA 210 ECUADOR	74.4%	66.3%	64.7%	73.1%	73.1%	74.6%	74.6%	74.6%	74.6%	74.6%	74.6%	73.9%	73.9%	74.5%	74.3%	78.9%	78.9%	78.5%	99.9%	99.8%	99.8%	99.9%	99.9%	99.9%	100%	100%	100%		100%	100%
59. UDLA 85 ECUADOR	74.0%	66.3%	64.7%	72.5%	72.5%	74.0%	74.0%	74.0%	74.0%	74.0%	74.0%	73.3%	73.3%	73.9%	73.6%	79.0%	79.0%	78.6%	99.9%	99.8%	99.8%	99.9%	99.9%	99.9%	100%	100%	100%	100%		100%
60. UDLA 250 ECUADOR	74.5%	66.3%	64.7%	73.1%	73.1%	74.5%	74.5%	74.5%	74.5%	74.5%	74.5%	73.8%	73.8%	74.4%	74.2%	78.8%	78.8%	78.4%	99.9%	99.8%	99.8%	99.9%	99.9%	99.9%	100%	100%	100%	100%	100%	
61. UDLA 86-2 ECUADOR	73.5%	66.3%	64.7%	72.3%	72.3%	73.5%	73.5%	73.5%	73.5%	73.5%	73.5%	72.7%	72.7%	73.2%	73.0%	78.5%	78.5%	78.0%	99.9%	99.7%	99.7%	99.9%	99.9%	100%	100%	100%	100%	100%	100%	100%
62. UDLA 88 ECUADOR	73.9%	66.3%	64.7%	72.6%	72.6%	73.9%	73.9%	73.9%	73.9%	73.9%	73.9%	73.1%	73.1%	73.8%	73.5%	78.9%	78.9%	78.5%	99.9%	99.8%	99.8%	99.9%	99.9%	99.9%	100%	100%	100%	100%	100%	100%
63. UDLA 207 ECUADOR	73.9%	66.3%	64.7%	72.6%	72.6%	73.9%	73.9%	73.9%	73.9%	73.9%	73.9%	73.1%	73.1%	73.8%	73.5%	78.9%	78.9%	78.5%	99.9%	99.8%	99.8%	99.9%	99.9%	99.9%	100%	100%	100%	100%	100%	100%
64. UDLA 76F ECUADOR	73.9%	66.3%	64.7%	72.6%	72.6%	73.9%	73.9%	73.9%	73.9%	73.9%	73.9%	73.1%	73.1%	73.8%	73.5%	78.9%	78.9%	78.5%	99.9%	99.8%	99.8%	99.9%	99.9%	99.9%	100%	100%	100%	100%	100%	100%
65. UDLA 276 ECUADOR	74.5%	66.3%	64.7%	73.3%	73.3%	74.7%	74.7%	74.7%	74.7%	74.7%	74.7%	74.0%	74.0%	74.6%	74.4%	78.8%	78.8%	78.5%	99.9%	99.8%	99.8%	99.9%	99.9%	99.9%	100%	100%	100%	100%	100%	100%
66. UDLA 268 ECUADOR	74.5%	66.3%	64.7%	73.3%	73.3%	74.7%	74.7%	74.7%	74.7%	74.7%	74.7%	74.0%	74.0%	74.6%	74.4%	78.8%	78.8%	78.5%	99.9%	99.8%	99.8%	99.9%	99.9%	99.9%	100%	100%	100%	100%	100%	100%
67. UDLA 257 ECUADOR	74.1%	66.3%	64.7%	72.8%	72.8%	74.1%	74.1%	74.1%	74.1%	74.1%	74.1%	73.4%	73.4%	74.0%	73.8%	78.6%	78.6%	78.2%	99.9%	99.8%	99.8%	99.9%	99.9%	99.9%	100%	100%	100%	100%	100%	100%
68. UDLA 247 ECUADOR	74.5%	66.3%	64.7%	73.1%	73.1%	74.5%	74.5%	74.5%	74.5%	74.5%	74.5%	73.8%	73.8%	74.4%	74.2%	78.7%	78.7%	78.3%	99.9%	99.8%	99.8%	99.9%	99.9%	99.9%	100%	100%	100%	100%	100%	100%
69. UDLA 267 ECUADOR	74.5%	66.3%	64.7%	73.3%	73.3%	74.7%	74.7%	74.7%	74.7%	74.7%	74.7%	74.0%	74.0%	74.6%	74.4%	78.8%	78.8%	78.5%	99.9%	99.8%	99.8%	99.9%	99.9%	99.9%	100%	100%	100%	100%	100%	100%
70. UDLA 280 ECUADOR	74.5%	66.3%	64.7%	73.3%	73.3%	74.7%	74.7%	74.7%	74.7%	74.7%	74.7%	74.0%	74.0%	74.6%	74.4%	78.8%	78.8%	78.5%	99.9%	99.8%	99.8%	99.9%	99.9%	99.9%	100%	100%	100%	100%	100%	100%
71. UDLA 299 ECUADOR	74.5%	66.3%	64.7%	73.3%	73.3%	74.7%	74.7%	74.7%	74.7%	74.7%	74.7%	74.0%	74.0%	74.6%	74.4%	78.8%	78.8%	78.5%	99.9%	99.8%	99.8%	99.9%	99.9%	99.9%	100%	100%	100%	100%	100%	100%
72. UDLA 86 ECUADOR	74.5%	66.3%	64.7%	73.3%	73.3%	74.7%	74.7%	74.7%	74.7%	74.7%	74.7%	74.0%	74.0%	74.6%	74.4%	78.8%	78.8%	78.5%	99.9%	99.8%	99.8%	99.9%	99.9%	99.9%	100%	100%	100%	100%	100%	100%
73. UDLA 203 ECUADO...	74.5%	66.3%	64.7%	73.2%	73.2%	74.5%	74.5%	74.5%	74.5%	74.5%	74.5%	73.8%	73.8%	74.4%	74.2%	78.7%	78.7%	78.3%	99.9%	99.8%	99.8%	99.9%	99.9%	99.9%	100%	100%	100%	100%	100%	100%
74. DQ323984.1 USA 11	74.0%	66.3%	64.7%	72.8%	72.8%	74.3%	74.3%	74.3%	74.3%	74.3%	74.3%	74.6%	74.2%	75.0%	74.8%	80.5%	80.5%	79.5%	98.6%	98.8%	98.8%	98.6%	98.6%	98.4%	98.6%	98.6%	98.6%	98.6%	98.5%	98.6%
75. KU746335.1 MEXIC...	74.0%	66.3%	64.7%	72.8%	72.8%	74.3%	74.3%	74.3%	74.3%	74.3%	74.3%	74.6%	74.2%	75.0%	78.7%	80.3%	80.3%	80.0%	98.6%	98.8%	98.8%	98.6%	98.6%	98.4%	98.6%	98.6%	98.6%	98.6%	98.5%	98.6%
76. KP231537.1 CANADA	74.0%	66.3%	64.7%	72.8%	72.8%	74.3%	74.3%	74.3%	74.3%	74.3%	74.3%	74.6%	74.2%	75.0%	78.7%	80.3%	80.3%	80.0%	98.6%	98.8%	98.8%	98.6%	98.6%	98.4%	98.6%	98.6%	98.6%	98.6%	98.5%	98.6%
77. MF161434.1 ECUAD...	73.7%	66.3%	64.7%	72.4%	72.4%	73.7%	73.7%	73.7%	73.7%	73.7%	73.7%	72.9%	72.9%	73.5%	73.3%	79.1%	79.1%	77.8%	98.6%	98.8%	98.8%	98.6%	98.6%	98.4%	98.5%	98.5%	98.5%	98.5%	98.5%	98.5%
78. MN428137.1 PAKIS...	74.4%	66.7%	65.1%	73.2%	73.2%	74.7%	74.7%	74.7%	74.7%	74.7%	74.7%	75.1%	74.7%	75.4%	78.6%	79.7%	79.7%	79.7%	99.3%	99.1%	99.1%	99.3%	99.3%	99.0%	99.0%	99.0%	99.0%	99.0%	99.1%	99.0%
79. MK757569.1 IRAN	74.6%	66.5%	64.9%	73.5%	73.5%	75.0%	75.0%	75.0%	75.0%	75.0%	75.0%	75.3%	74.9%	75.7%	79.0%	79.8%	79.8%	79.7%	99.9%	99.8%	99.8%	99.9%	99.9%	99.6%	99.8%	99.8%	99.8%	99.8%	99.8%	99.8%
80. KY229175.1 BRAZIL	74.2%	66.5%	64.9%	73.3%	73.3%	74.2%	74.2%	74.2%	74.2%	74.2%	74.2%	73.4%	73.4%	74.0%	73.8%	78.6%	78.6%	78.0%	99.9%	99.7%	99.7%	99.9%	99.9%	99.6%	99.8%	99.8%	99.8%	99.8%	99.7%	99.8%
81. UDLA 50 ECUADOR 2	74.3%	66.7%	64.9%	73.1%	73.1%	74.5%	74.5%	74.5%	74.5%	74.5%	74.5%	74.0%	74.0%	74.5%	74.3%	78.6%	78.6%	77.6%	94.8%	94.9%	94.9%	94.8%	94.8%	94.5%	94.9%	94.9%	94.9%	94.9%	94.7%	94.8%
82. MT104458.1 INDON...	74.3%	66.7%	64.9%	73.1%	73.1%	74.5%	74.5%	74.5%	74.5%	74.5%	74.5%	74.1%	74.1%	74.7%	74.5%	79.0%	79.0%	77.9%	94.8%	94.9%	94.9%	94.8%	94.8%	94.5%	94.9%	94.9%	94.9%	94.9%	94.7%	94.8%
83. UDLA 337 ECUADOR	74.4%	66.7%	64.9%	73.2%	73.2%	74.6%	74.6%	74.6%	74.6%	74.6%	74.6%	74.1%	74.1%	74.7%	74.4%	78.5%	78.5%	77.5%	94.8%	94.9%	94.9%	94.8%	94.8%	94.5%	94.9%	94.9%	94.9%	94.9%	94.7%	94.8%
84. UDLA 64 ECUADOR	74.4%	66.7%	64.9%	73.2%	73.2%	74.6%	74.6%	74.6%	74.6%	74.6%	74.6%	74.1%	74.1%	74.7%	74.4%	78.5%	78.5%	77.5%	94.8%	94.9%	94.9%	94.8%	94.8%	94.5%	94.9%	94.9%	94.9%	94.9%	94.7%	94.8%
85. UDLA 56 ECUADOR	74.4%	66.7%	64.9%	73.2%	73.2%	74.6%	74.6%	74.6%	74.6%	74.6%	74.6%	74.1%	74.1%	74.7%	74.4%	78.5%	78.5%	77.5%	94.8%	94.9%	94.9%	94.8%	94.8%	94.5%	94.9%	94.9%	94.9%	94.9%	94.7%	94.8%
86. UDLA 44 ECUADOR 2	74.4%	66.7%	64.9%	73.2%	73.2%	74.6%	74.6%	74.6%	74.6%	74.6%	74.6%	74.1%	74.1%	74.7%	74.4%	78.5%	78.5%	77.5%	94.8%	94.9%	94.9%	94.8%	94.8%	94.5%	94.9%	94.9%	94.9%	94.9%	94.7%	94.8%
87. KT862805.1 UNITE...	74.3%	66.7%	64.9%	73.1%	73.1%	74.5%	74.5%	74.5%	74.5%	74.5%	74.5%	74.9%	74.4%	75.2%	79.0%	80.2%	80.2%	79.8%	94.8%	94.9%	94.9%	94.8%	94.8%	94.5%	94.9%	94.9%	94.9%	94.9%	94.7%	94.8%
88. MK468898.1 MORO...	71.4%	66.3%	64.7%	71.5%	71.5%	71.5%	71.5%	71.5%	71.5%	71.5%	71.5%	71.0%	71.0%	71.3%	71.3%	75.2%	75.2%	74.8%	99.2%	99.2%	99.2%	99.2%	99.2%	99.1%	99.3%	99.3%	99.3%	99.3%	99.2%	99.3%
89. DUCK ADENOVIRUS...	48.7%	40.8%	39.3%	48.5%	48.5%	48.8%	48.8%	48.8%	48.8%	48.8%	48.8%	48.4%	48.2%	48.7%	51.5%	52.4%	52.4%	52.4%	48.6%	48.7%	48.7%	48.6%	48.6%	48.6%	49.0%	49.0%	49.0%	49.0%	48.6%	49.0%

	61. UDL...	62. UDL...	63. UDL...	64. UDL...	65. UDL...	66. UDL...	67. UDL...	68. UDL...	69. UDL...	70. UDL...	71. UDL...	72. UDL...	73. UDL...	74. DQ3...	75. KU7...	76. KP2...	77. MF1...	78. MN...	79. MK7...	80. KY2...	81. UDL...	82. MT1...	83. UDL...	84. UDL...	85. UDL...	86. UDL...	87. KT8...	88. MK4...	89. DU...
1. NC 015323.1 CANADA	61.3%	61.9%	61.9%	61.9%	63.0%	63.0%	62.2%	62.7%	63.0%	63.0%	63.0%	63.0%	62.7%	64.5%	70.5%	70.5%	62.2%	70.1%	70.2%	62.4%	63.6%	64.0%	63.7%	63.7%	63.7%	63.7%	70.5%	59.9%	50.6%
2. GU188428.1 CANADA	61.3%	61.9%	61.9%	61.9%	63.0%	63.0%	62.2%	62.7%	63.0%	63.0%	63.0%	63.0%	62.7%	64.5%	70.5%	70.5%	62.2%	70.1%	70.2%	62.4%	63.6%	64.0%	63.7%	63.7%	63.7%	63.7%	70.5%	59.9%	50.6%
3. KY636400.1 CHINA	61.6%	62.2%	62.2%	62.2%	63.1%	63.1%	62.3%	62.8%	63.1%	63.1%	63.1%	63.1%	62.8%	64.6%	70.5%	70.5%	62.4%	70.1%	70.2%	62.5%	63.4%	63.7%	63.5%	63.5%	63.5%	63.5%	70.3%	60.0%	50.7%
4. KX538980.1 CHINA	66.5%	67.4%	67.4%	67.4%	68.7%	68.7%	67.5%	68.2%	68.7%	68.7%	68.7%	68.7%	68.5%	70.2%	72.2%	72.2%	67.9%	71.7%	71.8%	68.2%	67.5%	68.0%	67.5%	67.5%	67.5%	67.5%	71.8%	69.5%	51.6%
5. KU558761.1 CHINA	61.6%	62.2%	62.2%	62.2%	63.1%	63.1%	62.3%	62.8%	63.1%	63.1%	63.1%	63.1%	62.8%	64.6%	70.5%	70.5%	62.4%	70.1%	70.2%	62.5%	63.4%	63.7%	63.5%	63.5%	63.5%	63.5%	70.3%	60.0%	50.7%
6. KU569296.1 CHINA	61.6%	62.2%	62.2%	62.2%	63.1%	63.1%	62.3%	62.8%	63.1%	63.1%	63.1%	63.1%	62.8%	64.6%	70.5%	70.5%	62.4%	70.1%	70.2%	62.5%	63.4%	63.7%	63.5%	63.5%	63.5%	63.5%	70.3%	60.0%	50.7%
7. MH454598.1 CHINA	61.6%	62.2%	62.2%	62.2%	63.1%	63.1%	62.3%	62.8%	63.1%	63.1%	63.1%	63.1%	62.8%	64.6%	70.5%	70.5%	62.4%	70.1%	70.2%	62.5%	63.4%	63.7%	63.5%	63.5%	63.5%	63.5%	70.3%	60.0%	50.7%
8. MN781666.1 CHINA	61.6%	62.2%	62.2%	62.2%	63.1%	63.1%	62.3%	62.8%	63.1%	63.1%	63.1%	63.1%	62.8%	64.6%	70.5%	70.5%	62.4%	70.1%	70.2%	62.5%	63.4%	63.7%	63.5%	63.5%	63.5%	63.5%	70.3%	60.0%	50.7%
9. HE608152.1 AUSTRIA	61.2%	61.8%	61.8%	61.8%	62.7%	62.7%	61.9%	62.5%	62.7%	62.7%	62.7%	62.7%	62.5%	64.3%	70.4%	70.4%	62.0%	70.0%	70.1%	62.2%	63.5%	63.8%	63.6%	63.6%	63.6%	63.6%	70.3%	59.6%	50.6%
10. MN542422.1 CHINA	61.2%	61.8%	61.8%	61.8%	62.7%	62.7%	61.9%	62.5%	62.7%	62.7%	62.7%	62.7%	62.5%	64.5%	70.7%	70.7%	62.0%	70.3%	70.4%	62.2%	63.5%	63.8%	63.6%	63.6%	63.6%	63.6%	70.6%	59.9%	50.9%
11. NC 001720.1 AUSTR...	64.5%	65.7%	65.7%	65.7%	66.7%	66.7%	65.8%	66.5%	66.7%	66.7%	66.7%	66.7%	66.5%	67.7%	72.0%	72.0%	65.7%	71.9%	72.1%	66.1%	66.4%	66.7%	66.6%	66.6%	66.6%	66.6%	72.0%	63.4%	51.5%
12. U46933.1 AUSTRIA	64.5%	65.7%	65.7%	65.7%	66.7%	66.7%	65.8%	66.5%	66.7%	66.7%	66.7%	66.7%	66.5%	67.7%	72.0%	72.0%	65.7%	71.9%	72.1%	66.1%	66.4%	66.7%	66.6%	66.6%	66.6%	66.6%	72.0%	63.4%	51.5%
13. KX247012.1 POLAND	64.5%	65.7%	65.7%	65.7%	66.7%	66.7%	65.8%	66.5%	66.7%	66.7%	66.7%	66.7%	66.5%	67.8%	72.1%	72.1%	65.9%	71.9%	72.2%	66.1%	66.7%	66.9%	66.8%	66.8%	66.8%	66.8%	72.1%	63.3%	51.7%
14. NC 021221.1 NORT...	68.6%	69.2%	69.2%	69.2%	69.9%	69.9%	69.3%	69.7%	69.9%	69.9%	69.9%	69.9%	69.8%	70.3%	75.5%	75.5%	69.1%	75.4%	75.5%	69.2%	70.2%	70.4%	70.5%	70.5%	70.5%	70.5%	75.6%	66.7%	51.3%
15. KC493646.1 NORTH...	68.6%	69.2%	69.2%	69.2%	69.9%	69.9%	69.3%	69.7%	69.9%	69.9%	69.9%	69.9%	69.8%	70.3%	75.5%	75.5%	69.1%	75.4%	75.5%	69.2%	70.2%	70.4%	70.5%	70.5%	70.5%	70.5%	75.6%	66.7%	51.3%
16. MG953201.1 HUNG...	69.7%	70.3%	70.3%	70.3%	71.0%	71.0%	70.4%	70.8%	71.0%	71.0%	71.0%	71.0%	70.9%	71.4%	75.9%	75.9%	70.2%	75.9%	75.9%	70.3%	70.9%	71.1%	71.1%	71.1%	71.1%	71.1%	75.9%	68.1%	51.0%
17. UDLA 71 ECUADOR 6	73.8%	73.9%	73.9%	73.9%	74.5%	74.5%	74.0%	74.4%	74.5%	74.5%	74.5%	74.5%	74.4%	74.3%	74.3%	74.3%	73.8%	74.4%	74.6%	74.0%	74.4%	74.4%	74.5%	74.5%	74.5%	74.5%	74.4%	71.5%	48.5%
18. MF161433.1 ECUAD...	73.7%	73.6%	73.6%	73.6%	73.8%	73.8%	73.7%	73.8%	73.8%	73.8%	73.8%	73.8%	73.8%	73.5%	73.5%	73.5%	73.5%	73.6%	73.9%	73.9%	73.6%	73.6%	73.8%	73.8%	73.8%	73.8%	73.6%	71.1%	47.9%
19. KT862808.1 AUSTRIA	73.9%	74.0%	74.0%	74.0%	74.7%	74.7%	74.1%	74.5%	74.7%	74.7%	74.7%	74.7%	74.5%	75.3%	78.2%	78.2%	73.9%	77.8%	77.9%	74.2%	74.8%	74.9%	74.9%	74.9%	74.9%	74.9%	78.2%	71.7%	50.9%
20. NC 038332.1 JAPAN	73.9%	74.0%	74.0%	74.0%	74.7%	74.7%	74.1%	74.5%	74.7%	74.7%	74.7%	74.7%	74.5%	75.3%	78.2%	78.2%	73.9%	77.8%	77.9%	74.2%	74.8%	74.9%	74.9%	74.9%	74.9%	74.9%	78.2%	71.7%	50.9%
21. NC 014969.1 CANA...	71.2%	71.5%	71.5%	71.5%	72.3%	72.3%	71.8%	72.1%	72.3%	72.3%	72.3%	72.3%	72.1%	72.9%	78.1%	78.1%	71.1%	77.8%	78.3%	71.6%	72.9%	73.1%	73.0%	73.0%	73.0%	73.0%	78.4%	68.4%	52.2%
22. GU734104.1 CANA...	71.2%	71.5%	71.5%	71.5%	72.3%	72.3%	71.8%	72.1%	72.3%	72.3%	72.3%	72.3%	72.1%	72.9%	78.1%	78.1%	71.1%	77.8%	78.3%	71.6%	72.9%	73.1%	73.0%	73.0%	73.0%	73.0%	78.4%	68.4%	52.2%
23. KX258422.1 PERU	71.3%	71.7%	71.7%	71.7%	72.5%	72.5%	71.8%	72.3%	72.5%	72.5%	72.5%	72.5%	72.3%	73.0%	77.8%	77.8%	71.2%	77.5%	77.9%	71.7%	73.0%	73.2%	73.1%	73.1%	73.1%	73.1%	78.1%	68.9%	51.7%
24. MK850433.1 IRAN	63.9%	63.9%	63.9%	63.9%	63.9%	63.9%	63.9%	63.9%	63.9%	63.9%	63.9%	63.9%	63.9%	63.9%	63.9%	63.9%	63.9%	63.9%	63.9%	63.9%	65.0%	65.0%	65.0%	65.0%	65.0%	65.0%	65.0%	63.9%	43.8%
25. KY229168.1 BRAZIL	71.1%	71.1%	71.1%	71.1%	71.1%	71.1%	71.1%	71.1%	71.1%	71.1%	71.1%	71.1%	71.1%	70.7%	70.7%	70.7%	70.7%	70.7%	71.1%	71.1%	71.3%	71.3%	71.4%	71.4%	71.4%	71.4%	71.3%	67.5%	49.2%
26. MT104454.1 INDON...	71.4%	71.8%	71.8%	71.8%	72.6%	72.6%	71.9%	72.4%	72.6%	72.6%	72.6%	72.6%	72.4%	72.4%	72.4%	72.4%	71.3%	72.6%	73.1%	71.8%	72.9%	73.0%	73.0%	73.0%	73.0%	73.0%	73.0%	69.0%	49.1%
27. KT862811.1 UNITE...	71.2%	71.5%	71.5%	71.5%	72.3%	72.3%	71.8%	72.1%	72.3%	72.3%	72.3%	72.3%	72.1%	72.9%	78.0%	78.0%	71.1%	77.7%	78.2%	71.6%	72.7%	72.9%	72.8%	72.8%	72.8%	72.8%	78.2%	68.7%	51.9%
28. KT862809.1 JAPAN	71.6%	72.2%	72.2%	72.2%	73.1%	73.1%	72.4%	72.9%	73.1%	73.1%	73.1%	73.1%	72.9%	73.7%	78.0%	78.0%	72.0%	77.8%	78.2%	72.4%	73.4%	73.5%	73.5%	73.5%	73.5%	73.5%	78.2%	70.0%	51.5%
29. KP879219.1 POLAND	66.9%	66.9%	66.9%	66.9%	66.9%	66.9%	66.9%	66.9%	66.9%	66.9%	66.9%	66.9%	66.9%	66.7%	66.7%	66.7%	66.7%	66.7%	67.1%	67.1%	67.2%	67.2%	67.2%	67.2%	67.2%	67.2%	67.2%	66.7%	44.3%
30. UDLA 4 ECUADOR 8a	73.5%	73.9%	73.9%	73.9%	74.6%	74.6%	74.1%	74.5%	74.6%	74.6%	74.6%	74.6%	74.5%	74.1%	74.1%	74.1%	73.7%	74.5%	74.7%	74.2%	74.4%	74.4%	74.5%	74.5%	74.5%	74.5%	74.4%	71.3%	48.9%
31. UDLA 17 ECUADOR	73.5%	73.9%	73.9%	73.9%	74.5%	74.5%	74.1%	74.4%	74.5%	74.5%	74.5%	74.5%	74.5%	74.0%	74.0%	74.0%	73.7%	74.4%	74.6%	74.2%	74.3%	74.3%	74.4%	74.4%	74.4%	74.4%	74.3%	71.4%	48.7%
32. MG008488.1 MORO...	66.3%	66.3%	66.3%	66.3%	66.3%	66.3%	66.3%	66.3%	66.3%	66.3%	66.3%	66.3%	66.3%	66.3%	66.3%	66.3%	66.3%	66.7%	66.5%	66.5%	66.7%	66.7%	66.7%	66.7%	66.7%	66.7%	66.7%	66.3%	40.8%
33. KT781516.1 EGYPT	64.7%	64.7%	64.7%	64.7%	64.7%	64.7%	64.7%	64.7%	64.7%	64.7%	64.7%	64.7%	64.7%	64.7%	64.7%	64.7%	64.7%	65.1%	64.9%	64.9%	64.9%	64.9%	64.9%	64.9%	64.9%	64.9%	64.9%	64.7%	39.3%
34. UDLA 310-2 ECUAD...	72.3%	72.6%	72.6%	72.6%	73.3%	73.3%	72.8%	73.1%	73.3%	73.3%	73.3%	73.3%	73.2%	72.8%	72.8%	72.8%	72.4%	73.2%	73.5%	73.3%	73.1%	73.1%	73.2%	73.2%	73.2%	73.2%	73.1%	71.5%	48.5%
35. UDLA 292-2 ECUAD...	72.3%	72.6%	72.6%	72.6%	73.3%	73.3%	72.8%	73.1%	73.3%	73.3%	73.3%	73.3%	73.2%	72.8%	72.8%	72.8%	72.4%	73.2%	73.5%	73.3%	73.1%	73.1%	73.2%	73.2%	73.2%	73.2%	73.1%	71.5%	48.5%
36. UDLA 282 ECUADOR	73.5%	73.9%	73.9%	73.9%	74.7%	74.7%	74.1%	74.5%	74.7%	74.7%	74.7%	74.7%	74.5%	74.3%	74.3%	74.3%	73.7%	74.7%	75.0%	74.2%	74.5%	74.5%	74.6%	74.6%	74.6%	74.6%	74.5%	71.5%	48.8%
37. UDLA 316 ECUADOR	73.5%	73.9%	73.9%	73.9%	74.7%	74.7%	74.1%	74.5%	74.7%	74.7%	74.7%	74.7%	74.5%	74.3%	74.3%	74.3%	73.7%	74.7%	75.0%	74.2%	74.5%	74.5%	74.6%	74.6%	74.6%	74.6%	74.5%	71.5%	48.8%
38. UDLA 310 ECUADOR	73.5%	73.9%	73.9%	73.9%	74.7%	74.7%	74.1%	74.5%	74.7%	74.7%	74.7%	74.7%	74.5%	74.3%	74.3%	74.3%	73.7%	74.7%	75.0%	74.2%	74.5%	74.5%	74.6%	74.6%	74.6%	74.6%	74.5%	71.5%	48.8%
39. UDLA 291 ECUADOR	73.5%	73.9%	73.9%	73.9%	74.7%	74.7%	74.1%	74.5%	74.7%	74.7%	74.7%	74.7%	74.5%	74.3%	74.3%	74.3%	73.7%	74.7%	75.0%	74.2%	74.5%	74.5%	74.6%	74.6%	74.6%	74.6%	74.5%	71.5%	48.8%
40. UDLA 292 ECUADOR	73.5%	73.9%	73.9%	73.9%	74.7%	74.7%	74.1%	74.5%	74.7%	74.7%	74.7%	74.7%	74.5%	74.3%	74.3%	74.3%	73.7%	74.7%	75.0%	74.2%	74.5%	74.5%	74.6%	74.6%	74.6%	74.6%	74.5%	71.5%	48.8%
41. UDLA 303 ECUADO...	73.5%	73.9%	73.9%	73.9%	74.7%	74.7%	74.1%	74.5%	74.7%	74.7%	74.7%	74.7%	74.5%	74.3%	74.3%	74.3%	73.7%	74.7%	75.0%	74.2%	74.5%	74.5%	74.6%	74.6%	74.6%	74.6%	74.5%	71.5%	48.8%
42. MN453821.1 BRAZI...	72.7%	73.1%	73.1%	73.1%	74.0%	74.0%	73.4%	73.8%	74.0%	74.0%	74.0%	74.0%	73.8%	74.6%	74.6%	74.6%	72.9%	75.1%	75.3%	73.4%	74.0%	74.1%	74.1%	74.1%	74.1%	74.1%	74.9%	71.0%	48.4%
43. KY229177.1 BRAZIL	72.7%	73.1%	73.1%	73.1%	74.0%	74.0%	73.4%	73.8%	74.0%	74.0%	74.0%	74.0%	73.8%	74.2%	74.2%	74.2%	72.9%	74.7%	74.9%	73.4%	74.0%	74.1%	74.1%	74.1%	74.1%	74.1%	74.4%	71.0%	48.2%
44. DQ323985.1 USA	73.2%	73.8%	73.8%	73.8%	74.6%	74.6%	74.0%	74.4%	74.6%	74.6%	74.6%	74.6%	74.4%	75.0%	75.0%	75.0%	73.5%	75.4%	75.7%	74.0%	74.5%	74.7%	74.7%	74.7%	74.7%	74.7%	75.2%	71.3%	48.7%
45. KT862810.1 JAPAN	73.0%	73.5%	73.5%	73.5%	74.4%	74.4%	73.8%	74.2%	74.4%	74.4%	74.4%	74.4%	74.2%	74.8%	78.7%	78.7%	73.3%	78.6%	79.0%	73.8%	74.3%	74.5%	74.4%	74.4%	74.4%	74.4%	79.0%	71.3%	51.5%
46. NC 000899.1 POLA...	78.5%	78.9%	78.9%	78.9%	78.8%	78.8%	78.6%	78.7%	78.8%	78.8%	78.8%	78.8%	78.7%	80.5%	80.3%	80.3%	79.1%	79.7%	79.8%	78.6%	78.6%	79.0%	78.5%	78.5%	78.5%	78.5%	80.2%	75.2%	52.4%
47. AF083975.2 CANADA	78.5%	78.9%	78.9%	78.9%	78.8%	78.8%	78.6%	78.7%	78.8%	78.8%	78.8%	78.8%	78.7%	80.5%	80.3%	80.3%	79.1%	79.7%	79.8%	78.6%	78.6%	79.0%	78.5%	78.5%	78.5%	78.5%	80.2%	75.2%	52.4%
48. KT862807.1 JAPAN	78.0%	78.5%	78.5%	78.5%	78.5%	78.5%	78.2%	78.3%	78.5%	78.5%	78.5%	78.5%	78.3%	79.5%	80.0%	80.0%	77.8%	79.7%	79.7%	78.0%	77.6%	77.9%	77.5%	77.5%	77.5%	77.5%	79.8%	74.8%	52.4%
49. UDLA 2 ECUADOR 11	99.9%	99.9%	99.9%	99.9%	99.9%	99.9%	99.9%	99.9%	99.9%	99.9%	99.9%	99.9%	99.9%	98.6%	98.6%	98.6%	98.6%	99.3%	99.9%	99.9%	94.8%	94.8%	94.8%	94.8%	94.8%	94.8%	94.8%	99.2%	48.6%
50. UDLA 3 ECUADOR	99.7%	99.8%	99.8%	99.8%	99.8%	99.8%	99.8%	99.8%	99.8%	99.8%	99.8%	99.8%	99.8%	98.8%	98.8%	98.8%	98.8%	99.1%	99.8%	99.7%	94.9%	94.9%	94.9%	94.9%	94.9%	94.9%	94.9%	99.2%	48.7%
51. UDLA 72 ECUADOR	99.7%	99.8%	99.8%	99.8%	99.8%	99.8%	99.8%	99.8%	99.8%	99.8%	99.8%	99.8%	99.8%	98.8%	98.8%	98.8%	98.8%	99.1%	99.8%	99.7%	94.9%	94.9%	94.9%	94.9%	94.9%	94.9%	94.9%	99.2%	48.7%
52. UDLA 76 ECUADOR	99.9%	99.9%	99.9%	99.9%	99.9%	99.9%	99.9%	99.9%	99.9%	99.9%	99.9%	99.9%	99.9%	98.6%	98.6%	98.6%	98.6%	99.3%	99.9%	99.9%	94.8%	94.8%	94.8%	94.8%	94.8%	94.8%	94.8%	99.2%	48.6%
53. UDLA 78 ECUADOR	99.9%	99.9%	99.9%	99.9%	99.9%	99.9%	99.9%	99.9%	99.9%	99.9%	99.9%	99.9%	99.9%	98.6%	98.6%	98.6%	98.6%	99.3%	99.9%	99.9%	94.8%	94.8%	94.8%	94.8%	94.8%	94.8%	94.8%	99.2%	48.6%
54. UDLA 80 ECUADOR	100%	99.9%	99.9%	99.9%	99.9%	99.9%	99.9%	99.9%	99.9%	99.9%	99.9%	99.9%	99.9%	98.4%	98.4%	98.4%	98.4%	99.0%	99.6%	99.6%	94.5%	94.5%	94.5%	94.5%	94.5%	94.5%	94.5%	99.1%	48.6%
55. UDLA 226 ECUADOR	100%	100%	100%	100%	100%	100%	100%	100%	100%	100%	100%	100%	100%	98.6%	98.6%	98.6%	98.5%	99.0%	99.8%	99.8%	94.9%	94.9%	94.9%	94.9%	94.9%	94.9%	94.9%	99.3%	49.0%
56. UDLA 222 ECUADOR	100%	100%	100%	100%	100%	100%	100%	100%	100%	100%	100%	100%	100%	98.6%	98.6%	98.6%	98.5%	99.0%	99.8%	99.8%	94.9%	94.9%	94.9%	94.9%	94.9%	94.9%	94.9%	99.3%	49.0%
57. UDLA 213 ECUADOR	100%	100%	100%	100%	100%	100%	100%	100%	100%	100%	100%	100%	100%	98.6%	98.6%	98.6%	98.5%	99.0%	99.8%	99.8%	94.9%	94.9%	94.9%	94.9%	94.9%	94.9%	94.9%	99.3%	49.0%
58. UDLA 210 ECUADOR	100%	100%	100%	100%	100%	100%	100%	100%	100%	100%	100%	100%	100%	98.6%	98.6%	98.6%	98.5%	99.0%	99.8%	99.8%	94.9%	94.9%	94.9%	94.9%	94.9%	94.9%	94.9%	99.3%	49.0%
59. UDLA 85 ECUADOR	100%	100%	100%	100%	100%	100%	100%	100%	100%	100%	100%	100%	100%	98.5%	98.5%	98.5%	98.5%	99.1%	99.8%	99.7%	94.7%	94.7%	94.7%	94.7%	94.7%	94.7%	94.7%	99.2%	48.6%
60. UDLA 250 ECUADOR	100%	100%	100%	100%	100%	100%	100%	100%	100%	100%	100%	100%	100%	98.6%	98.6%	98.6%	98.5%	99.0%	99.8%	99.8%	94.8%	94.8%	94.8%	94.8%	94.8%	94.8%	94.8%	99.3%	49.0%
61. UDLA 86-2 ECUADOR		100%	100%	100%	100%	100%	100%	100%	100%	100%	100%	100%	100%	98.4%	98.4%	98.4%	98.4%	99.1%	99.7%	99.7%	94.4%	94.4%	94.4%	94.4%	94.4%	94.4%	94.4%	99.2%	47.7%
62. UDLA 88 ECUADOR	100%		100%	100%	100%	100%	100%	100%	100%	100%	100%	100%	100%	98.5%	98.5%	98.5%	98.5%	99.1%	99.8%	99.7%	94.7%	94.7%	94.7%	94.7%	94.7%	94.7%	94.7%	99.2%	48.6%
63. UDLA 207 ECUADOR	100%	100%		100%	100%	100%	100%	100%	100%	100%	100%	100%	100%	98.5%	98.5%	98.5%	98.5%	99.1%	99.8%	99.7%	94.7%	94.7%	94.7%	94.7%	94.7%	94.7%	94.7%	99.2%	48.6%
64. UDLA 76F ECUADOR	100%	100%	100%		100%	100%	100%	100%	100%	100%	100%	100%	100%	98.5%	98.5%	98.5%	98.5%	99.1%	99.8%	99.7%	94.7%	94.7%	94.7%	94.7%	94.7%	94.7%	94.7%	99.2%	48.6%
65. UDLA 276 ECUADOR	100%	100%	100%	100%		100%	100%	100%	100%	100%	100%	100%	100%	98.6%	98.6%	98.6%	98.5%	99.0%	99.8%	99.8%	94.9%	94.9%	94.9%	94.9%	94.9%	94.9%	94.9%	99.3%	49.1%
66. UDLA 268 ECUADOR	100%	100%	100%	100%	100%		100%	100%	100%	100%	100%	100%	100%	98.6%	98.6%	98.6%	98.5%	99.0%	99.8%	99.8%	94.9%	94.9%	94.9%	94.9%	94.9%	94.9%	94.9%	99.3%	49.1%
67. UDLA 257 ECUADOR	100%	100%	100%	100%	100%	100%		100%	100%	100%	100%	100%	100%	98.5%	98.5%	98.5%	98.5%	99.0%	99.8%	99.8%	94.7%	94.7%	94.7%	94.7%	94.7%	94.7%	94.7%	99.2%	48.5%
68. UDLA 247 ECUADOR	100%	100%	100%	100%	100%	100%	100%		100%	100%	100%	100%	100%	98.6%	98.6%	98.6%	98.5%	99.0%	99.8%	99.8%	94.8%	94.8%	94.8%	94.8%	94.8%	94.8%	94.8%	99.3%	49.0%
69. UDLA 267 ECUADOR	100%	100%	100%	100%	100%	100%	100%	100%		100%	100%	100%	100%	98.6%	98.6%	98.6%	98.5%	99.0%	99.8%	99.8%	94.9%	94.9%	94.9%	94.9%	94.9%	94.9%	94.9%	99.3%	49.1%
70. UDLA 280 ECUADOR	100%	100%	100%	100%	100%	100%	100%	100%	100%		100%	100%	100%	98.6%	98.6%	98.6%	98.5%	99.0%	99.8%	99.8%	94.9%	94.9%	94.9%	94.9%	94.9%	94.9%	94.9%	99.3%	49.1%
71. UDLA 299 ECUADOR	100%	100%	100%	100%	100%	100%	100%	100%	100%	100%		100%	100%	98.6%	98.6%	98.6%	98.5%	99.0%	99.8%	99.8%	94.9%	94.9%	94.9%	94.9%	94.9%	94.9%	94.9%	99.3%	49.1%
72. UDLA 86 ECUADOR	100%	100%	100%	100%	100%	100%	100%	100%	100%	100%	100%		100%	98.6%	98.6%	98.6%	98.5%	99.0%	99.8%	99.8%	94.9%	94.9%	94.9%	94.9%	94.9%	94.9%	94.9%	99.3%	49.1%
73. UDLA 203 ECUADO...	100%	100%	100%	100%	100%	100%	100%	100%	100%	100%	100%	100%		98.6%	98.6%	98.6%	98.5%	99.0%	99.8%	99.8%	94.8%	94.8%	94.8%	94.8%	94.8%	94.8%	94.8%	99.3%	49.0%
74. DQ323984.1 USA 11	98.4%	98.5%	98.5%	98.5%	98.6%	98.6%	98.5%	98.6%	98.6%	98.6%	98.6%	98.6%	98.6%		100%	100%	100%	98.8%	98.8%	98.9%	95.8%	96.0%	95.7%	95.7%	95.7%	95.7%	96.1%	100%	48.6%
75. KU746335.1 MEXIC...	98.4%	98.5%	98.5%	98.5%	98.6%	98.6%	98.5%	98.6%	98.6%	98.6%	98.6%	98.6%	98.6%	100%		100%	100%	98.5%	98.4%	98.9%	95.8%	96.0%	95.7%	95.7%	95.7%	95.7%	98.3%	100%	52.2%
76. KP231537.1 CANADA	98.4%	98.5%	98.5%	98.5%	98.6%	98.6%	98.5%	98.6%	98.6%	98.6%	98.6%	98.6%	98.6%	100%	100%		100%	98.5%	98.4%	98.9%	95.8%	96.0%	95.7%	95.7%	95.7%	95.7%	98.3%	100%	52.2%
77. MF161434.1 ECUAD...	98.4%	98.5%	98.5%	98.5%	98.5%	98.5%	98.5%	98.5%	98.5%	98.5%	98.5%	98.5%	98.5%	100%	100%	100%		98.8%	98.8%	98.9%	95.6%	95.7%	95.5%	95.5%	95.5%	95.5%	95.7%	100%	48.5%
78. MN428137.1 PAKIS...	99.1%	99.1%	99.1%	99.1%	99.0%	99.0%	99.0%	99.0%	99.0%	99.0%	99.0%	99.0%	99.0%	98.8%	98.5%	98.5%	98.8%		99.3%	99.3%	94.9%	94.9%	94.9%	94.9%	94.9%	94.9%	97.1%	99.3%	52.0%
79. MK757569.1 IRAN	99.7%	99.8%	99.8%	99.8%	99.8%	99.8%	99.8%	99.8%	99.8%	99.8%	99.8%	99.8%	99.8%	98.8%	98.4%	98.4%	98.8%	99.3%		100%	95.1%	95.2%	95.1%	95.1%	95.1%	95.1%	97.0%	99.4%	52.1%
80. KY229175.1 BRAZIL	99.7%	99.7%	99.7%	99.7%	99.8%	99.8%	99.8%	99.8%	99.8%	99.8%	99.8%	99.8%	99.8%	98.9%	98.9%	98.9%	98.9%	99.3%	100%		95.1%	95.1%	95.1%	95.1%	95.1%	95.1%	95.1%	99.4%	48.9%
81. UDLA 50 ECUADOR 2	94.4%	94.7%	94.7%	94.7%	94.9%	94.9%	94.7%	94.8%	94.9%	94.9%	94.9%	94.9%	94.8%	95.8%	95.8%	95.8%	95.6%	94.9%	95.1%	95.1%		99.9%	99.8%	99.8%	99.8%	99.8%	99.9%	95.1%	49.0%
82. MT104458.1 INDON...	94.4%	94.7%	94.7%	94.7%	94.9%	94.9%	94.7%	94.8%	94.9%	94.9%	94.9%	94.9%	94.8%	96.0%	96.0%	96.0%	95.7%	94.9%	95.2%	95.1%	99.9%		99.8%	99.8%	99.8%	99.8%	100%	95.1%	48.9%
83. UDLA 337 ECUADOR	94.4%	94.7%	94.7%	94.7%	94.9%	94.9%	94.7%	94.8%	94.9%	94.9%	94.9%	94.9%	94.8%	95.7%	95.7%	95.7%	95.5%	94.9%	95.1%	95.1%	99.8%	99.8%		100%	100%	100%	99.8%	94.9%	49.0%
84. UDLA 64 ECUADOR	94.4%	94.7%	94.7%	94.7%	94.9%	94.9%	94.7%	94.8%	94.9%	94.9%	94.9%	94.9%	94.8%	95.7%	95.7%	95.7%	95.5%	94.9%	95.1%	95.1%	99.8%	99.8%	100%		100%	100%	99.8%	94.9%	49.0%
85. UDLA 56 ECUADOR	94.4%	94.7%	94.7%	94.7%	94.9%	94.9%	94.7%	94.8%	94.9%	94.9%	94.9%	94.9%	94.8%	95.7%	95.7%	95.7%	95.5%	94.9%	95.1%	95.1%	99.8%	99.8%	100%	100%		100%	99.8%	94.9%	49.0%
86. UDLA 44 ECUADOR 2	94.4%	94.7%	94.7%	94.7%	94.9%	94.9%	94.7%	94.8%	94.9%	94.9%	94.9%	94.9%	94.8%	95.7%	95.7%	95.7%	95.5%	94.9%	95.1%	95.1%	99.8%	99.8%	100%	100%	100%		99.8%	94.9%	49.0%
87. KT862805.1 UNITE...	94.4%	94.7%	94.7%	94.7%	94.9%	94.9%	94.7%	94.8%	94.9%	94.9%	94.9%	94.9%	94.8%	96.1%	98.3%	98.3%	95.7%	97.1%	97.0%	95.1%	99.9%	100%	99.8%	99.8%	99.8%	99.8%		95.1%	52.2%
88. MK468898.1 MORO...	99.2%	99.2%	99.2%	99.2%	99.3%	99.3%	99.2%	99.3%	99.3%	99.3%	99.3%	99.3%	99.3%	100%	100%	100%	100%	99.3%	99.4%	99.4%	95.1%	95.1%	94.9%	94.9%	94.9%	94.9%	95.1%		46.3%
89. DUCK ADENOVIRUS...	47.7%	48.6%	48.6%	48.6%	49.1%	49.1%	48.5%	49.0%	49.1%	49.1%	49.1%	49.1%	49.0%	48.6%	52.2%	52.2%	48.5%	52.0%	52.1%	48.9%	49.0%	48.9%	49.0%	49.0%	49.0%	49.0%	52.2%	46.3%	
